# Juvenile hormone-mediated chromatin remodelling by cumulative action of downstream transcription factors, Hairy and Krüppel homolog 1

**DOI:** 10.1038/s42003-026-10327-4

**Published:** 2026-05-26

**Authors:** Roumik Banerjee, Challiyil S. Jayapriya, Ajeet K. Mohanty, Alexander S. Raikhel, Tusar T. Saha

**Affiliations:** 1 Department of Biological Sciences, Birla Institute of Technology and Science, Pilani, K K Birla Goa Campus, Sancoale, Goa India; 2 ICMR-National Institute of Malaria Research, Field Station, Panaji, Goa India; 3 Department of Entomology, University of California, Riverside, CA USA

**Keywords:** Chromatin remodelling, Gene regulation, Entomology

## Abstract

Insect-specific juvenile hormone (JH) controls mosquito post-eclosion (PE) development, a stage crucial for subsequent blood meal-driven egg maturation. During PE, JH functions through the independent or cumulative actions of repressors Hairy and Krüppel homolog 1 (Kr-h1), initiating the downregulation of different target gene cohorts. A key factor governing candidate gene expression is its chromatin state, determined by the tightness of DNA wrapping around associated proteins. Chromatin remodeling alters DNA accessibility and impacts gene expression. Despite advances in our understanding of the JH signaling pathway, little is known about JH/Met-induced chromatin remodeling during gene repression. Using the Dengue mosquito *Aedes aegypti*, we show that repressors Hairy and Kr-h1 induce alternative chromatin modifications to repress their target genes. While Hairy causes widespread histone H4 deacetylation of target genes, Kr-h1 induces histone H3K9 trimethylation and chromatin compaction. For the candidate genes co-repressed by both Hairy and Kr-h1, the two factors have a cumulative effect on the chromatin modification of the target gene loci, downregulating their expression. Our findings shed light on the mechanism behind the cumulative action of transcriptional repressors Hairy and Kr-h1 involved in JH signal transduction.

## Abstract (web summary)

Characterization of Juvenile hormone mediated chromatin remodeling of repressed genes in mosquito *Aedes aegypti* provide mechanistic insights into the cumulative action of downstream transcription factors, Hairy and Krüppel homolog 1.

## Introduction

The *Aedes aegypti* mosquito is a dangerous vector that transmits human viral diseases such as Dengue fever, yellow fever, Chikungunya, and Zika virus^1^. In adult female mosquitoes, vertebrate blood serves as a source of nutrients required for vitellogenesis, the process of production of yolk protein precursors (YPPs) in the fat body (FB), an insect organ analogous to vertebrate liver and adipose tissue. Vitellogenesis is primarily regulated by the steroid hormone 20-hydroxyecdysone (20E)^2,3^. Before initiating vitellogenesis, a mosquito undergoes a reproductive maturation necessary for the competence of blood-meal-driven events. In the newly eclosed mosquito, this period lasts about 72 hours and is termed the post-eclosion (PE) phase. This phase is controlled by juvenile hormone (JH) III, the only form of this hormone found in *Ae. aegypti* (hereafter referred to as JH)^4,5^. The JH level starts increasing after adult eclosion, reaching a peak at about 48-54 h PE^6^.

JH is a sesquiterpenoid growth regulator that is found only in arthropods^7,8^ and controls various biological processes, including development, reproduction, and metamorphosis^2,7^. This hormone functions through its receptor Methoprene-tolerant (Met), a member of the basic helix–loop–helix (bHLH)–Per-Arnt-Sim (PAS) family of proteins^8–12^. In the cell, Met is either present as a homodimer (Met-Met) or in association with the heat shock protein 83 (Hsp83) chaperon complex^13^. Upon JH binding, Met dissociates and heterodimerizes with another bHLH-PAS domain protein, Taiman (Tai), an insect homolog of the vertebrate steroid receptor co-activator (SRC)^7,14^. This ligand-bound heterodimer complex then binds to specific DNA elements (JH response elements, JHREs) through a core E-box motif in the promoter region of target genes^14,15^. This direct role of JH-Met in the activation of target genes has been confirmed for multiple candidates, including *early trypsin (ET), Hairy*, *Krüppel-homolog 1 (Kr-h1)*, and the *regulator of ribosomal synthesis 1 (RRS1)*^15–18^.

Unlike Met-mediated gene activation, repression mediated by Met is indirect, requiring the involvement of additional intermediate factors.^17,18^. Previously, the bHLH-orange domain protein Hairy and the C2H2 zinc-finger protein Kr-h1 were identified as Met-induced downstream repressors of the JH signaling pathway^6,14,15^. Hairy is demonstrated to bind E-box elements in the promoter of JH-Met targets and recruit co-factor Groucho, resulting in gene repression. The role of Kr-h1 as a JH pathway component and a repressor have been extensively researched during insect metamorphosis and during adult development in mosquito, *Ae. aegypti*. A consensus Kr-h1 binding element has been determined from various insects and validated using robust molecular techniques. Recently, these two factors have also been demonstrated to work together in the repression of a subset of JH-Met regulated genes. Such targets harbor both Hairy and Kr-h1 binding sites in their promoters responsible for recruiting these two factors resulting in cumulative trans-repression of the gene.^19^.

Gene regulation is a highly complex process that needs coordination among multiple components. The chromatin state defines a particular gene’s expression status at a given time and in a particular tissue. DNA molecules are wrapped around histone proteins, forming nucleosomes that condense into chromatin. Chromatin modifiers change the chromatin state, permitting the accessibility of transcription factors to DNA, thereby inducing activation or repression of target genes. ATP-dependent chromatin remodeling complexes alter histone occupancy in target genes by removing, incorporating, or restructuring nucleosomes^20^. Additionally, enzyme-based histone tail modifications, such as acetylation, methylation, phosphorylation, and ubiquitination, affect the binding affinity of DNA and histones, in turn, loosening or tightening the DNA contact with histones^21,22^. Acetylation by histone acetyltransferases (HATs) unwinds the DNA around the nucleosome, making it accessible to the transcriptional machinery. In contrast, histone deacetylases (HDACs) reverse this process, condensing the chromatin and leading to gene repression^23^. Histone methyl transferases (HMTs) are yet another class of histone-modifying enzymes that methylate specific lysine or arginine residues on histone tails. Based on the position of a residue, histone tail methylation may lead to either activation or repression of a target gene. For instance, H3K4 and H3K36 methylation are correlated with gene activation, whereas H3K9 and H3K27 methylation are modifications that lead to the repression of genes^24,25^. Many key transcription factors function by recruiting these nucleosome modifiers and altering the chromatin state of the target genes.

While JH-Met target genes have been identified in several insects, the chromatin status of their loci at different developmental points before and after JH action remained unexplored. Consequently, how JH may alter the chromatin structure, impacting the expression of target genes, is not completely understood. Homologs of JH-regulated proteins Hairy and Kr-h1 are known to recruit nucleosome remodelers in *Drosophila* and humans^26–29^. In this study, we investigated the role of JH-Met and its downstream factors, Hairy and Kr-h1, in the chromatin remodeling of JH-repressed genes in the adult female *Ae. aegypti* mosquito. We show that the JH-Met-Hairy axis induces widespread histone H4 deacetylation without triggering chromatin compaction of target genes. In contrast, the action of the JH-Met-Kr-h1 axis results in H3K9 trimethylation and chromatin compaction of their targets. Importantly, we demonstrate that for genes synergistically regulated by both transcription factors, JH-Met mediated histone H4 deacetylation was regulated by Hairy. At the same time, Kr-h1 was responsible for histone H3K9 trimethylation and chromatin compaction by the hormone. Apart from shedding light on the chromatin architecture of genes involved in mosquito reproduction, these results may hold the key to understanding the cumulative mode of action of Hairy and Kr-h1 downstream of JH-Met.

## Results

### Effect of JH on the histone H3 occupancy of genes repressed by Met-Hairy and Met-Kr-h1 axis

Previously, we characterized Hairy and Kr-h1 target genes downstream of JH-Met during PE development in adult female mosquito *Ae. aegypti*. Effect of JH-Met, the role of the intermediate factors, and the involvement of other co-factors in the repression of these target genes were studied in great detail. Furthermore, Hairy and Kr-h1 binding sites were identified computationally and thoroughly validated via electrophoretic mobility shift assay (EMSA), chromatin immunoprecipitation (ChIP), and cell culture-based Luciferase reporter assays. To investigate JH-mediated chromatin remodeling, we majorly focused on two key genes, *Ornithine decarboxylase* (*AAEL000044*, *ODC*) regulated by Hairy, and *Dimethylaniline monooxygenase* (*AAEL000834*, *DMO*) regulated by Kr-h1. Temporally, both these target genes show a high expression right after adult eclosion (0-4 h and 12-16 h PE), with intermediate expression levels observed around 24-28 h PE and gradually dropping to low expression during late PE (36-40 h, 48-52 h and 68-72 h) (see Supplementary Fig. S1A). As expected, the expression trend has an inverse correlation with JH titer, which low in newly eclosed mosquitoes and gradually increases to reach its peak around 48-60 h PE. While Hairy binding to the *ODC* promoter was demonstrated, the critical evidence of Kr-h1 interacting with its cognate binding elements specifically from *DMO* was missing. An octamer (GAAATAGA) was previously recognized as a potential Kr-h1 binding site (KBS), nearly 3 kb upstream of the *DMO* gene, based on in-silico bioinformatics analysis^19^. Using a cell culture-based approach and available anti-Kr-h1 antibody we performed ChIP assays. A clear enrichment in the immunoprecipitation (IP) signal was observed when previously cloned *Ae. aegypti* Kr-h1^19^ and cloned promoter was co-transfected in *Drosophila* S2 cells (see Supplementary Fig. S1B).

**Fig. 1:**
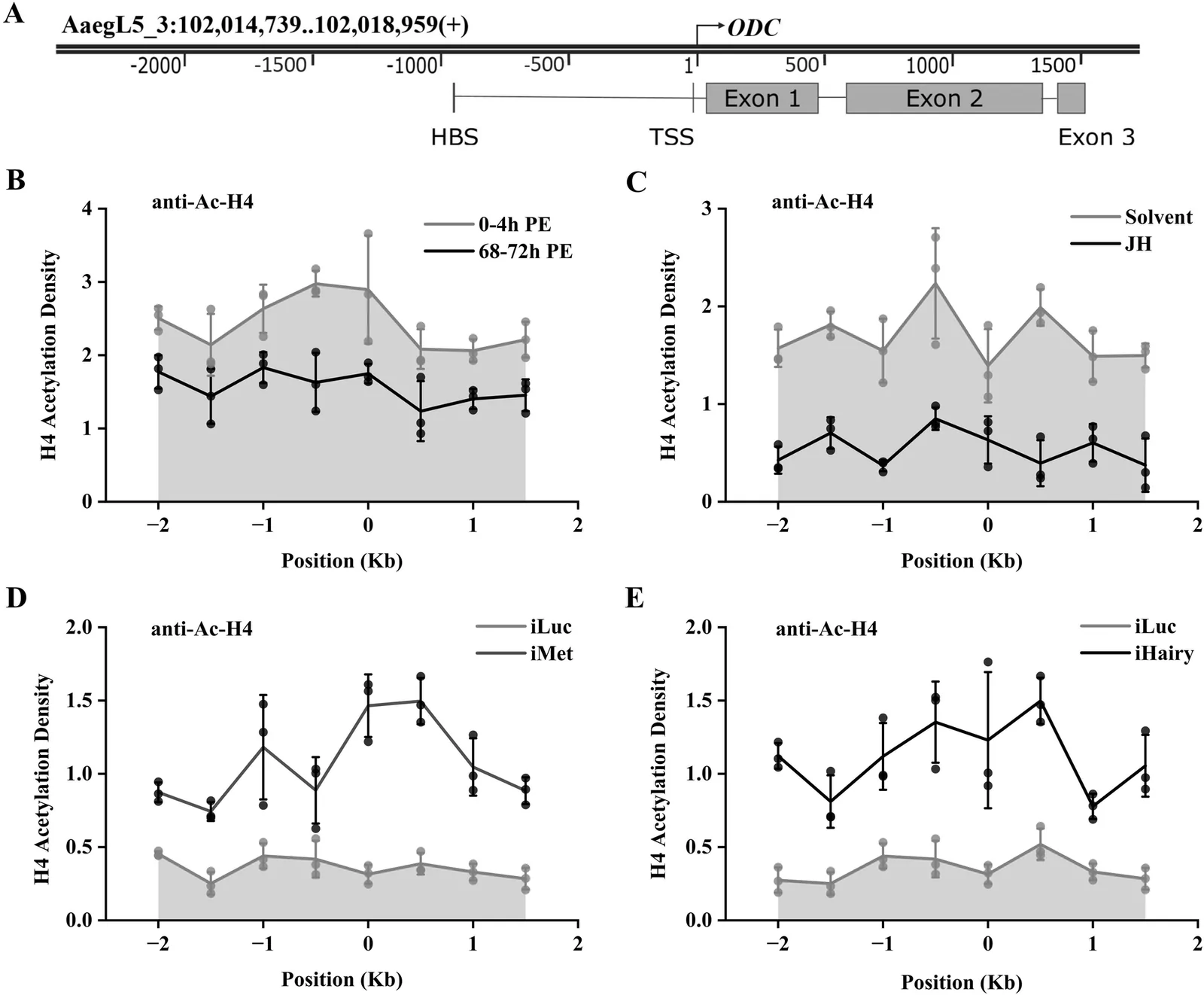
Hairy induces global changes in histone H4 acetylation of target gene *ODC.* **A** Schematic representation of target region within the *ODC* gene locus and HBS. **B** A lower level of histone H4-Ac is observed at 72 h PE than in newly eclosed mosquitoes (4 h PE) at all the gene loci assayed. **C** Similar lower global histone H4-Ac levels of the target gene were observed when 4 h PE mosquitoes were treated with exogenous JH. **D** dsRNA-mediated knockdown of JH receptor *Met* results in a compromised H4-Ac profile of the target gene. **E** Knockdown of intermediate factor *Hairy* phenocopies the effect of Met depletion, indicating the role of Hairy in chromatin compaction of genes repressed by JH/Met. For ectopic JH treatment, sample collection was done 6 h post treatment. For knockdown, dsRNA injection was performed on 24 h PE adult female mosquitoes followed by sample collection 48 h post injection. The H4-Ac density was measured by ChIP assay using anti-H4-Ac antibody. H4-Ac density was calculated from ChIP samples by normalizing the immunoprecipitation signal first with the H4-Ac level at the actin promoter and then with the relative H3 level. Error bars represent ± SD (n = 3). Three biological replicates were performed for all experiments with similar results. Source data are provided in Supplementary Data 1.

**Fig. 2:**
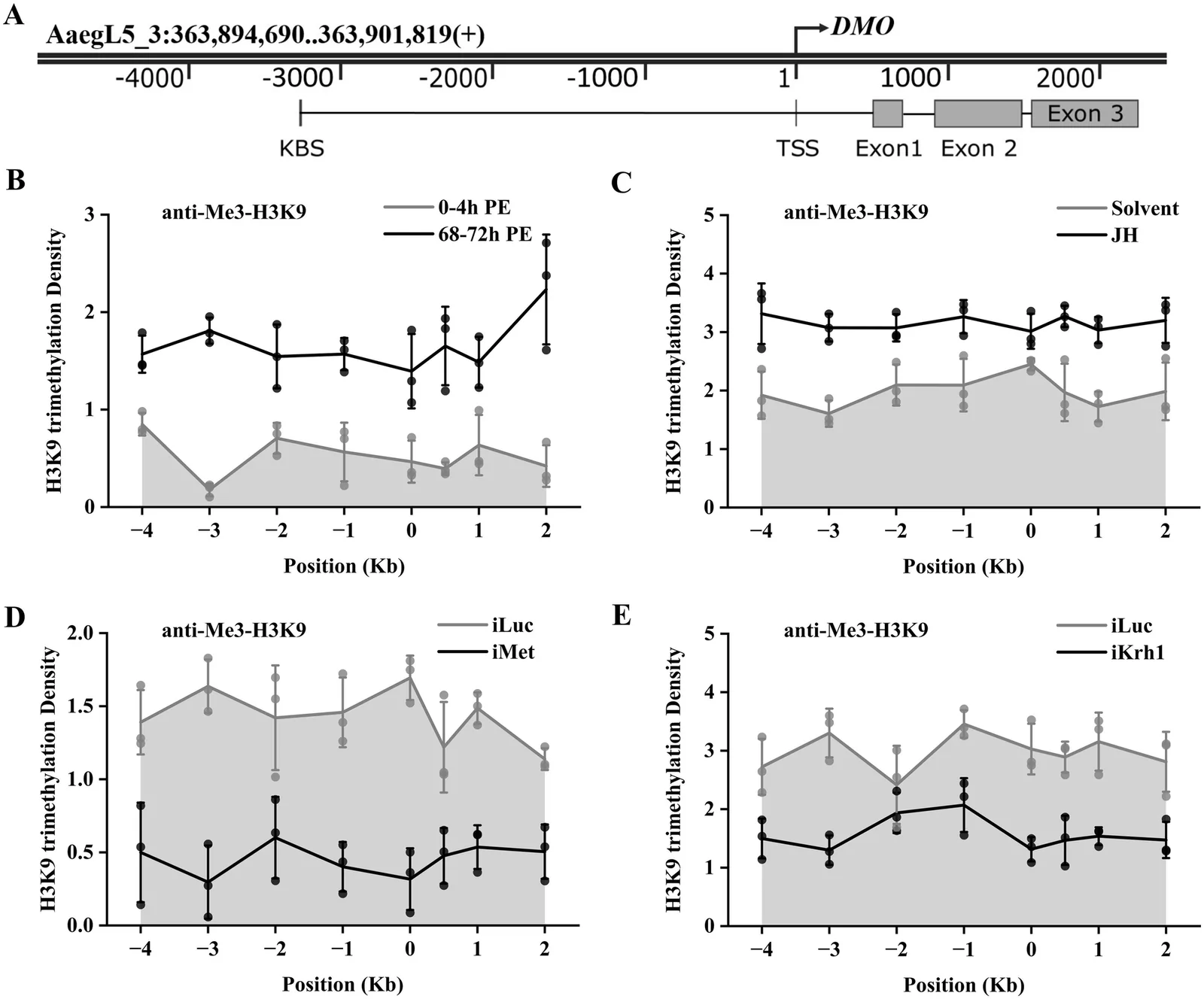
Kr-h1 induces long-range histone H3K9-Me3 of target gene *DMO.* **A** Schematic representation of target region within the *DMO* gene locus along with KBS. **B** Significantly higher H3K9-Me3 marks are observed at 72 h PE than in newly eclosed mosquitoes (4 h PE). **C** Widespread higher H3K9-Me3 density is also recorded with exogenous JH application at 4 h PE. **D** dsRNA-mediated knockdown of *Met* results in lower trimethylation levels in H3K9 than in control iLuc. **E** A similar result is observed after the knockdown of intermediate factor *Kr-h1* as a lower H3K9-Me3 density is observed in iKr-h1 than in iLuc samples. For ectopic JH treatment, sample collection was done 6 h post treatment. For knockdown, dsRNA injection was performed on 24 h PE adult female mosquitoes followed by sample collection 48 h post injection. The H3K9-Me3 density of target gene *DMO* was measured by ChIP assay using anti-H3K9-Me3 antibody for all experiments. H3K9-Me3 density was calculated from ChIP samples by normalizing the immunoprecipitation signal first with the H3K9-Me3 level at the actin promoter and then with the relative H3 level. Error bars represent ± SD (n = 3). Three biological replicates were performed for all experiments with similar results. Source data are provided in Supplementary Data 1.

To investigate the impact of the JH/Met signaling pathway on histone H3 occupancy of *ODC* and *DMO*, we used a ChIP assay with a rabbit polyclonal histone H3 antibody and quantitative polymerase chain reaction (qPCR) analysis. For quantification, primer pairs targeting eight loci—four in the promoter region (-2, -1.5, -1, and -0.5 kb) including the Hairy binding site (HBS), one harboring the TSS (0 kb), and three in the coding region (0.5, 1, and 1.5 kb)—of the *ODC* gene were used (see Supplementary Table S1). Similarly, primer pairs targeting eight loci—four in the promoter region (-4, -3, -2, and -1 kb) including the KBS, one harboring the TSS (0 kb), and three in the coding region (0.5, 1, and 2 kb) — of the *DMO* gene were used (see Supplementary Table S1). Gene maps for *ODC* harboring HBS and *DMO* harboring KBS at their respective promoter regions are provided for reference (Fig. 1A and 2A). We analyzed the difference in the histone H3 occupancy levels of both genes in the FB tissue at two developmental time points—4 h PE (JH low titer) and 72 h PE (JH high titer). No significant change in histone H3 occupancy was observed between these time points for most of the loci tested for the two genes (see Supplementary Fig. S2A and S3A, Table S2 and S3). Topical application of a physiological dosage of JH at 4 h PE had no impact on the histone H3 occupancy of either gene, indicating that JH does not alter the histone H3 occupancy map of its target genes (see Supplementary Fig. S2B and S3B, Table S2 and S3). In addition, dsRNA-mediated *Met* knockdown (iMet) did not impact the histone H3 occupancy map of either *ODC* or *DMO* (see Supplementary Fig. S2C and S3C, Table S2 and S3). dsRNA-mediated knockdowns of either *Hairy* or *Kr-h1* did not alter the H3 occupancy of *ODC* and *DMO*, respectively (see Supplementary Fig. S2D and S3D, Table S2 and S3). Taken together, we conclude that JH and its signaling pathway components do not alter the histone H3 occupancy of their target genes. Therefore, it can be assumed that gene repression by JH involves a different chromatin modification mechanism.

### Hairy mediates JH-induced widespread histone H4 deacetylation of target genes

Acetylation of the histone tail is strongly correlated with active genetic loci in eukaryotes, whereas histone deacetylation serves as a potential marker for repressed genes^22,23^. To characterize the effect of JH on the acetylation status of target genes, we performed ChIP assays with anti-histone H4 acetyl antibody targeting multiple lysine residues: Lys5, Lys8, Lys12 and Lys16. *ODC* and *DMO* were assayed via qPCR using the primer pairs described in the previous section. The overall experimental design was the same as described above. For the Met-Hairy-regulated gene, *ODC*, a clearly lower histone H4 acetylation (H4-Ac) was observed at 72 h PE than at 4 h PE for most of the assayed loci (Fig. 1B, Supplementary Table S2). In contrast, such a decrease in H4-Ac density was not observed for the Met-Kr-h1-regulated gene, *DMO* (see Supplementary Fig. S4A, Table S3). Topical JH application on 4 h PE mosquito abdomens also had distinct effects on the acetylation status of the two target genes, *ODC* and *DMO*. JH action resulted in a 2- to 4-fold lower H4-Ac level of *ODC* at all the loci assayed (Fig. 1C, Supplementary Table S2). In the case of *DMO*, however, JH did not alter the H4-Ac levels of any assayed loci (see Supplementary Fig. S4B, Table S3). Next, we checked the impact of JH receptor knockdown on the acetylation status of *ODC* and *DMO*. In iMet mosquito FB, an opposite effect to that of the JH application was observed for *ODC*. A dramatically higher H4-Ac density was recorded for most of the loci tested compared to iLuc controls (Fig. 1D, Supplementary Table S2). For *DMO*, no change was detected in any of the loci (see Supplementary Fig. S4C, Table S3). Based on these results, we can conclude that JH-Met differentially impacts the H4-Ac density of its target genes, *ODC* and *DMO*. While JH-Met induces widespread histone H4 deacetylation of *ODC* (a gene regulated by the JH-Met-Hairy axis), JH-Met-Kr-h1-regulated *DMO* acetylation status remains unaltered by the hormone action.

To test the role of Hairy and Kr-h1 in altering the histone H4-Ac levels of target genes, we performed the dsRNA-mediated knockdown of these two factors in the female mosquito’s PE phase (24 h PE). FB tissue samples were collected 48 hours post injection and were subjected to ChIP assays, as mentioned above. The iHairy sample shows a dramatically higher histone H4-Ac density across most of the loci of the *ODC* gene, a result similar to that of iMet (Fig. 1E, Supplementary Table S2). Ectopic treatment of iHairy mosquitoes with JH did not result in histone H4 deacetylation characteristically observed in iLuc+JH controls (see Supplementary Fig. S5, Table S2). Hormonal treatments were done at 48 h post injection and were incubated for 6 h followed by tissue collection for ChIP assays with the desired antibody. These results indicate that JH-Met induces the widespread histone H4 deacetylation of *ODC* through Hairy. In contrast, the knockdown of *Kr-h1* had no impact on the H4-Ac levels of various loci of the *DMO* gene, thus further confirming that the JH-Met-Kr-h1 axis does not alter histone H4-Ac (see Supplementary Fig. S4D, Table S3).

Similar results were obtained for two other candidate genes- *Matrix metalloproteinase (AAEL002655, MMP*) and *Thioester-containing protein 3 (AAEL008607, Tep3)* controlled by Hairy and Kr-h1, respectively. For quantification, primer pairs targeting four loci—two in the promoter and two in the coding region of the *MMP* gene, and primer pairs targeting four loci—two in the promoter, one harboring the TSS, and one in the coding region of the *Tep3* gene were used (see Supplementary Table S1). While JH promotes widespread H4 deacetylation of the *MMP* gene through Hairy (see Supplementary Fig. S6A-C, Table S5), it fails to alter the H4 acetylation density of *Tep3* (see Supplementary Fig. S7A-C, Table S6). In *Drosophila*, Hairy induces deacetylation of the histone H4 tail of its target gene, *Fushi tarazu* (*ftz*), during embryo development^30^.

### JH promotes long-range histone H3K9 trimethylation via Kr-h1

Histone methylation at specific residues of the histone tail is directly correlated with gene repression^24^. H3K9 trimethylation (H3K9-Me3) is an important marker in studying the chromatin status of repressed genes. To understand the impact of JH on H3K9-Me3 during target gene repression, ChIP assays were performed using an anti-histone H3K9-Me3 antibody, keeping the rest of the experimental design the same as before. We observed no significant change in histone H3K9-Me3 density on any loci of the *ODC* gene during PE development (4 h and 72 h PE) (see Supplementary Fig. S8A, Table S2). Neither JH’s ectopic application nor Met’s knockdown altered the H3K9-Me3 density of *ODC* (see Supplementary Fig. S8B and S8C, Table S2). Furthermore, Hairy, the DNA-binding transcriptional repressor of *ODC*, did not alter its H3K9-Me3 density (see Supplementary Fig. S8D, Table S2). We conclude that the JH-Met-Hairy axis does not affect the H3K9-Me3 status of target genes during its repression. In contrast, the other JH target gene, *DMO*, showed significantly elevated H3K9-Me3 marks across most of the loci we assayed during PE development (72 h PE) when compared with newly eclosed female mosquitoes (4 h PE) (Fig. 2B, Supplementary Table S3). A widespread increase in H3K9-Me3 density was detected with ectopic JH application on newly eclosed female mosquitoes (4 h PE) for *DMO* (Fig. 2C, Supplementary Table S3). *Met* knockdown had the opposite effect to that of JH—all the loci of *DMO* in iMet mosquitoes demonstrated dramatically lower H3K9-Me3 levels than in the iLuc control (Fig. 2D, Supplementary Table S3). As already mentioned, *DMO* is a gene regulated by JH-Met via Kr-h1, which acts as a DNA-binding intermediate repressor^19^. We tested the effect of dsRNA-mediated knockdown of *Kr-h1* on the H3K9-Me3 density of *DMO*, and a significantly lower histone H3K9-Me3 was recorded at seven of the eight loci analysed (Fig. 2E, Supplementary Table S3). Application of JH to iKr-h1 mosquitoes could not rescue the histone H3K9-Me3 levels to those observed in controls (iLuc+JH) (see Supplementary Fig. S9A, Table S3). These results confirm that JH induces widespread trimethylation of H3K9 histone marks via its intermediate repressor Kr-h1 for the gene *DMO* repression, but remains ineffective on Hairy-repressed *ODC* genes. Similarly, a clear induction in H3K9 trimethylation was observed in *Tep3* gene loci at 72 h PE as well as by exogenous JH; whereas, RNAi mediated knockdown of *Met* and *Kr-h1* significantly decreased H3K9 trimethylation at all tested loci (see Supplementary Fig. S7D-F, Table S6). On the contrary, there were no significant changes in H3K9-Me3 density of the *MMP* gene by JH/Met/Kr-h1 action (see Supplementary Fig. S6D-F, Table S5).

### Kr-h1 mediates JH-induced chromatin compaction of the target genes

**Fig. 3:**
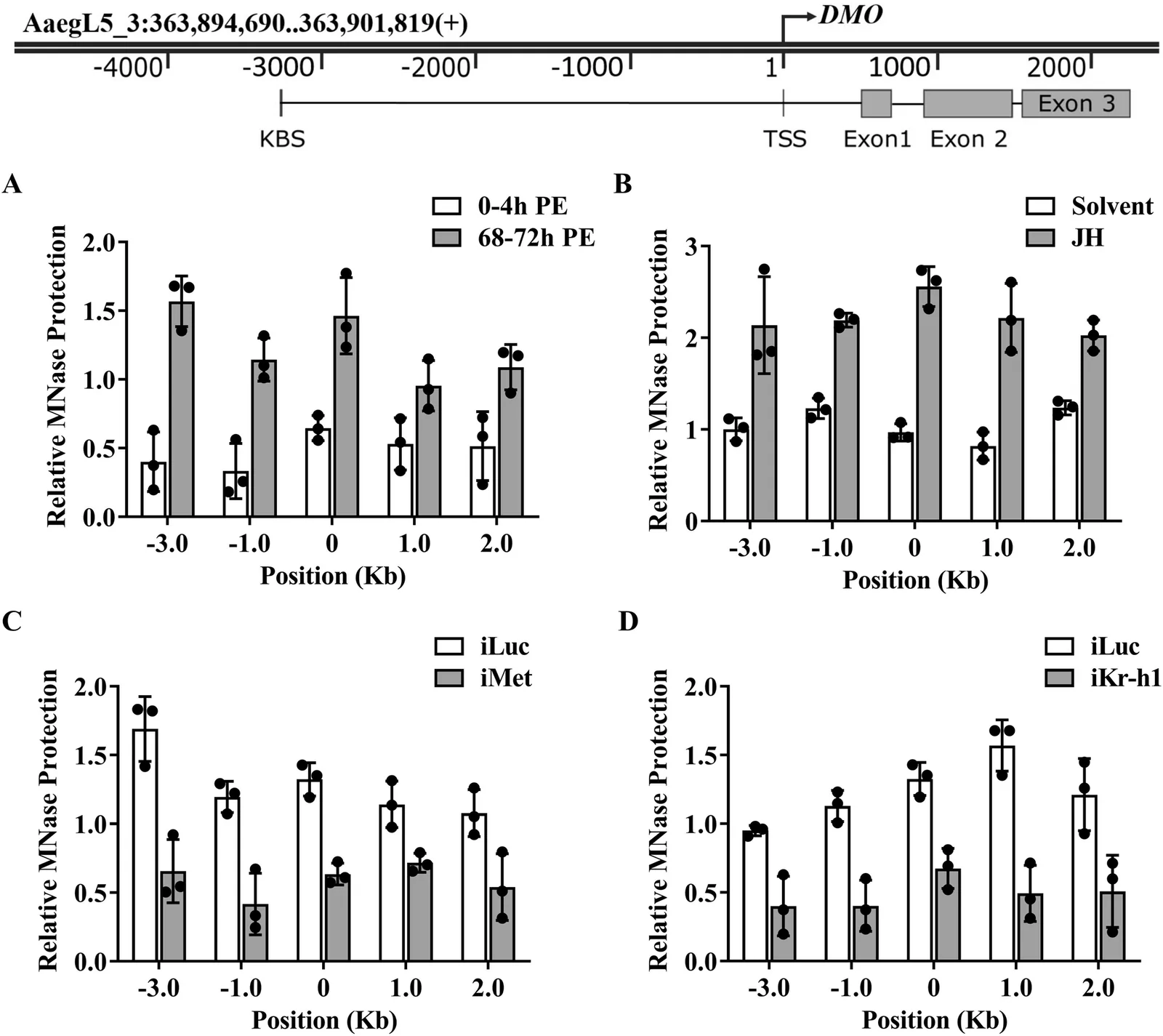
MNase protection assay demonstrating the chromatin compaction of the target gene *DMO.* **A** A distinctly lower MNase sensitivity pattern is observed in 72 h PE samples and **B** in JH-treated samples throughout all the loci. **C**, **D** Knockdown of *Met* or *Kr-h1* shows the opposite effect of ectopic JH application experiment, as significantly higher overall MNase sensitivity is observed in both iMet and iKr-h1 samples. For ectopic JH treatment, sample collection was done 6 h post treatment. For knockdown, dsRNA injection was performed on 24 h PE adult female mosquitoes followed by sample collection 48 h post injection. For all experiments, the relative MNase protection level was calculated by comparing the level of digested samples with the undigested controls. Error bars represent ± SD (n = 3). Three biological replicates were performed for all experiments with similar results. Source data are provided in Supplementary Data 1.

Histone modifications are associated with condensation of chromatin structure, making it more inaccessible to the transcriptional machinery^22,23^. To investigate the impact of the JH signaling pathway on the chromatin compaction of target genes *ODC* and *DMO*, we performed a micrococcal nuclease (MNase) protection assay in the adult female *Ae. aegypti*. Micrococcal nuclease (New England Biolabs), derived from *Staphylococcus aureus*, is an enzyme that digests DNA regions not stably bound to proteins^31^. Therefore, protection from MNase digestion indicates higher compaction of a DNA region, and the read-out is increased qPCR product levels^32^. For quantification, primer pairs targeting five loci—two in the promoter region (-1.5 kb and -0.5 kb), one harboring the TSS (0 kb), and two in the coding region (0.5 kb and 1 kb)—of the *ODC* gene were used (see Supplementary Table S1). Similarly, for the quantification of the MNase digestion profile of the *DMO* gene, primer pairs targeting five loci were used—two in the promoter region (-3 kb and -1 kb), one harboring the TSS (0 kb), and two in the coding region (1 kb and 2 kb) (see Supplementary Table S1). During PE development, MNase mapping demonstrated a clear change in chromatin compaction for *DMO* but not for *ODC*. Sensitivity to MNase digestion remained unaltered between 4 h and 72 h PE for most of the loci of *ODC* (see Supplementary Fig. S10A, Table S2). In contrast, significant resistance to MNase digestion was observed for all the loci of *DMO* at 72 h PE, but not at 4 h PE (Fig. 3A, Supplementary Table S3). Ectopic application of JH to newly eclosed mosquito abdomens resulted in greater protection from MNase digestion for all the loci of *DMO* (Fig. 3B, Supplementary Table S3). In contrast, no change in the MNase digestion profile was reported for *ODC* (see Supplementary Fig. S10B, Table S2). These results suggest that JH action induces significant compaction of *DMO* but does not impact that of *ODC*. Our knockdown experiments supported this observation that *Met* knockdown resulted in a higher MNase sensitivity of all *DMO* loci in comparison with iLuc controls (Fig. 3C, Supplementary Table S3), but did not impact the MNase digestion profile of *ODC* (see Supplementary Fig. S10C, Table S2). RNAi knockdown of *Kr-h1* demonstrated similar results to those of iMet mosquitoes, and all the *DMO* loci showed greater digestion by MNase (Fig. 3D, Supplementary Table S3). This phenotype could not be rescued by JH treatment in the iKr-h1 mosquitoes, indicating that JH-Met mediated chromatin compaction of target genes works through Kr-h1 (see Supplementary Fig. S9B, Table S3). iHairy mosquitoes, however, did not impact the MNase digestion profile of the other JH target gene, *ODC*, at most of the loci tested (see Supplementary Fig. S10D, Table S2). Using similar sets of experiments as above, the JH-mediated chromatin compaction by intermediate factor Kr-h1 was confirmed for *Tep3* gene as well (see Supplementary Fig. S7G-I, Table S6). On the contrary, no change in MNase sensitivity was observed in the case of the Hairy repressed *MMP* gene in all the different experimental frameworks (see Supplementary Fig. S6G-I, Table S5).

### Hairy and Kr-h1 induce different chromatin modifications resulting in cumulative repression of the target genes

**Fig. 4:**
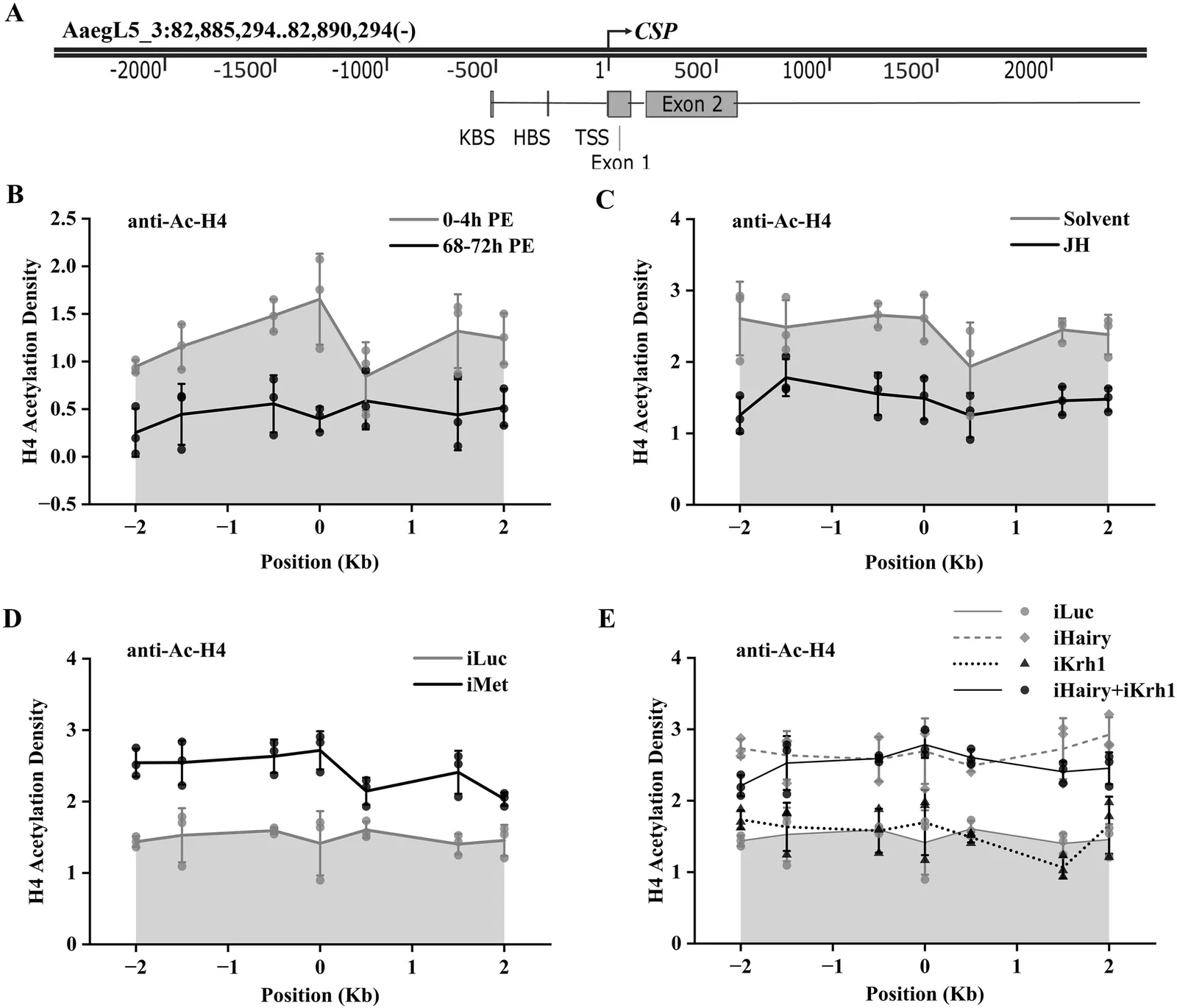
Histone H4-Ac density of target gene *CSP.* **A** Schematic representation of target region within the *CSP* gene locus along with HBS and KBS. **B** A lower level of histone H4-Ac is observed at 72 h PE in comparison with newly eclosed mosquitoes (4 h PE) at most of the gene loci assayed. **C** Similar lower global histone H4-Ac levels of the target gene are observed with 4 h PE mosquitoes that were treated with exogenous JH. **D** Knockdown of *Met* results in a compromised H4-Ac profile of the target gene. **E** Both iHairy and iHairy+iKr-h1 samples show higher H4-Ac levels across all the loci tested. However, the iKr-h1 sample shows a similar trend to that observed in iLuc control. For ectopic JH treatment, sample collection was done 6 h post treatment. For knockdown, dsRNA injection was performed on 24 h PE adult female mosquitoes followed by sample collection 48 h post injection. The H4-Ac density of target gene *CSP* was measured for all experiments to check the effect of JH and its pathway components by ChIP assay using anti-H4-Ac antibody. H4-Ac density was calculated from ChIP samples by normalizing the immunoprecipitation signal first with the H4-Ac level at the actin promoter and then with the relative H3 level. Error bars represent ± SD (n = 3). Three biological replicates were performed for all experiments with similar results. Source data are provided in Supplementary Data 1.

In our previous study, a cohort of 130 JH-Met repressed genes was reported to be regulated by both Hairy and Kr-h1 in a cooperative manner^19^. *Clip domain serine protease (AAEL005093, CSP)* is well characterised candidate gene from this cohort which harbors both HBS (-264 bp upstream of TSS) and KBS (-517 bp upstream of TSS), separated by 247 nucleotides in the promoter region (Fig. 4A). EMSA and ChIP assays have demonstrated physical binding of Hairy and Kr-h1 to these binding motifs and cell culture-based Luciferase reporter assays have demonstrated cumulative action of the two repressors for *CSP* gene^19^. The temporal expression profile of *CSP* during PE development is negatively correlated with JH titer, which is indicative of the repressive role of the hormone for the candidate gene (see Supplementary Fig. S1A). Here, we used *CSP* to investigate how Hairy and Kr-h1, working downstream of JH-Met, impact the chromatin architecture of the same target gene. As described above, the *CSP* gene was used to perform ChIP assays using histone-specific antibodies. Primers were designed against seven different loci—three in the upstream regulatory region (-2, -1, and -0.5 kb), one in the TSS region (0 kb), and three in the coding region (0.5, 1, and 2 kb)—for quantification by qPCR (see Supplementary Table S1). We observed no change in the histone H3 occupancy map of the *CSP* gene during PE development (see Supplementary Fig. S11A, Table S4). Neither the JH application nor *Met* RNAi knockdown showed any variation in the histone H3 occupancy when compared with controls (see Supplementary Fig. S11B and S11C, Table S4). Furthermore, RNAi knockdowns of *Hairy* and *Kr-h1* individually (iHairy and iKr-h1, respectively) or simultaneously (iHairy+iKr-h1) did not alter the histone H3 occupancy map of the *CSP* gene (see Supplementary Fig. S11D, Table S4). These results suggest that JH and its pathway components do not alter the histone H3 occupancy of the *CSP* gene.

We then checked for histone H4-Ac levels of *CSP* at two PE points— 4 h and 72 h —and after JH treatment. A clear drop in H4-Ac levels was observed for most of the loci tested in the *CSP* gene at 72 h PE compared with those at 4 h PE in FB (Fig. 4B, Supplementary Table S4). Similar H4 deacetylation was recorded in JH-treated samples compared to acetone-treated controls (Fig. 4C, Supplementary Table S4). *Met* RNAi knockdown had the opposite effect to the JH application, elevating H4-Ac levels throughout all assayed *CSP* loci (Fig. 4D, Supplementary Table S4). We checked the involvement of Hairy and Kr-h1 in the process by dsRNA knockdowns. Kr-h1 played no role in JH-Met-mediated deacetylation of *CSP*, as the H4-Ac profile of the gene in the iKr-h1 mosquito was similar to that of iLuc samples. However, both iHairy and iHairy+iKr-h1 samples showed elevated H4-Ac levels across all the loci tested (Fig. 4E, Supplementary Table S4). Moreover, *Hairy* knockdown compromises the JH-mediated H4 deacetylation of *CSP* as evidenced by hormonal treatment experiments of iHairy and iLuc mosquitoes (see Supplementary Fig. S12A, Table S4). These results indicate that JH-Met induces H4 deacetylation of target genes through Hairy but not Kr-h1.

**Fig. 5:**
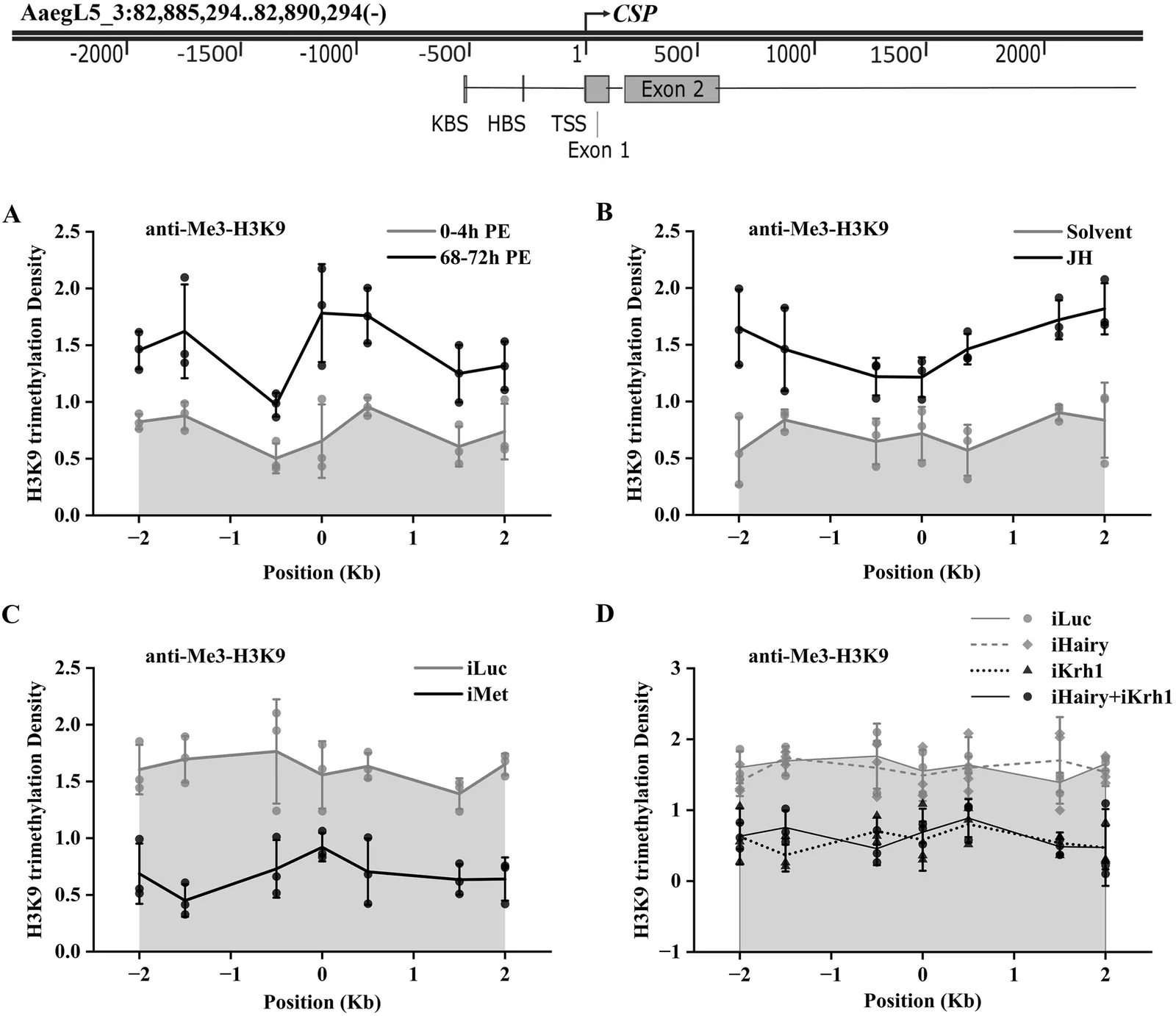
Histone H3K9-Me3 density of target gene *CSP.* **A** Higher H3K9-Me3 marks are observed at 72 h PE than in newly eclosed mosquitoes (4 h PE). **B** Similar higher overall H3K9-Me3 levels are also observed in exogenous JH-treated mosquitoes. **C** Knockdown of *Met* results in a lower H3K9-Me3 density in all assayed *CSP* loci. **D** A similar trend of lower methylation of H3K9 marks is also observed in both iKr-h1 and iHairy+iKr-h1 samples. iHairy shows no change in the overall methylation pattern of H3K9 marks at *CSP* loci. For ectopic JH treatment, sample collection was done 6 h post treatment. For knockdown, dsRNA injection was performed on 24 h PE adult female mosquitoes followed by sample collection 48 h post injection to check the effect of JH and its pathway components by ChIP assay. The H3K9-Me3 density of target gene *CSP* was measured using anti-H3K9-Me3 antibody. H3K9-Me3 density was calculated from ChIP samples by normalizing the immunoprecipitation signal first with the H3K9-Me3 level at the actin promoter and then with the relative H3 level. Error bars represent ± SD (n = 3). Three biological replicates were performed for all experiments with similar results. Source data are provided in Supplementary Data 1.

H3K9-Me3 signatures of the *CSP* gene showed a different trend. Significantly higher H3K9-Me3 marks were observed in both the late PE stage (72 h) and in JH-treated samples (Fig. 5A, B, Supplementary Table S4). *Met* RNAi knockdown showed an opposite trend, and a lower H3K9-Me3 density was observed in all assayed *CSP* loci (Fig. 5C, Supplementary Table S4). Similar significantly lower *CSP* H3K9-Me3 marks were recorded in both iKr-h1 and iHairy+iKr-h1 samples for most of the loci, a phenotype that could not be rescued by JH treatment of iKr-h1 mosquitoes, indicating the role of Kr-h1 in the process (see Supplementary Fig. S12B, Table S4). *Hairy* knockdown, however, showed a trend like that for iLuc, thus affirming that Hairy is not involved in the process (Fig. 5D, Supplementary Table S4).

**Fig. 6:**
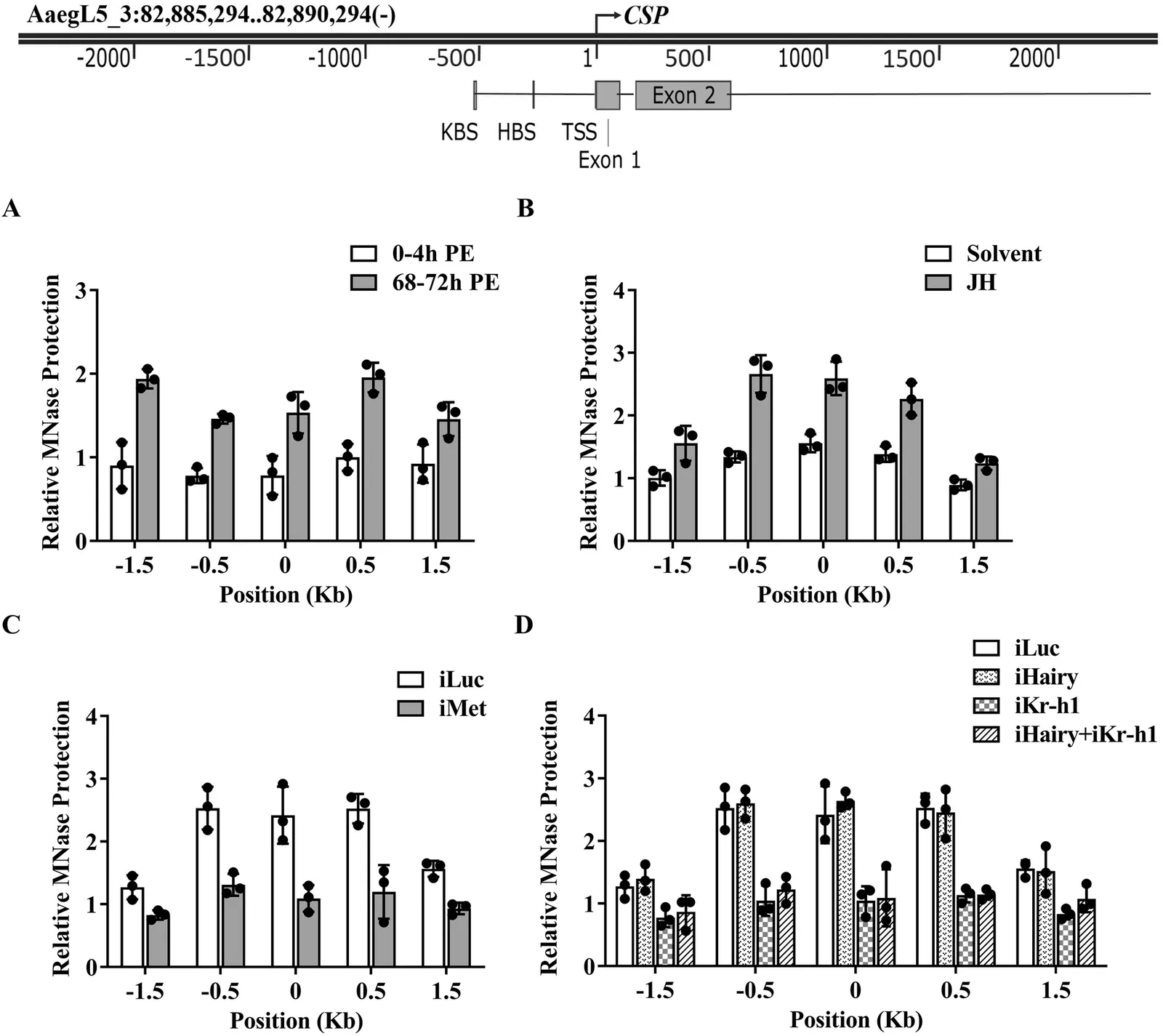
MNase protection assay of target gene *CSP.* **A** Significantly higher relative MNase protection level is observed in 72 h PE samples and **B** in JH-treated samples throughout all the loci. **C** Knockdown of Met results in significantly lower relative MNase protection in iMet samples. **D** *Hairy* knockdown did not affect the compaction level of the *CSP* gene. Still, the *Kr-h1* knockdown and double knockdown of *Hairy* and *Kr-h1* resulted in greater sensitivity of the target gene to MNase digestion. For ectopic JH treatment, sample collection was done 6 h post treatment. For knockdown, dsRNA injection was performed on 24 h PE adult female mosquitoes followed by sample collection 48 hr post injection. For all experiments, the relative MNase protection level was calculated by comparing the level of digested samples with undigested controls. All experiments were performed in triplicate with similar results. Error bars represent ± SD (n = 3). Three biological replicates were performed for all experiments with similar results. Source data are provided in Supplementary Data 1.

To understand the *CSP* gene chromatin state during the JH-controlled PE stage, we performed MNase protection assay. Primers were designed for five loci—two at the promoter, one at TSS, and two at the coding region of the *CSP* gene (Supplementary Table S1). We observed an increase in protection from MNase digestion across all loci at 72 h PE compared with that at 4 h PE, indicating the *CSP* gene condensation during PE development (Fig. 6A, Supplementary Table S4). JH treatment also enhanced protection from MNase digestion at most of the *CSP* loci tested (Fig. 6B, Supplementary Table S4). Knockdown of *Met* resulted in the opposite effect, rendering *CSP* loci sensitive to MNase digestion (Fig. 6C, Supplementary Table S4). *Hairy* dsRNA knockdown did not affect the compaction level of the *CSP* gene; the *Kr-h1* knockdown increased the sensitivity of the target gene to MNase digestion. Double knockdown of *Hairy* and *Kr-h1* showed a trend like that of iKr-h1 samples (Fig. 6D, Supplementary Table S4). Ectopic JH application in iKr-h1 mosquitoes shows no significant effect on relative MNase sensitivity of the *CSP* gene (see Supplementary Fig. S12C, Table S4). However, clear induction in relative MNase protection was observed after JH treatment in iLuc mosquitoes (see Supplementary Fig. S12C, Table S4). Taken together, we conclude that Kr-h1 mediates JH-Met-induced compaction of the *CSP* gene, but not Hairy.

We performed the same experiments on the second candidate gene *Glutamine gamma-glutamyltransferase* (*GGG)*, regulated cumulatively by intermediate factors Hairy and Kr-h1, downstream of JH-Met. For quantification, primer pairs targeting four loci—2 in the promoter, one harboring the TSS and one in the coding region of the *GGG* gene, were used (see Supplementary Table S1). A clear reduction in H4 acetylation marks was observed at 72 h PE and after JH treatment throughout all the tested loci of the *GGG* gene (see Supplementary Fig. S13A and S13B, Table S7). A significant increase in H4 acetylation was observed in iMet, iHairy, and iHairy+iKr-h1 samples but not in iKr-h1 samples (see Supplementary Fig. S13C, Table S7). Likewise, JH treatment increased H3K9 trimethylation of the *GGG* gene (see Supplementary Fig. S13D-F, Table S7). However, this induction of H3K9 trimethylation was controlled by Met and Kr-h1, not by Hairy (see Supplementary Fig. S13F, Table S7). As demonstrated by the MNase protection assay, the JH-induced chromatin compaction of the *GGG* gene was also controlled by Kr-h1, with the knockdown of *Hairy* having no effect on the MNase sensitivity of the candidate gene (see Supplementary Fig. S13G-I, Table S7).

## Discussion

Recently, several studies have investigated chromatin changes in the context of JH and its signaling pathway. CREB binding protein (CBP) is a HAT that has been demonstrated to play a role in JH-mediated induction of *Kr-h1* in the cockroach *Blattella germanica*^33^. In addition to Kr-h1, CBP was also linked with the induction of other JH-regulated genes in *Tribolium*^34,35^. CBP was found to act via acetylation of core histone proteins, specifically H3K18 and H3K27 in the JH context^34,35^. The chromatin modifier HDACs have also been implicated in playing a role in JH action^34–36^. HDAC1, in association with SIN3, acts via deacetylating the histones, particularly H2BK5 residues of the target gene in *Tribolium*^36^. JH/Met represses the mRNA levels of HDAC1, therefore leading to increased acetylation of histone H2BK5 residues. In *Tribolium*, JH-mediated CBP recruitment and HDAC repression jointly contribute to the acetylation of several histone residues on target genes, leading to their expression^34–36^. These two factors, CBP and HDAC, have been reported to be involved in JH signaling in *Ae. aegypti* mosquito larvae^37,38^. JH and CBP action, via increased histone acetylation, facilitates the recruitment of JH receptor and other proteins tothe promoter of target gene *Kr-h1*^37^. However, JH and Met induce HDAC3, which along with the co-repressor SMRTER, suppresses pro-apoptotic genes that play a role in larval midgut remodeling^38^.

In this study, we focused on chromatin remodeling initiated by two specific components of the JH signaling pathway—Kr-h1 and Hairy. We found that Hairy action leads to widespread deacetylation of histone H4 throughout the target gene loci, without inducing significant chromatin compaction or change in the histone H3 occupancy map. Various reports have demonstrated that Hairy, through its C-terminal WRPW domain, interacts with the co-repressor Groucho in different organisms, including mosquitoes^17,27,39–41^. Groucho is known to recruit class-I HDAC Rpd3, a component of a co-repressor complex that contains other chromatin-binding proteins such as Sin3 and RbAp48^27,28,42^. These complex deacetylates the lysine residues of all four core histones of the target genes, leading to their repression^23,43^. A similar mechanism might be active in the case of the JH signaling pathway involving Hairy in mosquitoes. A long-range target gene repression might be achieved by a Groucho-mediated “spreading” mechanism, where the co-repressor is recruited several kilobases downstream of Hairy binding site including the transcribed region of the target gene^27,30,44–47^. In contrast to Hairy, Kr-h1 action leads to H3K9 trimethylation and chromatin compaction, as demonstrated by MNase protection assay. We did not observe any change in the acetylation levels of histone H4 after Kr-h1 action. The mammalian homologs of Kr-h1, Krüppel like factors (KLFs), are known to recruit co-repressors that can function as chromatin remodelers^26,29^. Previously, C-terminal binding proteins (CtBPs) and Sin3-HDAC systems have been linked to KLF function^29,48^. Later studies have revealed that some KLFs, carrying specific domains, can recruit heterochromatin protein 1 (HP1) and its associated HMT protein, SUV39H1^26,49^. This HP1-HMT complex is known to methylate adjacent histone H3K9 residues. Interestingly, HP1 is recruited to these H3K9-Me3 marks leading to long-distance spreading of repressive methylation throughout the chromatin^26,29,50,51^. In *Drosophila*, SUV39H1 homolog SU(VAR)3-9 has also been demonstrated to play a role in HP1-mediated H3K9 methylation and repression of euchromatic genes^51,52^. Additionally, another class of protein belonging to the C2H2 Zinc finger family- Krüppel associated box domain-zinc finger protein (KRAB-ZFPs) has been demonstrated to induce H3K9 trimethylation via KRAB associated protein 1 (KAP1), resulting in chromatin compaction several kilobases away from transcription factor binding site^53^. This process is known to involve the histone methyltransferase SETDB1 and HP1^50,53^. It is likely that in mosquitoes, Kr-h1 mediated repression involves components of the pathways discussed above, including HP1, for the observed long-range effect.

JH/Met-regulated transcriptional repressors Hairy and Kr-h1 work cumulatively for a subset of genes^19^. Our results demonstrate that for co-regulated genes, Hairy action results in deacetylation of histone H4, while Kr-h1 action leads to an increase in histone H3K9-Me3 marks and chromatin compaction of the target gene. Although induced independently by the two factors, the chromatin modifications will contribute to the expression status of the target gene and may hold the clue to the mechanistic understanding of the cumulative action of Hairy and Kr-h1 downstream of JH-Met. It should be noted that cooperative or synergistic action between chromatin-modifying factors has been reported before in yeast and human cell lines. In yeast, transcription factors Mot3 and Rox1 work synergistically, leading to gene repression^54^. Additionally, in yeast, ATP-dependent chromatin remodeling factor Isw2 complex and Sin3-Rpd3 HDAC complex show synergistic action in Ume6p-mediated gene repression^55^. Similarly, ATP-dependent chromatin remodeling and histone acetylation function cooperatively in regulating estrogenic receptor signaling in human cell lines^56^. In *Drosophila*, nucleosome remodeling factor (NURF) and Imitation Switch (ISWI) have been reported to function synergistically in the activation of genes^57^. These reports further support the possibility that Hairy and Kr-h1-induced alternative chromatin modifications have a cumulative effect on the repression of target genes.

## Conclusions

The two intermediate repressors of the JH-Met pathway—Hairy and Kr-h1—induce alternative chromatin modification to repress their respective target genes. While JH promotes histone H4 deacetylation via Hairy for repression, it induces histone H3K9 trimethylation and chromatin compaction via another repressor, Kr-h1, during their respective target gene repression. However, JH doesn’t induce any change in histone H3 occupancy map while repressing its target genes. In the case of genes co-regulated by both factors, Hairy induces histone H4 deacetylation, while Kr-h1’s action results in histone H3K9 trimethylation and chromatin compaction, leading to cumulative repression of the target gene. These findings may hold the key to understanding the mechanism behind cumulative repression of target genes by Hairy and Kr-h1 in the Dengue vector mosquito, *Ae. aegypti*.

## Methods

### Ethics statement

This study has approval from Institute Biosafety committee of Birla Institute of Technology and Sciences- Pilani, K K Birla Goa Campus (BPGC/2019/IBSC-3) for use of nonblood-fed mosquitoes, which does not involve any GMO with specialized biosafety precautions. National Institute of Malaria Research, Field Unit, Goa, has the approval of institutional ethics committee of NIMR, ICMR, New Delhi (NIMR/IAEC/2022-1/10) for rearing and maintenance of cyclic mosquito lines in the insectary and use of chicken for blood feeding.

### Experimental animal

*Ae. aegypti* mosquitoes were maintained in the insectary of NIMR, field unit, Goa. In this facility, the larvae were reared in plastic trays containing tap water at 28°C temperature and a humidity of around 85%, and a photoperiod: scotoperiod of 12:12 h (light: dark cycles). The larvae were fed on fish food till their metamorphosis into pupae. The adults were supplied with 10% (w/v) glucose soaked in cotton pads and fed with chicken blood for reproduction. For experiments, adult *Ae. aegypti* was sourced from the insectary of NIMR, field unit, Goa to the insectary at BITS-Pilani, Goa Campus. Mosquitoes were maintained at 28°C temperature and a humidity of 85% in the insect growth chamber and supplied with 10% (w/v) glucose solution as a food source.

### In vivo hormonal treatment

The newly emerged female mosquitoes (4 h PE) were anesthetized under cold shock and treated topically on the dorsal abdomen with 0.3 µl of 1 µg/ml JH-III (Sigma) dissolved in acetone or the vehicle alone as a control. FBs were collected 4-6 h post-treatment.

### Cloning

A 1.4 kb promoter region upstream 3 kb from TSS of the JH/Met/Kr-h1 target gene *DMO*, (*AAEL000834*) harboring potential KBS was PCR amplified and cloned into reporter vector PGL3basic (Promega), followed by sequencing for confirmation. Desired clones were used for transient transfection into cultured *Drosophila* S2 cells, using the FuGENE HD reagent (Promega) as per manufacturer’s protocol.

### dsRNA-mediated gene silencing

Knockdown of target genes was achieved by injecting gene-specific dsRNA into the thorax of the cold-anesthetized mosquito at 24 h PE using an Eppendorf Femtojet 4X microinjection system. dsRNA synthesized from the bacterial *luciferase* gene (iLuc) was used as a control. To prepare the template for dsRNA synthesis, total RNA was extracted from adult female *Ae. aegypti* FB using trizol based method and cDNA was synthesized using RevertAid First Strand cDNA Synthesis Kit (Thermo Scientific) following manufacture’s protocol. PCR was performed with cDNA using Qiagen PCR mastermix to prepare the template of dsRNA against specific genes. dsRNA was synthesized using the MEGAscriptTM T7 Transcription kit (Thermo Scientific) following the manufacturer’s protocol. Before injection, synthesized dsRNAs were pre-cleaned using the phenol-chloroform extraction method and resuspended in nuclease-free water at a final concentration of 4 μg/μl. Of the respective dsRNA, 0.3 μl was injected into the thorax of each female to achieve knockdown of the target gene. To confirm the efficiency of knockdown, 5 FBs were collected 48 h post injection for total RNA extraction followed by cDNA synthesis. Expression of *Met*, *Hairy* and *Kr-h1* was measured by qPCR to validate the knockdown of respective genes. Results were incorporated in the Supplementary Fig. S14. Knockdown validated samples were further used for all downstream experiments. Primers used for PCR were prepared using the transcript sequences obtained from Vectorbase, and their details are listed in Supplementary Table S1.

### Chromatin immunoprecipitation assay

ChIP assay was performed using FB tissue samples as described previously^30^ with few modifications. In brief, mosquito FB tissues were homogenized in PBS on ice. Homogenized tissues were initially crosslinked for chromatin extraction by incubating in 3% formaldehyde fixation buffer, followed by nuclear extraction. Chromatin samples were sonicated for 30 s (100% duty cycle) for 12 repetitions with a 1-min cooling interval using a bath sonicator to obtain a chromatin DNA of 200–300 bp. For immunoprecipitation, three technical replicates of each sample were used, and 50 μl of each sample were kept aside as input samples. Chromatin samples were immunoprecipitated using histone-specific antibodies: in this study, rabbit anti-H3 (Abcam), rabbit anti-acetyl H4 (Upstate; polyclonal antibody which can target multiple lysine residues - Lys5, Lys8, Lys 12, Lys16), and rabbit anti-trimethyl H3K9 (Abcam) antibodies. IP samples were incubated overnight at 65°C to induce reverse crosslinking, and the DNA fragments were eluted and purified using the phenol-chloroform extraction method. IP samples were then analyzed utilizing qPCR using position-specific primers for generating histone H3 occupancy, histone acetylation, and methylation map.

For ChIP assays from *Drosophila* S2 cells, the protocol described in Saha et al. 2019 was followed^19^. Briefly, 5–10×10^6^ transfected cells for each sample were fixed with 1% formaldehyde for crosslinking, followed by chromatin extraction using the same protocol as described for the tissue samples. Previously generated *Ae. aegypti* anti-Kr-h1 and anti IgG (control) antibodies were used for immunoprecipitation.

### Micrococcal nuclease protection assay

MNase protection assay was performed using mosquito FB tissues as described previously in Roy et al.,^32^. In brief, FBs dissected from female mosquitoes were homogenized and incubated with 2% formaldehyde for cross-linking. The reaction was stopped using 1 M glycine, and then nuclei and chromatin preparation was carried out as described by Petesch et al.,^58^. The chromatin lysate was centrifuged, and the pellet was washed and resuspended in the MNase digestion buffer. Half of the extract was stored as undigested control, and the remaining extract was treated with 100 U MNase enzyme (NEB) for 30 min. The reaction was quenched by adding 12.5 mM EDTA and 0.5% SDS. Both the chromatin samples were centrifuged at 14,000 rpm for 10 min at 4 °C and reverse-crosslinked by incubating at 65 °C overnight. The DNA samples were extracted using phenol-chloroform and subjected to qPCR-based enrichment analysis.

### Quantitative PCR analysis

qPCR was performed with SyBr Green (Brilliant III Ultra-Fast SYBR Green QPCR Master Mix, Agilent) based quantification for both ChIP and MNase assay samples using the AriaMx Agilent qPCR system. For ChIP samples, histone H3 occupancy was calculated by normalizing the immunoprecipitation signal with the histone H3 occupancy level at the actin promoter. H4-Ac or H3K9-Me3 density was calculated from ChIP samples by normalizing the immunoprecipitation signal first with the H4-Ac or H3K9-Me3 level at the actin promoter, respectively, and then with the relative H3 level. Using the MNase protection assay, a ratio was calculated between MNase digested and undigested samples. The primers used for both ChIP and MNase assay are listed in the Supplementary Table S1.

### Statistics and reproducibility

Statistical significance for all the data generated from ChIP and MNase assays were calculated from three biological replicates (n = 3) with similar results using unpaired two-tailed parametric t-test. Each sample was prepared from fifty mosquito FB for ChIP and MNase assay. Threshold for the *p*-values were set as p < 0.05 for significance. All the *p*-values for ChIP and MNase data are listed in the supplementary table (see Supplementary Table S2-S7). For gene expression study, three biological replicates (n = 3) with six mosquito FB per sample were used. Statistical significance was calculated using unpaired two-tailed parametric t-test. The *p*-values were represented in graphs as *p < 0.05; **p < 0.005; ***p < 0.0005; ****p < 0.00005 and ‘ns’ for not significant.

### Reporting summary

Further information on research design is available in the Nature Portfolio Reporting Summary linked to this article.

## Supplementary information

Transparent Peer Review file

Supplementary Information

Description of Additional Supplementary Files

Supplementary Data 1

Supplementary Data 2

Supplementary Data 3

Reporting Summary

## Data Availability

Source data for all graphs and figures of the paper can be found in the Supplementary Data 1. dsRNA sequences used for knockdown of specific genes can be found in Supplementary Data 2. Sequence of plasmid generated during the course of the investigation has been provided as a references sequence file in “Supplementary Data 3”. All other data are available from the corresponding author (or other sources, as applicable) on reasonable request.
