# Supplementary material for: Juvenile hormone-mediated chromatin remodelling by cumulative action of downstream transcription factors, Hairy and Krüppel homolog 1
Source: Commun Biol. 2026 May 26;9:1214. doi: 10.1038/s42003-026-10327-4 (PMC13586389; doi:10.1038/s42003-026-10327-4)
Supplement: Supplementary file 1 [file 42003_2026_10327_MOESM1_ESM.pdf]

nature portfolio

Peer Review File

Juvenile hormone-mediated chromatin remodelling by cumulative action of downstream transcription factors, Hairy and Krüppel homolog 1

Corresponding Author: Dr Tusar Saha

This file contains all reviewer reports in order by version, followed by all author rebuttals in order by version.

Version 0:

Reviewer comments:

Reviewer #1

(Remarks to the Author)
This manuscript investigates the epigenetic mechanisms underlying juvenile hormone (JH)-mediated transcriptional repression during mosquito reproduction. JH plays a central role in stage-specific gene regulation through transcriptional repressors such as Kr-h1 and Hairy. While some target genes are regulated by either Kr-h1 or Hairy independently, others require both factors for repression. However, the chromatin-level mechanisms by which these repressors act remain poorly understood.

Banerjee et al. use Aedes aegypti as a model to examine the distinct chromatin remodeling events associated with Kr-h1 and Hairy binding. The authors demonstrate differential recruitment of corepressors and link this to specific changes in histone modifications and gene expression. The work provides valuable insights into the synergistic repression by these JH-induced repressors and advances our understanding of JH-regulated gene networks in both mosquito reproduction and insect metamorphosis.

The experimental design is generally sound and includes appropriate controls. The manuscript is clearly written; however, several issues should be addressed to strengthen the conclusions:

Specific comments:
1. The study investigates histone modifications and DNA accessibility in conjunction with Kr-h1 and Hairy binding and their target gene expression. However, it is unclear whether Kr-h1 or Hairy is bound to these target genes at the early 0–4 h time point. Additionally, the manuscript does not show whether expression levels of these targets are reduced at 68–72 h compared to 0–4 h. These data are essential to support the proposed links between transcription factor binding, histone modifications, and transcriptional repression.
2. The authors should assess whether RNAi-mediated knockdown of Met, Kr-h1 or Hairy alters the chromosomal binding of Kr-h1 and Hairy and whether these changes affect the expression of target genes. These analyses would help clarify the specificity and hierarchy of the regulatory mechanisms.
3. The figure legends indicate that all experiments were performed in triplicate with consistent outcomes. However, it is unclear whether these represent biological replicates from independent experiments. Please clarify the nature of the replicates used.
4. Histone H4 acetylation is typically enriched at promoters and enhancers, yet Fig. 1B shows no significant enrichment at either time point. The authors should discuss potential technical or biological reasons for this unexpected result.
5. Several experiments involve RNAi or topical JH treatment. The timing of sample collection following each treatment should be clearly stated in the figure legends to aid interpretation.
6. Line 52: The description of the Met–Hsp83 interaction should be revised for accuracy. Current evidence indicates that Met is associated with Hsp83 in the absence of JH, and this complex dissociates upon JH binding.

Reviewer #2

(Remarks to the Author)
The manuscript provides significant advances in understanding juvenile hormone (JH) signaling by elucidating how downstream repressors (Hairy and Kr-h1) drive chromatin-level repression in mosquitoes. Combines multiple approaches (ChIP-qPCR, MNase assays, RNAi, hormonal treatments) across developmental time points, the authors showed that Hairy and Kr-h1 induced alternative epigenetic modifications (H4 deacetylation vs. H3K9me3/chromatin compaction). While the specific combination of H4 deacetylation and H3K9me3 induced by JH repressors in mosquitoes have not been previously reported, the core epigenetic mechanisms themselves (histone deacetylation and methylation as repressive marks) represent well-established, general paradigms for transcriptional repression by transcription factors. The study largely documents the occurrence of these common modifications rather than providing novel mechanistic insights into how Hairy and Kr-h1 specifically recruit or interact with the chromatin-modifying complexes (e.g., HDACs, HMTs) responsible. This lack of deeper mechanistic exploration into the recruitment and function of these modifiers limits the conceptual advancement. In addition, several key conclusions appear to extend beyond what the presented experimental evidence can robustly support.

Major Comments：
1.The claim of "synergistic action" between Hairy and Kr-h1 is not convincingly demonstrated. The data show additive or cumulative effects on repression for co-regulated genes (H4 deacetylation plus H3K9me3/compaction), but provide no evidence of biochemical cooperation or mutual enhancement between the factors themselves in inducing each other's modifications or binding. True synergy requires evidence that the combined effect exceeds the sum of individual effects, which is lacking here. The term "synergistic" should be replaced with "additive" or "cumulative".
2.The assertion that JH-Met induces modifications "through" Hairy or Kr-h1) implies a direct and necessary role. However, the critical rescue experiments (iHairy + JH; iKr-h1 + JH) to demonstrate that the absence of the factor abolishes the JH-induced modification are missing. The current knockdown data (iHairy or iKr-h1 alone) only show correlation, not causation. Without these controls, the conclusions appear somewhat far-fetched.
3.The manuscript focuses on histone H3 occupancy as a proxy for overall histone position. Please clarify why histone H3, rather than H2A, H2B, H4, or a core histone pan-antibody, was specifically chosen for these experiments. Is there evidence suggesting H3 occupancy is the most representative or stable marker for general nucleosome positioning in this system?
4."histone position" in line 139 should be explicitly defined as referring to "histone H3 occupancy".
5.Figure 1C/D/E and Figure 2C/D/E should indicate the time points of detection, whether it is at 72 h PE. If so, the apparent ~2-fold decrease in H4-Ac levels observed in the solvent (Fig.1C) or iLuc controls (Fig 1D/E) compared to the 72h PE time point in Fig 1B is concerning. A similar potential discrepancy seems present in the H3K9me3 data (Fig 2B vs Fig 2C/D/E). This raises questions about experimental consistency or normalization across different experimental sets (developmental time course vs. hormone treatment vs. knockdowns). Please address this potential inconsistency and ensure robust normalization strategies are applied uniformly across all datasets.
6.Hairy binds ~1 kb upstream of the ODC TSS, and Kr-h1 binds ~3 kb upstream of the DMO TSS. It is therefore striking that JH, Met, or Hairy, Kr-h1 knockdowns induce changes in H4-Ac or H3K9me3 levels across all 8 assayed loci spanning promoter and coding regions. The manuscript should discuss potential mechanisms for this long-range effect. Does it involve chromatin spreading, looping, or recruitment of modifiers capable of acting over large distances? This is a significant finding that merits deeper discussion beyond just stating the observation.
7.Several procedural details currently in the Results section (e.g., antibody sources, statistical method) are more appropriate for the Methods section. The Results should focus on presenting and interpreting the findings, while ensuring all necessary methodological information is accurately and concisely detailed in the dedicated Methods section.
8.Language Clarity and Flow:
a)Line 62: Ambiguous pronoun: "Unlike Met-mediated gene activation, its repression is indirect..." Change to: "Unlike Met-mediated gene activation, repression mediated by Met is indirect, requiring the involvement of additional intermediate factors."
b)Line 119: Inaccurate contrast: "In contrast, DMO has a Kr-h1 binding site (KBS)..." Replace with a neutral connector: "Separately, DMO has a Kr-h1 binding site (KBS)..." or "For DMO, a Kr-h1 binding site (KBS) was located..."
c)Line 209: "Similarly, a clear induction in H3K9 trimethylation was observed in Tep3 gene loci by JH and Kr-h1..." Rewrite this sentence to describe the Kr-h1 RNAi results.

Reviewer #3

(Remarks to the Author)
Authors did not show the evidence of the binding of Hairy and kr-h1 on the binding sites. Authors did not show the evidence of recruitment of cofactors or histone. Authors need to explain the results of chip analysis, therefore it is not easy to understand so many acetyl- H4 or H3K9me3 around the target gene locus. The trial is important but the methods and the explanations are not enough.

Version 1:

Reviewer comments:

Reviewer #1

(Remarks to the Author)
The revised manuscript has addressed many of the concerns raised in the previous reviews and shows significant improvement.

However, the authors did not verify the knockdown efficiency in their RNAi experiments. Ideally, both mRNA and protein

levels of Kr-h1 and Hairy should be assessed. Instead, the authors rely on data from their earlier publications to suggest successful RNAi, but those results are not from the current experiment and therefore can't be considered applicable here.

Reviewer #2

(Remarks to the Author)
The authors have adequately addressed all my concerns. I support acceptance.

Open Access This Peer Review File is licensed under a Creative Commons Attribution 4.0 International License, which permits use, sharing, adaptation, distribution and reproduction in any medium or format, as long as you give appropriate credit to the original author(s) and the source, provide a link to the Creative Commons license, and indicate if changes were made.
In cases where reviewers are anonymous, credit should be given to 'Anonymous Referee' and the source.
The images or other third party material in this Peer Review File are included in the article's Creative Commons license, unless indicated otherwise in a credit line to the material. If material is not included in the article's Creative Commons license and your intended use is not permitted by statutory regulation or exceeds the permitted use, you will need to obtain permission directly from the copyright holder.
To view a copy of this license, visit https://creativecommons.org/licenses/by/4.0/

Point-by-point response to the reviewers

Editor's Comments:

We hope you will find the referees' comments useful as you decide how to proceed. Should further experimental data or analysis allow you to address these criticisms, we would be happy to look at a substantially revised manuscript. In particular, we would need demonstration that 1) Kr-h1 and Hairy bind to the assessed binding sites, 2) that the absence of Hairy or Kr-h1 abolishes the JH-induced modifications reported, and 3) that Kr-h1 and Hairy recruit cofactors or histones to induce the chromatin modifications reported. Further, address the concerns of R2 regarding use of the term 'synergy' in the manuscript.

We sincerely thank you for considering our manuscript and for your valuable comments. Our responses are as follows-

1. Hairy binding to the specific DNA motifs in the promoter of *ODC* gene has been demonstrated in great details in our previous manuscript (Saha et al., 2016). The DNA-protein interaction has been confirmed through high quality EMSA studies with cold probe based competition assays and mutation analysis (Saha et al.; 2016). The results were further confirmed by clear super-shifts using *Aedes* Hairy antibody (Saha et al., 2016). Similarly, Kr-h1 binding to specific promoter binding site of *CSP* gene was demonstrated through EMSA assays as well (Saha et al., 2019). Additionally, ChIP assays with *Aedes* Kr-h1 antibody was performed to confirm this DNA protein interaction. *CSP* is a gene co-regulated by Hairy and Kr-h1 and Hairy binding to specific response elements in the *CSP* promoter was validated via ChIP assays (Saha et al., 2019).
   For *DMO*, a gene regulated by Kr-h1, the Kr-h1 binding site were identified computationally (Saha et al, 2019). However, evidence of physical interaction between Kr-h1 and its binding elements from *DMO* promoter was missing. We have performed ChIP assays using *Aedes* Kr-h1 antibody in *Drosophila* S2 cell culture system. Cells were transfected with plasmids overexpressing Kr-h1 (Kr-h1-Myc) and plasmids harboring 1.4 Kb promoter region of DMO gene with the putative Kr-h1 binding site (KBS). A clear enrichment of IP signals was observed with primer pairs targeting the KBS region indicating binding of Kr-h1 to KBS. Primer pairs targeting control regions from the backbone of the plasmid did not show any signal. The details of this experiment are given in line no 127 to 132 of result section and Line 451 to 456 of methods section along with the graph in the supplementary figure 1B of supplementary file 1.
2. Certainly, this is a critical evidence that we missed providing earlier. However, the suggested rescue experiments have been performed and provided with this revised version. iHairy and iKr-h1 mosquitoes were treated with JH (or control acetone) 48hrs post injection. Samples were collected 6hr post treatment followed by ChIP or MNase analysis as required. JH failed to induce the chromatin changes observed in *ODC*, *DMO* and *CSP* genes in the absence of the corresponding factor/s. The details have been provided in the revised manuscript lines 185-188, 226-227, 264-266, 308-310, 316-319, and 332-335. The corresponding figures are incorporated as Supplementary file, Fig. S5, S9 and S12. These set of experiments

unambiguously establish that JH/Met induces the target gene chromatin changes via Hairy or Kr-h1.

3. We acknowledge the concerns raised by the reviewers and editors regarding the lack of evidence pertaining to recruitment and interaction with chromatin modifying complex like HMTs and HDACs. Indeed, providing these evidences would make the entire story complete. We have been working on this for some time now and we plan to publish these results as a separate subsequent manuscript. We thought that the topic of associated chromatin modifying complexes deserve separate attention because of the complexity brought about by the combinatorial action of Hairy and Kr-h1, and the involvement of multiple co-factors for both. Furthermore, adding the entire data would make the current manuscript quite long and complex. Thus we decided to report the target gene chromatin changes associated with JH and its signalling pathway components in the first paper, and the details of the recruitment of chromatin modifying complexes in a subsequent paper. I would hope that the reviewers find merit in our argument and kindly agree to let us follow through our plan.
4. We agree with the reviewer 2 that the observed effect is more cumulative/additive in nature rather than synergistic. The reason for this is historic, as we labelled the phenomenon as synergistic effect, albeit due to oversight, in our 2019 paper (Saha et al.; 2019). Taking reviewers suggestion, the term "synergistic" has been replaced with either "cumulative" or "additive" throughout the manuscript in the revised version. Accordingly, the Title of the manuscript has been altered as well.

Reviewers' comments:

Reviewer #1 (Remarks to the Author):

This manuscript investigates the epigenetic mechanisms underlying juvenile hormone (JH)-mediated transcriptional repression during mosquito reproduction. JH plays a central role in stage-specific gene regulation through transcriptional repressors such as Kr-h1 and Hairy. While some target genes are regulated by either Kr-h1 or Hairy independently, others require both factors for repression. However, the chromatin-level mechanisms by which these repressors act remain poorly understood.

Banerjee et al. use Aedes aegypti as a model to examine the distinct chromatin remodeling events associated with Kr-h1 and Hairy binding. The authors demonstrate differential recruitment of corepressors and link this to specific changes in histone modifications and gene expression. The work provides valuable insights into the synergistic repression by these JH-induced repressors and advances our understanding of JH-regulated gene networks in both mosquito reproduction and insect metamorphosis.

The experimental design is generally sound and includes appropriate controls. The manuscript is clearly written; however, several issues should be addressed to strengthen the conclusions:

Thank you for your time and encouraging comments. We have addresses your concerns point by point as below.

Specific comments:

1. The study investigates histone modifications and DNA accessibility in conjunction with Kr-h1 and Hairy binding and their target gene expression. However, it is unclear whether Kr-h1 or Hairy is bound to these target genes at the early 0–4 h time point. Additionally, the manuscript does not show whether expression levels of these targets are reduced at 68–72 h compared to 0–4 h. These data are essential to support the proposed links between transcription factor binding, histone modifications, and transcriptional repression.

JH bound Met directly binds to the promoter of Hairy and Kr-h1 leading to their activation. Previously, JH titer in adult female *Aedes aegypti* has been determined. Additionally, expression of both Hairy and Kr-h1 during post eclosion (PE) period been well characterized via qRT-PCR and Western Blot analysis (Saha et al., 2016 and Ojani et al., 2018). Published results of the Western blot based protein estimation are provided for your reference. Clearly no Hairy or Kr-h1 protein is expressed at 0-4h time frame (A time point when JH titer is basal). In the absence of the protein, the possibility of Hairy and Kr-h1 binding to the chromatin does not arise.

Thank you for pointing out that the manuscript is missing time course expression data for target genes. Indeed, that was an oversight. We have now added temporal expression data for target genes *ODC, DMO* and *CSP* during PE development in adult female *Ae. aegypti.* All three target genes show a high expression right after adult eclosion (0-4h and 12-16h PE), with intermediate expression levels observed around 24-28h PE and gradually dropping to low expression during late PE (36-40h, 48-52h and 68-72h). The results have been discussed in the main manuscript (Line 120-125 and 282-284) and the corresponding figures are incorporated as Supplementary file, Fig. S1A).

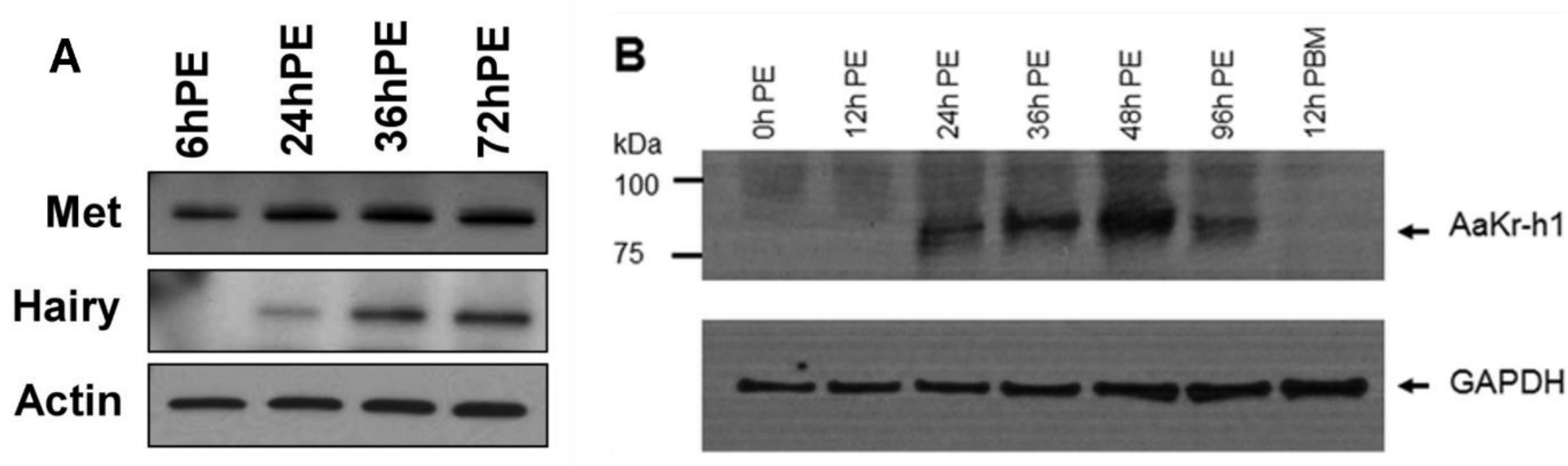

Fig. A) Temporal protein expression of *Ae. aegypti* Met and Hairy during PE development (From Saha et al.; 2016) B) Temporal protein expression of *Aa*Kr-h1 in adult female during PE. (From Ojani et al.; 2019).

2. The authors should assess whether RNAi-mediated knockdown of Met, Kr-h1 or Hairy alters the chromosomal binding of Kr-h1 and Hairy and whether these changes affect the expression

of target genes. These analyses would help clarify the specificity and hierarchy of the regulatory mechanisms.

dsRNA mediated knockdown of Met, Hairy and Kr-h1 via thoracic injections is a well-established and highly efficient technique. Following dsRNA injection the protein levels of the three factors are reduced to almost nil, as demonstrated in this published figure from our previous study (Saha et al.; 2019). In the absence of the proteins the question of chromosomal binding of these proteins in respective iMet, iHairy and iKr-h1 samples does not arise. Therefore, knockdowns will certainly alter the chromosomal binding of the respective factors and affect the expression of target genes in comparison to the controls.

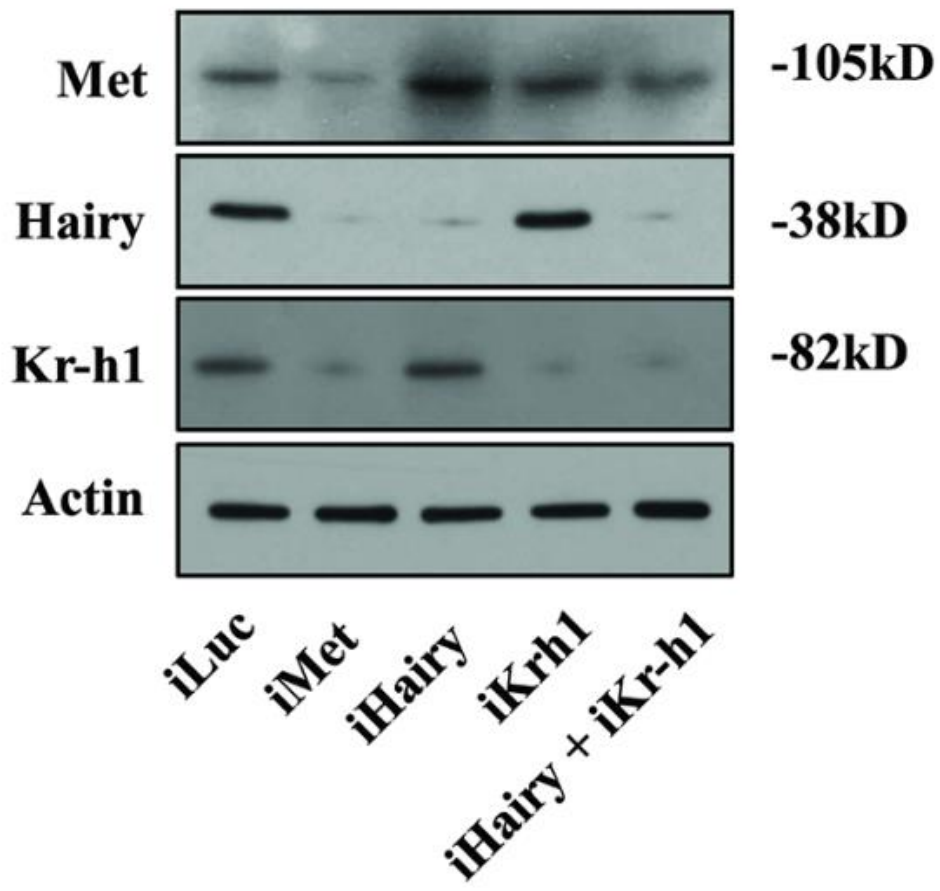

Fig. Protein expression of Met, Hairy and Kr-h1 in iMet, iHairy, iKr-h1 and iHairy+iKrh1 samples in adult female *Ae. aegypti* by western blot analysis. (From Saha et al.; 2019)

3. The figure legends indicate that all experiments were performed in triplicate with consistent outcomes. However, it is unclear whether these represent biological replicates from independent experiments. Please clarify the nature of the replicates used.

Yes, for all experiments we had three biological replicates derived from independent batches of mosquito which has been maintained in separate trays and cages. We apologise for the confusion.

4. Histone H4 acetylation is typically enriched at promoters and enhancers, yet Fig. 1B shows no significant enrichment at either time point. The authors should discuss potential technical or biological reasons for this unexpected result.

Thank you for your detailed review. By oversight, there was a mix-up between the data file for Fig.1B (H4Ac density of ODC at PE developmental timepoints) with that of Fig. 2B (H3K9Me3 density of DMO at PE developmental timepoints) resulting in swapping of the graphs. We have now corrected this and presented the right figures. This is an honest mistake from our end and we apologize for it. We once gain thank the reviewer for pointing out the discrepancy, which most likely would have skipped our attention. We have done a thorough check of all our data sets and found no other mistakes of similar kind.

Old

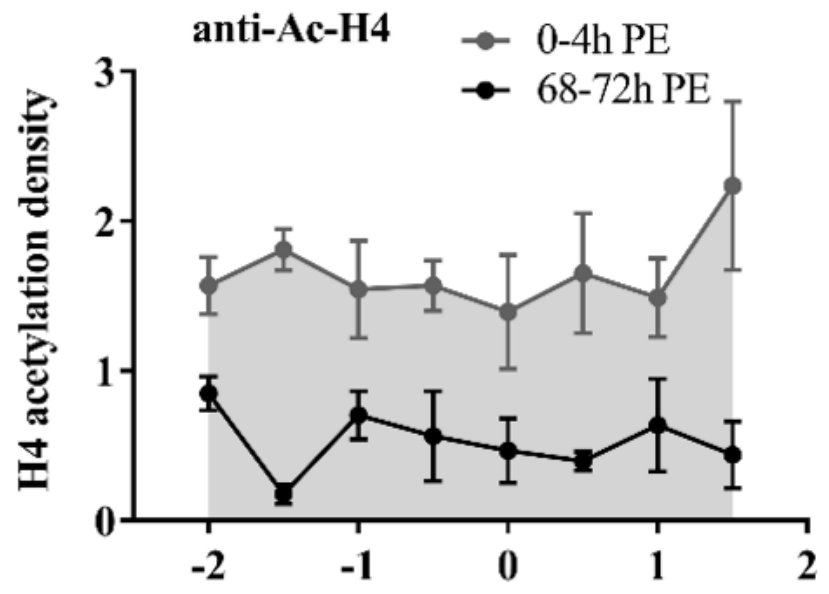

Fig 1B

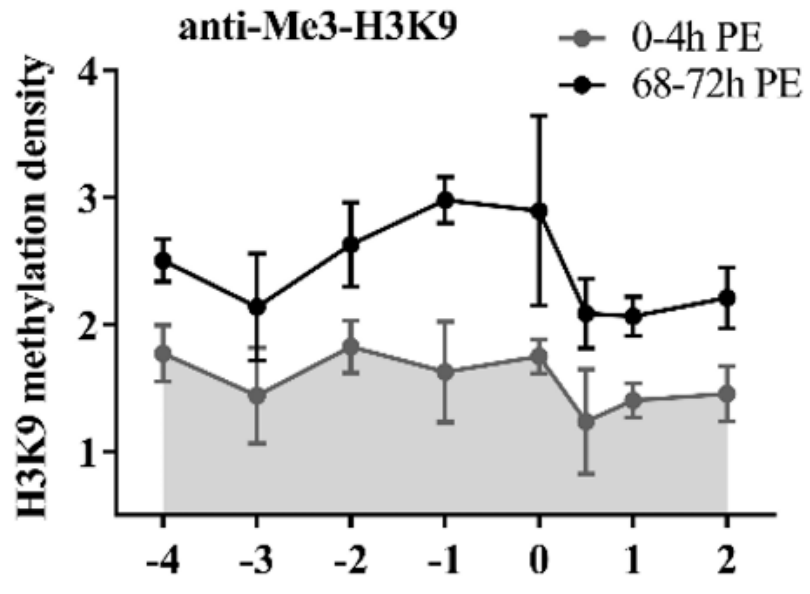

Fig 2B

New

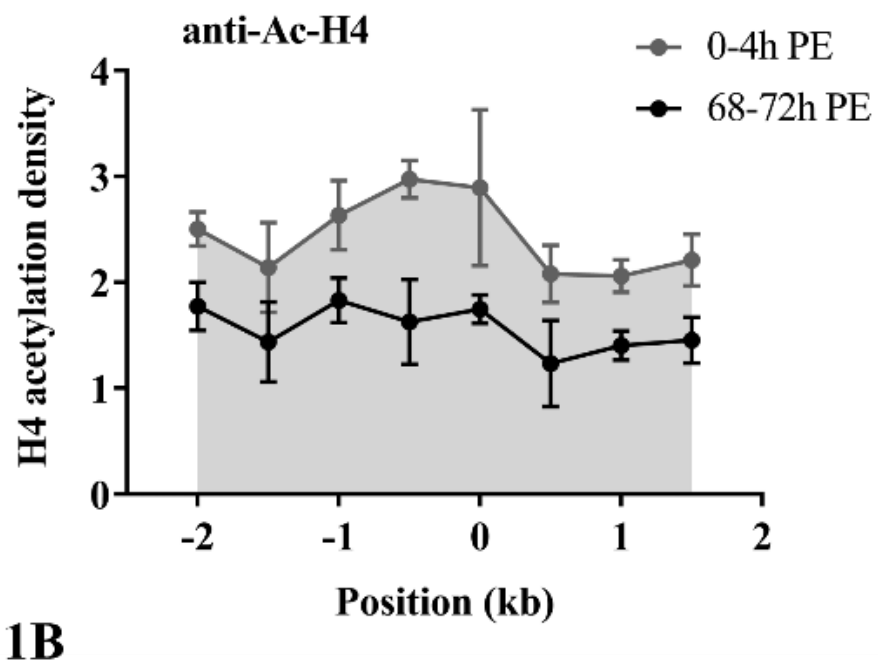

Fig 1B

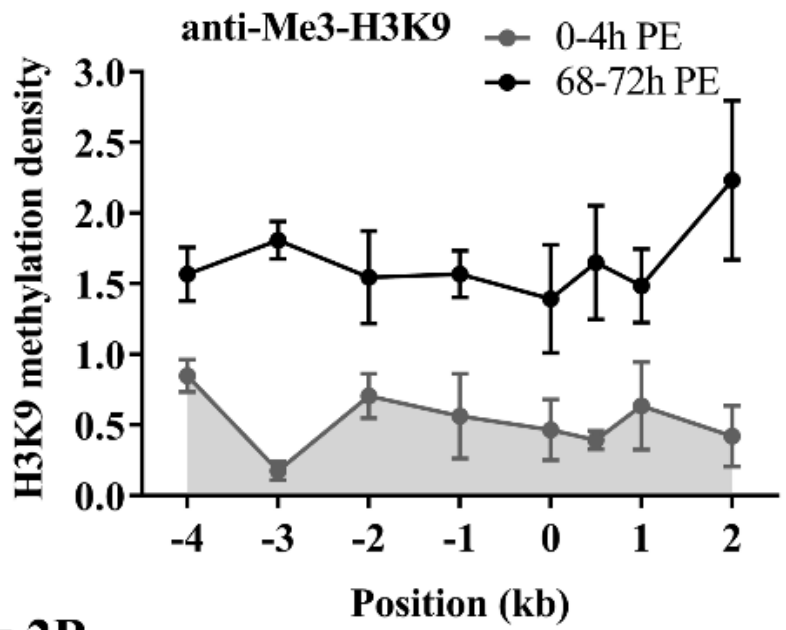

Fig 2B

5. Several experiments involve RNAi or topical JH treatment. The timing of sample collection following each treatment should be clearly stated in the figure legends to aid interpretation.

Thank you for the suggestion. The timing of dsRNA injection, ectopic JH treatment and sample collection is now clearly indicated in each figure legend.

6. Line 52: The description of the Met–Hsp83 interaction should be revised for accuracy. Current evidence indicates that Met is associated with Hsp83 in the absence of JH, and this complex dissociates upon JH binding.

Thank you for drawing our attention to this recent development. Taking reviewers suggestions we have altered the introduction to include- "In the cell, Met is either present as homodimer (Met-Met) or in association with the heat shock protein 83 (Hsp83) chaperon complex. Upon JH binding, Met dissociates and heterodimerize with another bHLH-PAS domain protein, Taiman (Tai), an insect homolog of the vertebrate steroid receptor co-activator (SRC)" at lines 51-54. The relevant manuscript detailing this observation (Tumova and Jindra; 2023) has been cited and referenced in the revised manuscript.

Reviewer #2 (Remarks to the Author):

The manuscript provides significant advances in understanding juvenile hormone (JH) signaling by elucidating how downstream repressors (Hairy and Kr-h1) drive chromatin-level repression in mosquitoes. Combines multiple approaches (ChIP-qPCR, MNase assays, RNAi, hormonal treatments) across developmental time points, the authors showed that Hairy and Kr-h1 induced alternative epigenetic modifications (H4 deacetylation vs. H3K9me3/chromatin compaction). While the specific combination of H4 deacetylation and H3K9me3 induced by JH repressors in mosquitoes have not been previously reported, the core epigenetic mechanisms

themselves (histone deacetylation and methylation as repressive marks) represent well-established, general paradigms for transcriptional repression by transcription factors. The study largely documents the occurrence of these common modifications rather than providing novel mechanistic insights into how Hairy and Kr-h1 specifically recruit or interact with the chromatin-modifying complexes (e.g., HDACs, HMTs) responsible. This lack of deeper mechanistic exploration into the recruitment and function of these modifiers limits the conceptual advancement. In addition, several key conclusions appear to extend beyond what the presented experimental evidence can robustly support.

Thank you for your positive comments and the criticism which we think are mostly valid. The histone modifications are quite conserved and we did not observe any deviation form already established core epigenetic mechanism. Indeed, we have documented the occurrence of established histone modifications as result of JH action in a specific but critically important developmental stage of dengue mosquito. This has given us platform to deep dive into the plausible mechanism of cumulative action between Kr-h1 and Hairy in the future. Our finding advances our understanding about how JH regulates gene networks and improves clarity about JH governed processes like mosquito reproduction and metamorphosis.

We acknowledge the concerns raised by the reviewer regarding the lack of evidence pertaining to recruitment and interaction with chromatin modifiers like HMTs and HDACs. Indeed, providing these evidences would make the entire story complete. We have been working on this for some time now and we plan to publish these results as a separate subsequent manuscript. We thought that the topic of associated chromatin modifying complexes deserve separate attention because of the complexity brought about by the combinatorial action of Hairy and Kr-h1, and the involvement of multiple co-factors for both. Furthermore, adding the entire data would make the current manuscript quite long and complex. Thus we decided to report the target gene chromatin changes associated with JH and its signalling pathway components in the first paper, and the details of the recruitment of chromatin modifying complexes in a subsequent paper. I would hope that the esteemed reviewer will find merit in our argument and kindly agree to let us follow through our plan.

Major Comments：

1.The claim of "synergistic action" between Hairy and Kr-h1 is not convincingly demonstrated. The data show additive or cumulative effects on repression for co-regulated genes (H4 deacetylation plus H3K9me3/compaction), but provide no evidence of biochemical cooperation or mutual enhancement between the factors themselves in inducing each other's modifications or binding. True synergy requires evidence that the combined effect exceeds the sum of individual effects, which is lacking here. The term "synergistic" should be replaced with "additive" or "cumulative".

Thank you for your thorough review. We agree with the reviewer that the observed effect is more cumulative/additive in nature rather than synergistic. The reason for this is historic, as we labelled the phenomenon as synergistic effect, albeit due to oversight, in our 2019 paper (Saha et al.; 2019). Taking reviewers suggestion, the term “synergistic” has been replaced with either

“cumulative” or “additive” throughout the manuscript in the revised manuscript. Accordingly, the Title of the manuscript has been altered as well.

2.The assertion that JH-Met induces modifications "through" Hairy or Kr-h1 implies a direct and necessary role. However, the critical rescue experiments (iHairy + JH; iKr-h1 + JH) to demonstrate that the absence of the factor abolishes the JH-induced modification are missing. The current knockdown data (iHairy or iKr-h1 alone) only show correlation, not causation. Without these controls, the conclusions appear somewhat far-fetched.

Thank you for pointing this out. Certainly, this is a critical evidence that we missed providing earlier. However, the suggested rescue experiments have been performed and provided with this revised version. iHairy and iKr-h1 mosquitoes were treated with JH (or control acetone) 48hrs post injection. Samples were collected 6hr post treatment followed by ChIP or MNase analysis as required. JH failed to induce the changes observed in *ODC*, *DMO* and *CSP* genes in the absence of the corresponding factor/s. The details have been provided in the revised manuscript lines 185-188, 226-227, 264-266, 308-310, 316-319, and 332-335. The corresponding figures are incorporated as Supplementary file, Fig. S5, S9 and S12. These set of experiments unambiguously establish that JH/Met induces the target gene chromatin changes via Hairy or Kr-h1.

3.The manuscript focuses on histone H3 occupancy as a proxy for overall histone position. Please clarify why histone H3, rather than H2A, H2B, H4, or a core histone pan-antibody, was specifically chosen for these experiments. Is there evidence suggesting H3 occupancy is the most representative or stable marker for general nucleosome positioning in this system?

To our understanding, there is no evidence that suggests that H3 occupancy is a better marker for overall histone positioning than other histone proteins such as H2A, H2B or H4. H3 was selected to proxy for histone positioning randomly based on prior research demonstrating the same (Li and Arnosti, 2011; Ha et al., 2019; Govind et al, 2011). To avoid confusion for the readers, we have altered the text from “histone positioning/histone occupancy” to “histone H3 occupancy” throughout the revised manuscript. Thank you for drawing our attention to this point.

4."histone position" in line 139 should be explicitly defined as referring to "histone H3 occupancy".

“Histone positioning” in line 139 and other places has been replaced with “histone H3 occupancy” as suggested.

5.Figure 1C/D/E and Figure 2C/D/E should indicate the time points of detection, whether it is at 72 h PE. If so, the apparent ~2-fold decrease in H4-Ac levels observed in the solvent (Fig.1C) or iLuc controls (Fig 1D/E) compared to the 72h PE time point in Fig 1B is concerning. A similar potential discrepancy seems present in the H3K9me3 data (Fig 2B vs Fig 2C/D/E). This raises questions about experimental consistency or normalization across different experimental sets (developmental time course vs. hormone treatment vs. knockdowns). Please address this potential inconsistency and ensure robust normalization strategies are applied uniformly across all datasets.

Hormonal applications were done on 4hr PE mosquitoes and tissue samples were collected 4-6hr post treatment. Thus, JH application experiments have a very different time frame and is not expected to mirror the 72hr PE developmental time point as such. For knockdown experiments, 24 h PE females were injected with suitable dsRNA and sample collections were done 48hr post injection. Although technically we are collecting samples at 72hr PE in this case, physical injury due to injection causes development delays in knockdown samples, including the control iLuc. Therefore, samples from our knockdown experiments does not mirror the 72hr PE developmental time point as well. Thus, taken together, both hormonal application experiments and knockdowns are precise tools to compare treatment and control groups, but does not correlate directly with the developmental time points. Therefore, variations are expected in the level of fold changes in H4-Ac or H3K9me3 between 72hr PE developmental time point and JH treated or knockdown samples. To give clarity to the readers, the details of the time points described above is now mentioned in the figure legends as per reviewer's suggestion.

6.Hairy binds ~1 kb upstream of the ODC TSS, and Kr-h1 binds ~3 kb upstream of the DMO TSS. It is therefore striking that JH, Met, or Hairy, Kr-h1 knockdowns induce changes in H4-Ac or H3K9me3 levels across all 8 assayed loci spanning promoter and coding regions. The manuscript should discuss potential mechanisms for this long-range effect. Does it involve chromatin spreading, looping, or recruitment of modifiers capable of acting over large distances? This is a significant finding that merits deeper discussion beyond just stating the observation.

Thank you for your comment. Indeed these are striking results that make our findings significant. Long range repression by Hairy is well reported in *Drosophila* (Barolo and Levine 1997, Martinez and Arnosti 2008, Li and Arnosti 2011, Kok et al, 2015). It has been shown that Hairy harbors a C-terminal WRPW domain that interacts and recruits co-repressor Groucho (Bianchi-Frias et al., 2004, Zhang and Levine 1999, Martinez and Arnosti 2008). We reported Hairy-Groucho interaction in mosquitoes and their role in repression of target genes, including *ODC* (Saha et al., 2016). In *Drosophila*, Groucho forms complexes that include class-I HDAC Rpd3 machinery that deacetylates the lysine residues of all four core histones of the target gene. The co-repressor is demonstrated to be recruited in transcribed region of gene, acting several kilobases downstream of the repressor binding site (Martinez and Arnosti 2008). A Groucho mediated "spreading" mechanism has been suggested for *Drosophila* resulting in chromatin modification over extensive regions leading to transcriptional inhibition (Song 2004, Martinez and Arnosti 2008). We expect a similar mechanism to be active in case of JH/Met mediated gene repression through Hairy.

Direct evidence of Kr-h1's role in long range repression is lacking. However, mammalian homolog Krüppel like factor (KLF) is known to recruit different chromatin remodelers including heterochromatin protein 1 (HP1) and its associated HMT protein, SUV39H1 (Lomberk, 2012; Seo, 2012). This HP1-HMT complex is known to methylate adjacent histone H3K9 residues. Interestingly, HP1 is recruited to these H3K9-Me3 marks leading to long distance spreading of repressive methylation through the chromatin (Hwang, 2001; Lomberk, 2006; Hamali 2023). Additionally, another class of protein belonging to the

C2H2 Zinc finger family- Krüppel associated box domain-zinc finger protein (KRAB-ZFPs) has been demonstrated to induce H3K9 trimethylation via KRAB associated protein 1 (KAP1) resulting in chromatin compaction several kb away from transcription factor binding site (Groner, 2010). This process is known to involve the histone methyltransferase SETDB1 and HP1 (Groner, 2010; Hamali, 2023). It is likely that in mosquitoes, Kr-h1 mediated repression involves components of the above discussed pathways, including HP1, for the observed long range effect. I would like to point out that we are currently in the process of characterizing the co-factors and chromatin remodelers involved in Hairy and Kr-h1 mediated gene repression downstream of JH/Met. A clear understanding about the mechanism of repression, including the cumulative action of the two factors, are only possible after these sets of experiments. These results will be published as a subsequent manuscript after the one that is currently under consideration.

The details have been provided in the discussion section of the revised manuscript lines 372 to 400.

7.Several procedural details currently in the Results section (e.g., antibody sources, statistical method) are more appropriate for the Methods section. The Results should focus on presenting and interpreting the findings, while ensuring all necessary methodological information is accurately and concisely detailed in the dedicated Methods section.

Thank you for your comment. Procedural details, as suggested, has been now shifted to the methods section in the revised manuscript.

8.Language Clarity and Flow:

a) Line 62: Ambiguous pronoun: "Unlike Met-mediated gene activation, its repression is indirect..." Change to: "Unlike Met-mediated gene activation, repression mediated by Met is indirect, requiring the involvement of additional intermediate factors."

As per the suggestion of the reviewer, the sentence has been replaced.

b) Line 119: Inaccurate contrast: "In contrast, DMO has a Kr-h1 binding site (KBS)..." Replace with a neutral connector: "Separately, DMO has a Kr-h1 binding site (KBS)..." or "For DMO, a Kr-h1 binding site (KBS) was located..."

As per reviewer's suggestions, the sentence has been changed and incorporated in Line 127-129.

c) Line 209: "Similarly, a clear induction in H3K9 trimethylation was observed in Tep3 gene loci by JH and Kr-h1..." Rewrite this sentence to describe the Kr-h1 RNAi results.

Thank you for your suggestion. We have replaced the sentence with –"Similarly, a clear induction in H3K9 trimethylation was observed in *Tep3* gene loci at 72h PE as well as by JH; whereas RNAi mediated knockdown of Met and Kr-h1 significantly decrease H3K9 trimethylation at all tested locus. This is incorporated in Lines 230-232 in the revised manuscript.

Reviewer #3 (Remarks to the Author):

Authors did not show the evidence of the binding of Hairy and kr-h1 on the binding sites. Authors did not show the evidence of recruitment of cofactors or histone. Authors need to explain the results of chip analysis, therefore it is not easy to understand so many acetyl- H4 or H3K9me3 around the target gene locus. The trial is important but the methods and the explanations are not enough.

Thank you for your comments. The binding of Hairy and Kr-1 to ODC and CSP has already been demonstrated in great details (Please see response to editors comments Point 1). Additionally, we have now performed ChIP studies to demonstrate Kr-h1 binding to identified motifs in the promoter of DMO gene (Response to editor Point 1). We acknowledge reviewer's concern regarding evidence of recruitment of cofactors or histones. We have been working on this for some time now. We have provided a detailed rationale as to why this was skipped in the current manuscript in Response to editors comments Point 3. With all due respect, I would disagree with the comment that adequate explanation for the ChIP analysis was not provided. We firmly believe that we have provided all necessary details in results and Methods section and figure legends to interpret the results represented clearly.

Reference:

1. Saha, T. T. et al. Hairy and Groucho mediate the action of juvenile hormone receptor Methoprene-tolerant in gene repression. Proc Natl Acad Sci U S A. 113(6), E735–43 (2016).
2. Ojani R, Fu X, Ahmed T, Liu P, Zhu J. Krüppel homologue 1 acts as a repressor and an activator in the transcriptional response to juvenile hormone in adult mosquitoes. Insect Mol Biol. 27(2), 268–78 (2018).
3. Saha, T. T. et al. Synergistic action of the transcription factors Krüppel homolog 1 and Hairy in juvenile hormone/Methoprene-tolerant-mediated gene-repression in the mosquito *Aedes aegypti*. *PLoS Genet*. **15(10),** 1–23 (2019).
4. Tumova, S., Jindra, M. Ligand-dependent protein interactions of the juvenile hormone receptor captured in real time. *FEBS J.* **290(11),** 2881–94 (2023).
5. Li, L. M., Arnosti, D. N. Long- and short-range transcriptional repressors induce distinct chromatin states on repressed genes. *Curr Biol*. **21(5),** 406–12 (2011).
6. Ha, S.D. et al. Transcription-dependent targeting of Hda1C to hyperactive genes mediates H4-specific deacetylation in yeast. *Nat Commun.* **10(1),** 4270 (2019).
7. Govind, C.K. et al. Phosphorylated Pol II CTD recruits multiple HDACs, including Rpd3C(S), for methylation-dependent deacetylation of ORF nucleosomes. *Mol Cell.* **39(2),** 234-46 (2010).
8. Barolo, S., Levine, M. hairy mediates dominant repression in the Drosophila embryo. EMBO J. 16(10), 2883-91 (1997).
9. Martinez, C. A., Arnosti, D. N. Spreading of a Corepressor Linked to Action of Long-Range Repressor Hairy. Mol Cell Biol. 28(8), 2792–802 (2008).

10. Kok, K., Ay, A., Li, L. M., Arnosti, D. N. Genome-wide errant targeting by Hairy. Elife. 4, e06394 (2015).
11. Bianchi-Frias, D. et al. Hairy transcriptional repression targets and cofactor recruitment in Drosophila. PLoS Biol. 2(7), E178 (2004).
12. Zhang, H., Levine, M. Groucho and dCtBP mediate separate pathways of transcriptional repression in the Drosophila embryo. Proc Natl Acad Sci U S A. 96(2), 535-40 (1999).
13. Song, H., Hasson, P., Paroush, Z., Courey, A. J. Groucho oligomerization is required for repression in vivo. *Mol Cell Biol.* **24(10),** 4341-50 (2004).
14. Lomberk, G. al. Sequence-specific recruitment of heterochromatin protein 1 via interaction with Krüppel-like factor 11, a human transcription factor involved in tumor suppression and metabolic diseases. J Biol Chem. 287(16), 13026–39 (2012).
15. Seo, S. et al. Krüppel-like factor 11 differentially couples to histone acetyltransferase and histone methyltransferase chromatin remodeling pathways to transcriptionally regulate dopamine D2 receptor in neuronal cells. J Biol Chem. 287(16), 12723–35 (2012).
16. Hwang, K. K., Eissenberg, J. C., Worman, H. J. Transcriptional repression of euchromatic genes by Drosophila heterochromatin protein 1 and histone modifiers. Proc Natl Acad Sci U S A. 98(20), 11423–7 (2001).
17. Lomberk, G., Urrutia, R. The family feud: Turning off Sp1 by Sp1-like KLF proteins. Biochem J. 392(1), 1–11 (2005).
18. Hamali, B., Amine, A. A. A., Al-Sady, B. Regulation of the heterochromatin spreading reaction by trans-acting factors. Open Biol. 13(11), 230271 (2023).
19. Groner, A. C. et al. KRAB-zinc finger proteins and KAP1 can mediate long-range transcriptional repression through heterochromatin spreading. PLoS Genet. 6(3), e1000869 (2010).

Point-by-point response to the reviewers

Editor's Comments:

Your manuscript entitled "Juvenile hormone-mediated chromatin remodeling: insights into cumulative action of downstream transcription factors, Hairy and Krüppel homolog 1" has now been seen by 2 referees, whose comments are appended below. You will see from their comments below that while they find your work of considerable interest, an important point is raised. We are interested in the possibility of publishing your study in Communications Biology, but would like to consider your response to this concern in the form of a revised manuscript before we make a final decision on publication.

We therefore invite you to revise and resubmit your manuscript, taking into account the points raised. In particular, given the variability of RNAi silencing upon thoracic injection, we ask that you demonstrate, and report, knockdown efficiency of your RNAi assays.

We sincerely thank you for considering our manuscript and for your valuable comments. We acknowledge the concerns raised by the reviewers and editors regarding the efficiency of our RNAi assays. We would like to clarify that the knockdown samples were indeed tested by qPCR for mRNA expression of the respective genes before processing for ChIP or MNase protection assays. We did not furnish the results thinking that it might not be of interest to the readers. Now, we have included the same as Supplementary figure 14 of the Supplementary file. Corresponding description about the validation of dsRNA mediated target gene knockdowns have been incorporated in Lines 466 to 470 in the methods section under the subheading- 'dsRNA-mediated gene silencing' in the revised manuscript.

Reviewers' comments:

Reviewer #1 (Remarks to the Author):

The revised manuscript has addressed many of the concerns raised in the previous reviews and shows significant improvement.

However, the authors did not verify the knockdown efficiency in their RNAi experiments. Ideally, both mRNA and protein levels of Kr-h1 and Hairy should be assessed. Instead, the authors rely on data from their earlier publications to suggest successful RNAi, but those results are not from the current experiment and therefore can't be considered applicable here.

Thank you for your positive comments. As per reviewer's suggestion, we have furnished results demonstrating depletion of respective mRNA levels in dsRNA treated samples in the revised manuscript. The results can be found as Supplementary figure 14 of the Supplementary file. Corresponding description about the results have been incorporated in Lines 466 to 470 in the methods section under the subheading- 'dsRNA-mediated gene silencing'. However, we had not collected protein samples while performing the experiments and therefore, we are not in a position to furnish protein results. We acknowledge that in the best case scenario, protein

measurements should be included to establish knockdown efficacy. We firmly believe that depletion of target gene mRNA post dsRNA treatment is sufficient to establish our case.

Reviewer #2 (Remarks to the Author):

The authors have adequately addressed all my concerns. I support acceptance.

We sincerely thank you for taking time out to review our manuscript. We are glad that we could address all the concerns pertaining to the manuscript.
