# Supplementary material for: Juvenile hormone-mediated chromatin remodelling by cumulative action of downstream transcription factors, Hairy and Krüppel homolog 1
Source: Commun Biol. 2026 May 26;9:1214. doi: 10.1038/s42003-026-10327-4 (PMC13586389; doi:10.1038/s42003-026-10327-4)
Supplement: Supplementary file 2 [file 42003_2026_10327_MOESM2_ESM.pdf]

Supplementary Information

Supplementary Figure S1. Expression analysis of Hairy and Kr-h1 target genes.

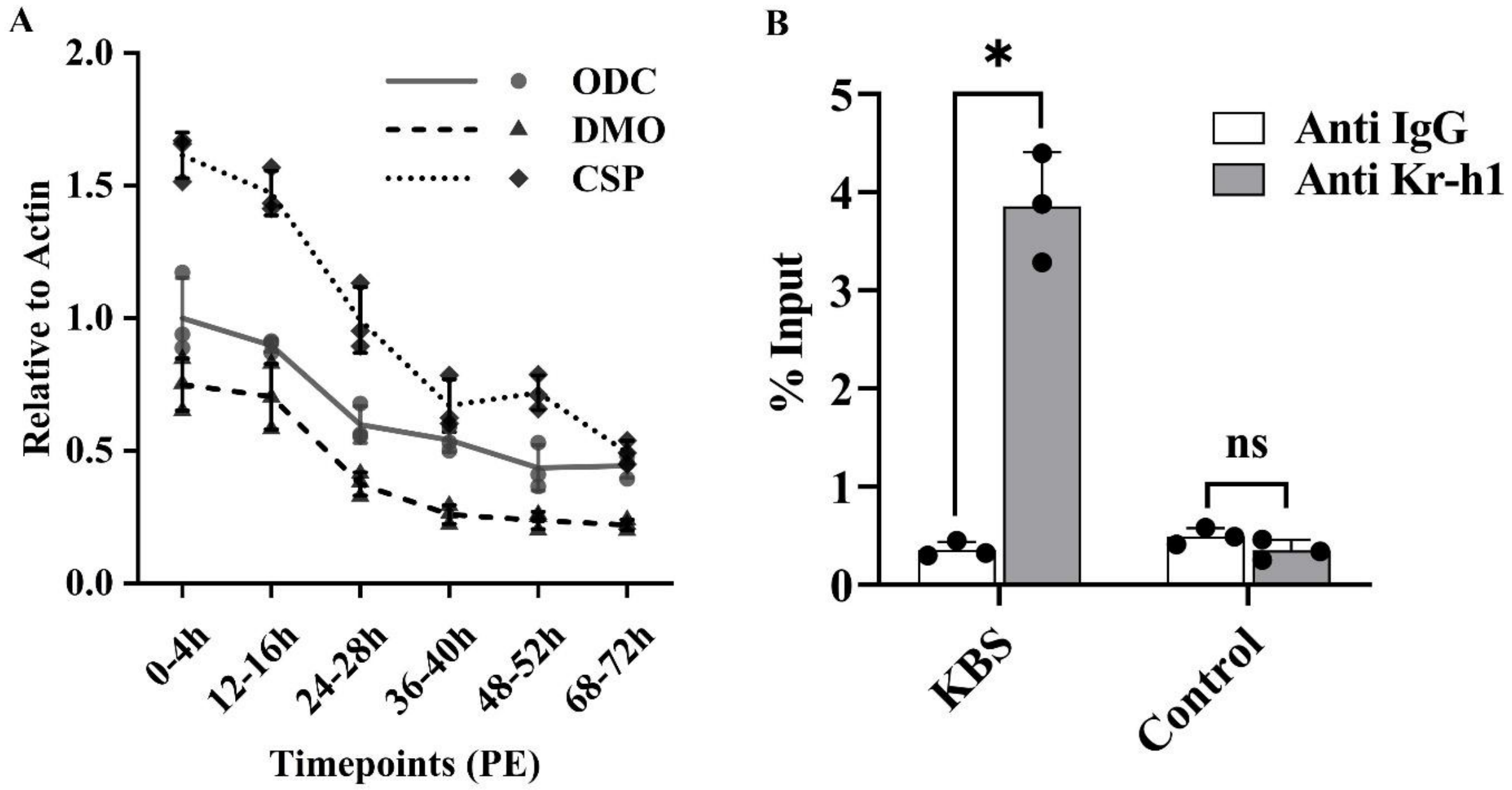

A) Temporal expression profiles of *Ornithine decarboxylase (ODC, AAEL000044), Dimethylaniline monooxygenase (DMO, AAEL000834)* and *Clip domain serine protease (CSP, AAEL005093)* at 6 different timepoints during PE development as mentioned in the graphs, as measured using qPCR. **B)** ChIP assays demonstrating the binding of Kr-h1 to its binding site in the promoter of target gene *DMO*. The 1.4 kb promoter region containing the potential KBS of Met/Kr-h1 target gene *DMO* was cloned in pGL3 Basic vector and transfected into Drosophila S2 cells along with plasmid overexpressing Kr-h1. ChIP were performed with *Aedes* anti-Kr-h1 antibody and anti-IgG antibody (control). Quantifications were performed by qPCR using primer pairs targeting KBS regions in the target gene promoter. Primers targeting the plasmid backbone were utilized as controls. Data was represented as % of input DNA.

Error bars represent ± SD. Three biological replicates were performed for all experiments with similar results. Source data are provided in Supplementary Data 1. (* $p < 0.05$; ns- not significant)

Supplementary Figure S2. Histone H3 occupancy map of target gene *ODC*.

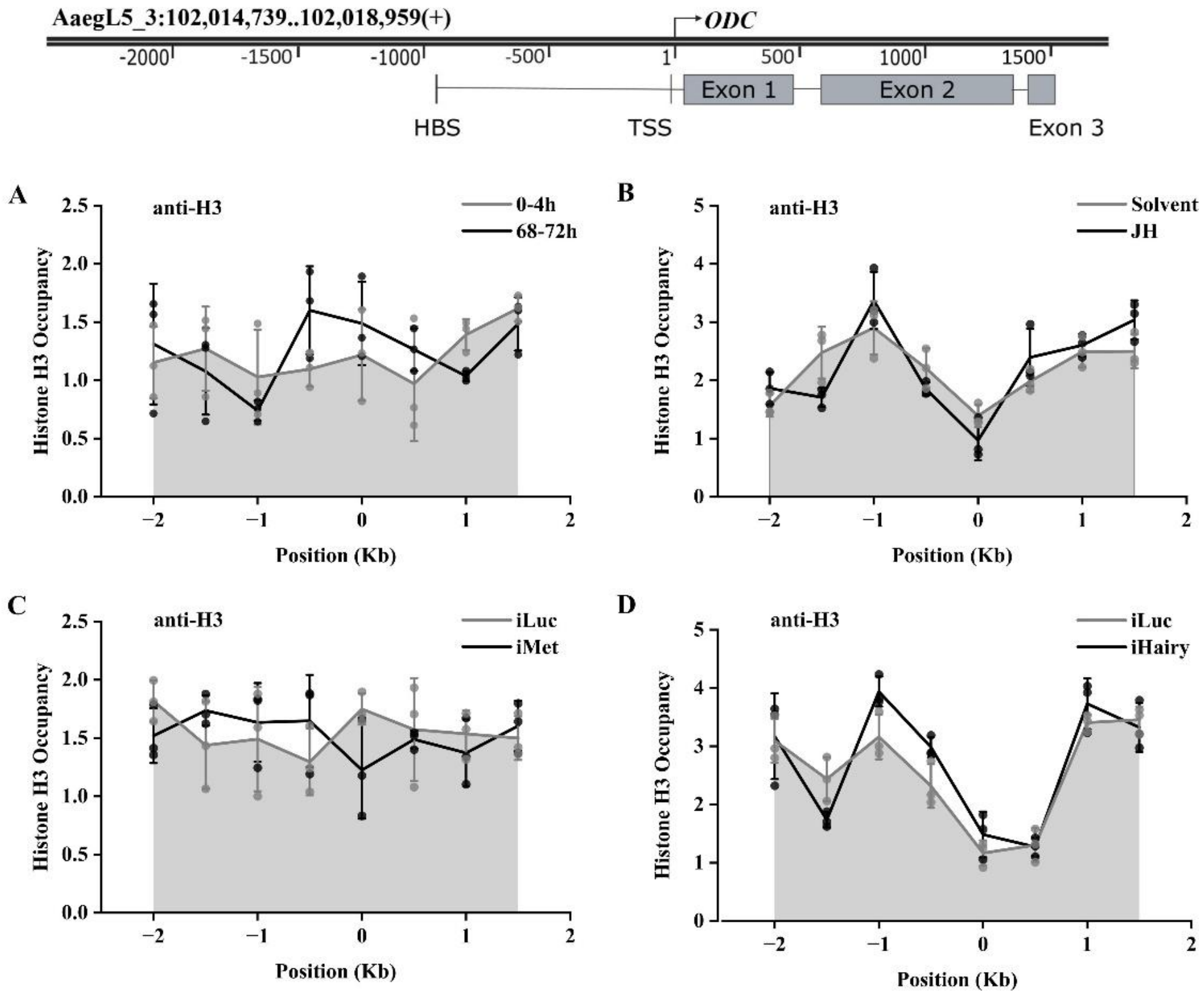

(A) PE reproductive maturation does not alter the overall histone occupancy of the target gene, as evidenced by similar histone position profiles at 12 h and 72 h PE. **(B)** JH has no significant impact on histone H3 positioning across the various regions of target gene loci. **(C and D)** dsRNA-mediated knockdown of neither *Met* nor *Hairy* impacts the histone positioning of the target gene *ODC*.

For ectopic JH treatment, sample collection was done 6 h post treatment. For knockdown, dsRNA injection was performed on 24 h PE adult female mosquitoes followed by sample collection 48 h post injection. Histone H3 occupancy for *ODC* gene was assayed by ChIP-qPCR using anti-H3 antibody. histone H3 occupancy was calculated by normalising the immunoprecipitation signal with the histone H3 occupancy level at the actin promoter. Error bars represent ± SD. Three biological replicates were performed for all experiments with similar results. Source data are provided in Supplementary Data 1.

Supplementary Figure S3. Histone H3 occupancy map of target gene *DMO*.

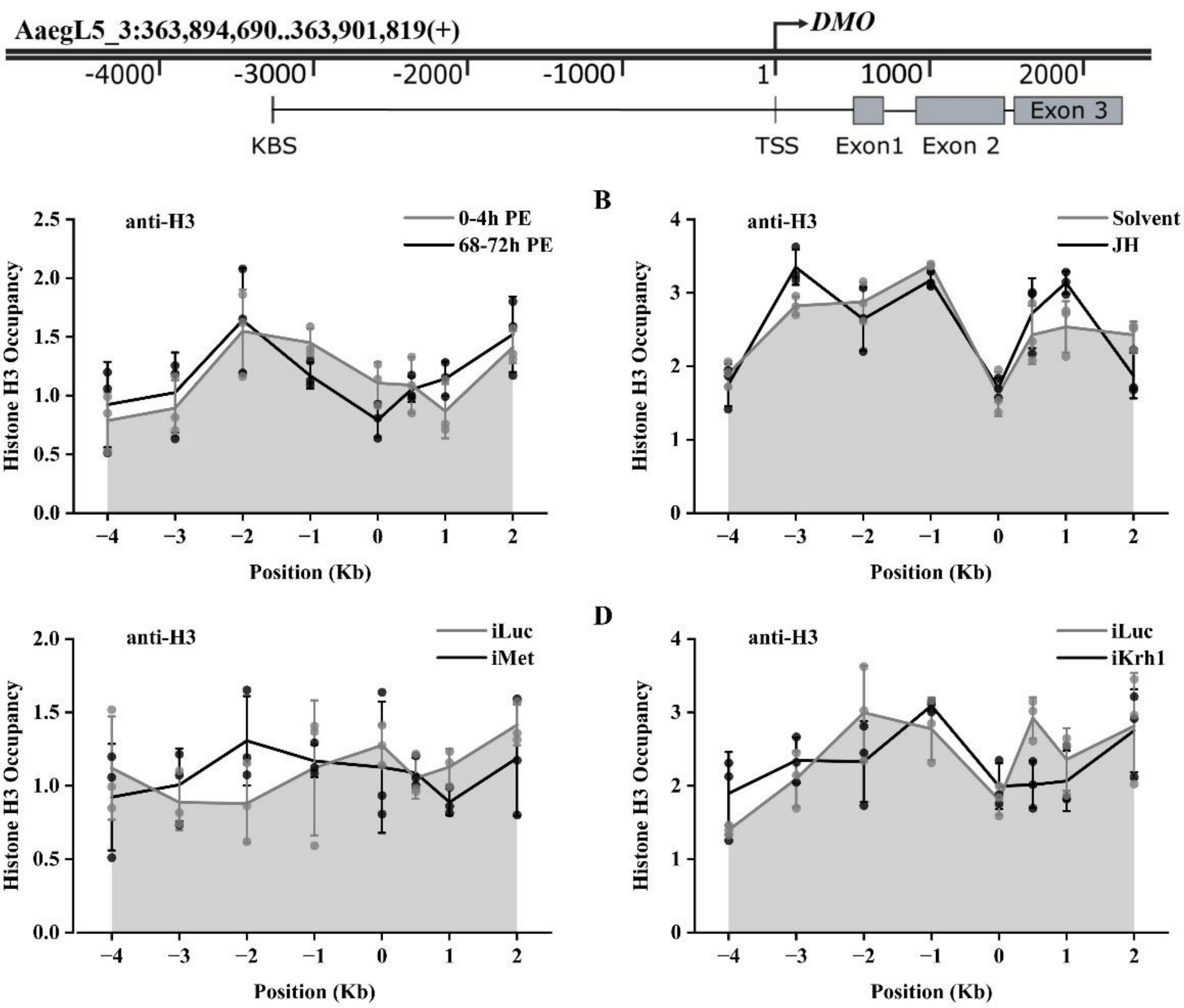

(A) PE reproductive maturation does not alter the overall histone occupancy of the target gene, as evidenced by similar histone H3 position profiles at 12 h and 72 h PE. **(B)** JH has no significant impact on the histone H3 positioning across the various regions of target gene loci. **(C and D)** dsRNA-mediated knockdown of neither *Met* nor intermediate factor *Kr-h1* impacts the histone positioning of the target gene *DMO*.

For ectopic JH treatment, sample collection was done 6 h post treatment. For knockdown, dsRNA injection was performed on 24 h PE adult female mosquitoes followed by sample collection 48 h post injection. Histone H3 occupancy of target gene *DMO* was assayed by ChIP-qPCR using anti-H3 antibody. Histone H3 occupancy was calculated by normalizing the immunoprecipitation signal with the histone H3 occupancy level at the actin promoter. Error bars represent ± SD. Three biological replicates were performed for all experiments with similar results. Source data are provided in Supplementary Data 1.

Supplementary Figure S4. Histone H4-Ac map of *DMO*.

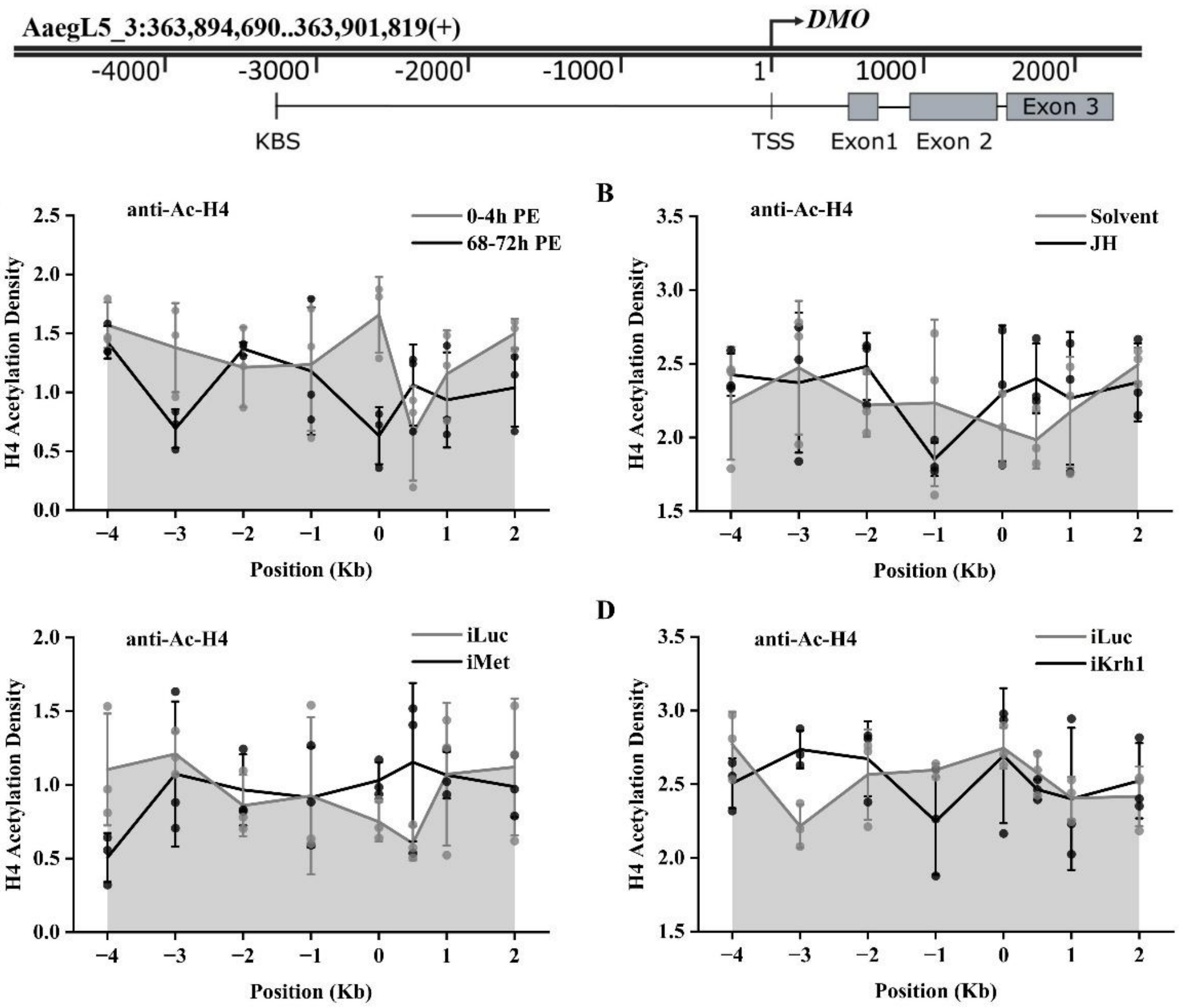

(A and B) No change in histone H4-Ac is observed during PE development time points or with ectopic JH application at almost all the loci of the gene *DMO*. **(C and D)** dsRNA-mediated knockdown of *Met* and *Kr-h1* results in no alteration of histone H4-Ac pattern on the gene.

For ectopic JH treatment, sample collection was done 6 h post treatment. For knockdown, dsRNA injection was performed on 24 h PE adult female mosquitoes followed by sample collection 48 h post injection. Histone H4-Ac levels were assayed by ChIP-qPCR using an anti-Ac-H4 antibody for all experiments. H4-Ac density was calculated from ChIP samples by normalizing the immunoprecipitation signal first with the H4-Ac level at the actin promoter and then with the relative H3 level. Error bars represent ± SD. Three biological replicates were performed for all experiments with similar results. Source data are provided in Supplementary Data 1.

Supplementary Figure S5. *Hairy* knockdown compromises the JH-Met mediated H4 deacetylation of *ODC* gene.

A

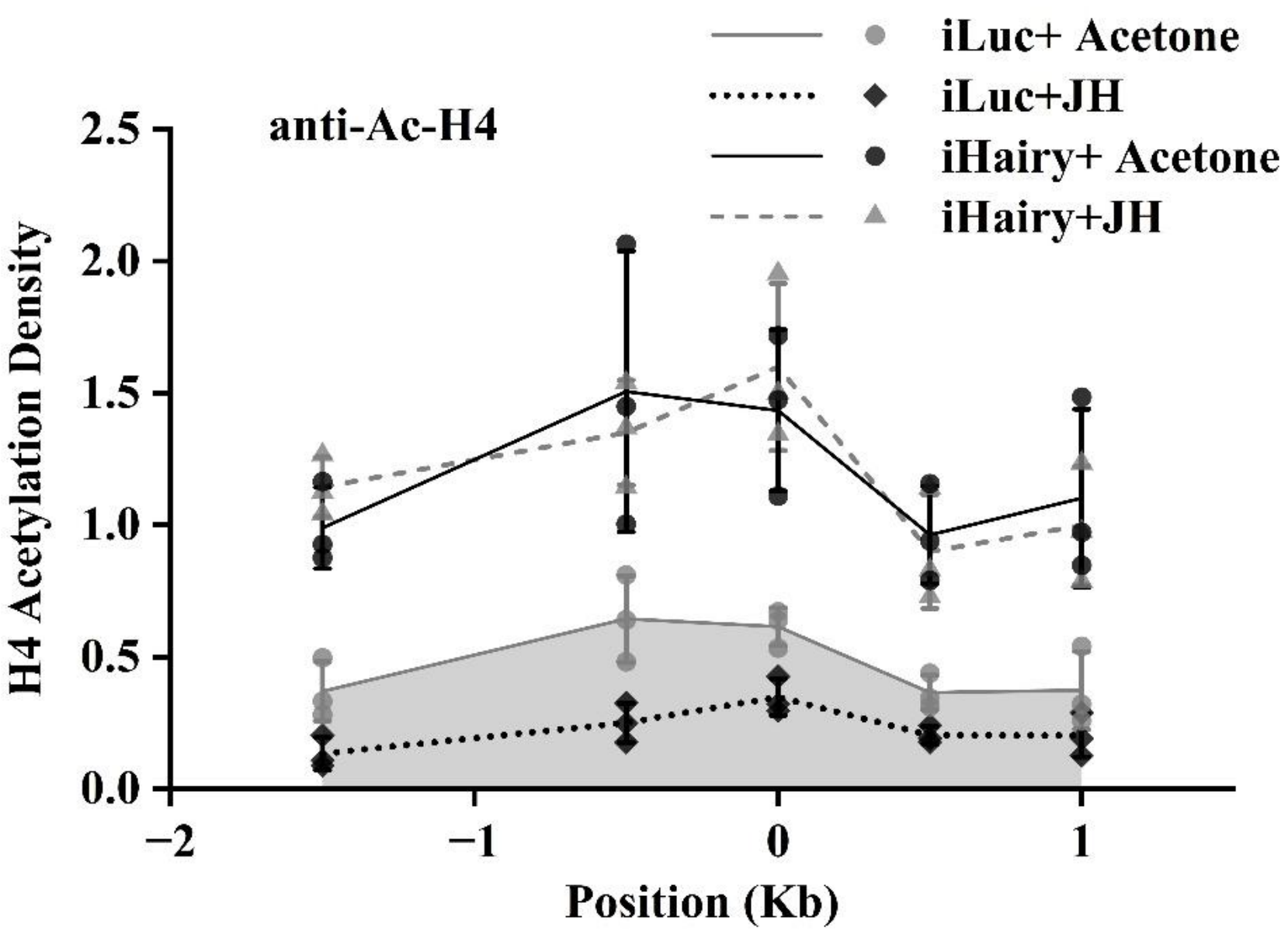

Ectopic JH application has no significant effect in H4 acetylation density at any locus of *ODC* gene in iHairy mosquitoes. However, JH induced significant H4 deacetylation in *ODC* gene in control iLuc mosquitoes. dsRNA injection was performed on 24 h PE adult female mosquitoes followed by JH or acetone (control) treatment 48 h post injection. Samples were collected 6 h post JH treatment for ChIP-qPCR using an anti-Ac-H4 antibody. H4-Ac density was calculated from ChIP samples by normalizing the immunoprecipitation signal first with the H4-Ac level at the actin promoter and then with the relative H3 level. Error bars represent ± SD. Three biological replicates were performed for all experiments with similar results. Source data are provided in Supplementary Data 1.

Supplementary Figure S6. Chromatin architecture of target gene *Matrix metallo-proteinase (MMP)*

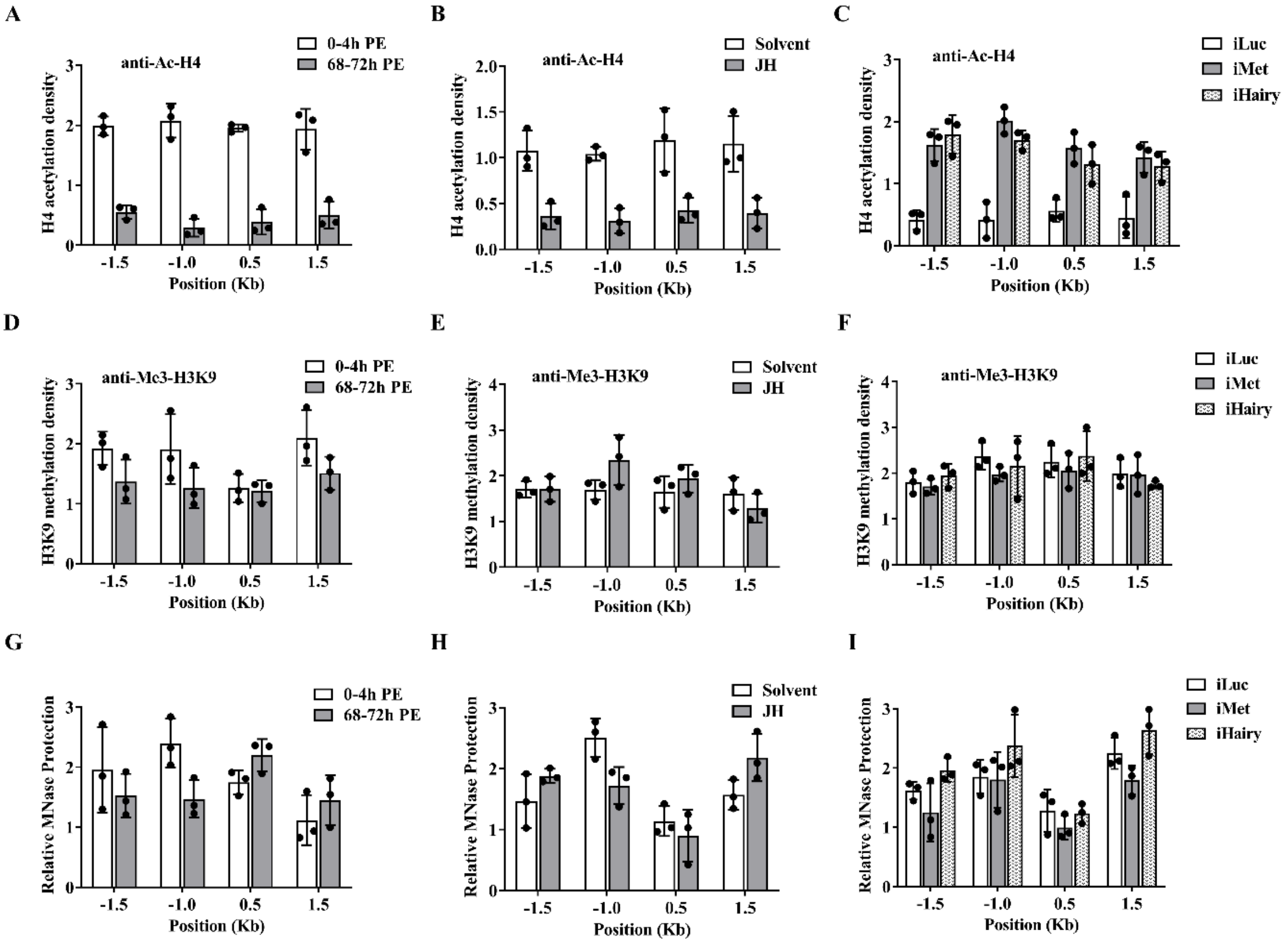

(A and B) Significant reduction in histone H4-Ac marks was observed at 72 h PE and after JH treatment at all the *MMP* gene loci**. (C)** RNAi knockdown of JH receptor *Met* and the downstream factor *Hairy* significantly increase the H4-Ac marks at all the loci. **(D and E)** Histone H3K9-Me3 marks were not affected at different time points of PE maturation and after JH treatment. **(F)** No significant change in H3K9-Me3 marks was also observed in iMet and dairy samples. **(G-I)** MNase digestion profile of the *MMP* gene also remains unaltered after PE maturation and JH treatment, as well as in *Met* and *Hairy* knockdown samples.

For ectopic JH treatment, sample collection was done 6 h post treatment. For knockdown, dsRNA injection was performed on 24 h PE adult female mosquitoes followed by sample collection 48 h post injection. For graphs A-F, H4 acetylation density and histone H3K9-Me3 density w measured by ChIP assay using anti-Ac-H4 and anti-H3K9-Me3 antibody. H4

acetylation and H3K9 trimethylation density were calculated from ChIP samples by normalizing the immunoprecipitation signal first with the H4-Ac or H3K9-Me3 level at the actin promoter and then with the relative H3 level. For graphs G-I, the relative MNase protection level was calculated by comparing the level of digested samples with undigested controls. Error bars represent ± SD. Three biological replicates were performed for all experiments with similar results. Source data are provided in Supplementary Data 1.

Supplementary Figure S7. Chromatin architecture of target gene *Thioester-containing protein 3 (Tep3)*

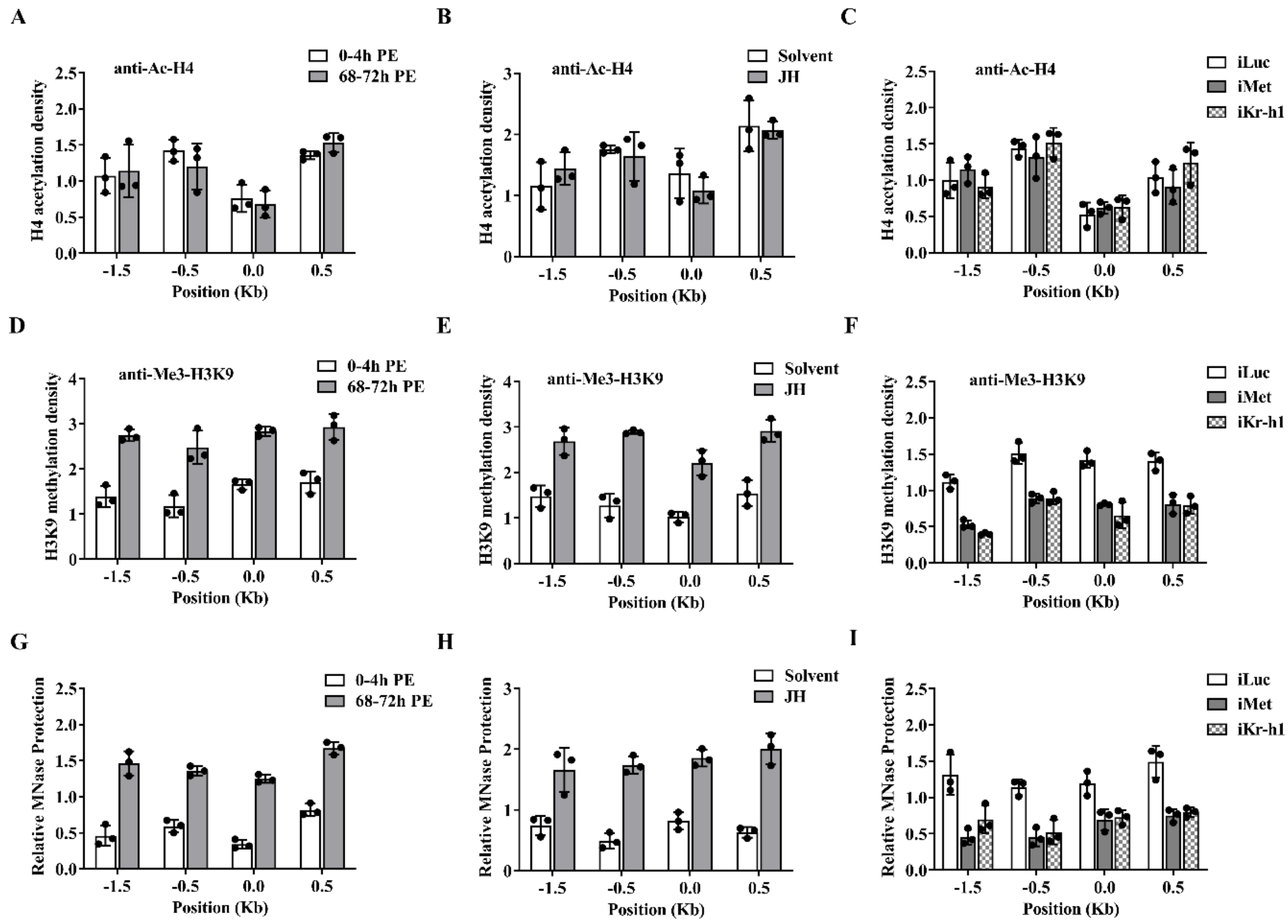

(A and B) No significant change in H4-Ac was observed at different PE time points and after JH treatment. **(C)** RNAi knockdown of JH receptor *Met* and the downstream factor *Kr-h1* does not alter the H4-Ac density of the *Tep3* gene at any loci. **(D and E)** Histone H3k9-Me3 marks were significantly increased at 72 h PE, and JH-treated samples in comparison to the controls. **(F)** iMet and iKr-h1 samples show a significant reduction in the H3K9-Me3 marks compared to iLuc control at all the loci of the *Tep3* gene. **(G and H)** MNase sensitivity was significantly

reduced in 72 h PE and JH-treated samples compared to controls, throughout all the assayed loci. **(I)** Knockdown of *Met* and *Kr-h1* induces digestion of target gene loci by MNase concerning iLuc controls.

For ectopic JH treatment, sample collection was done 6 h post treatment. For knockdown, dsRNA injection was performed on 24 h PE adult female mosquitoes followed by sample collection 48 h post injection. For graphs A-F, H4 acetylation density and histone H3K9-Me3 density was measured by ChIP assay using anti-Ac-H4 and anti-H3K9-Me3 antibody. H4 acetylation and H3K9 trimethylation density were calculated from ChIP samples by normalizing the immunoprecipitation signal first with the H4-Ac or H3K9-Me3 level at the actin promoter and then with the relative H3 level. For graphs G-I, the relative MNase protection level was calculated by comparing the level of digested samples with undigested controls. Error bars represent ± SD. Three biological replicates were performed for all experiments with similar results. Source data are provided in Supplementary Data 1.

Supplementary Figure S8. Histone H3K9-Me3 density of target gene *ODC*.

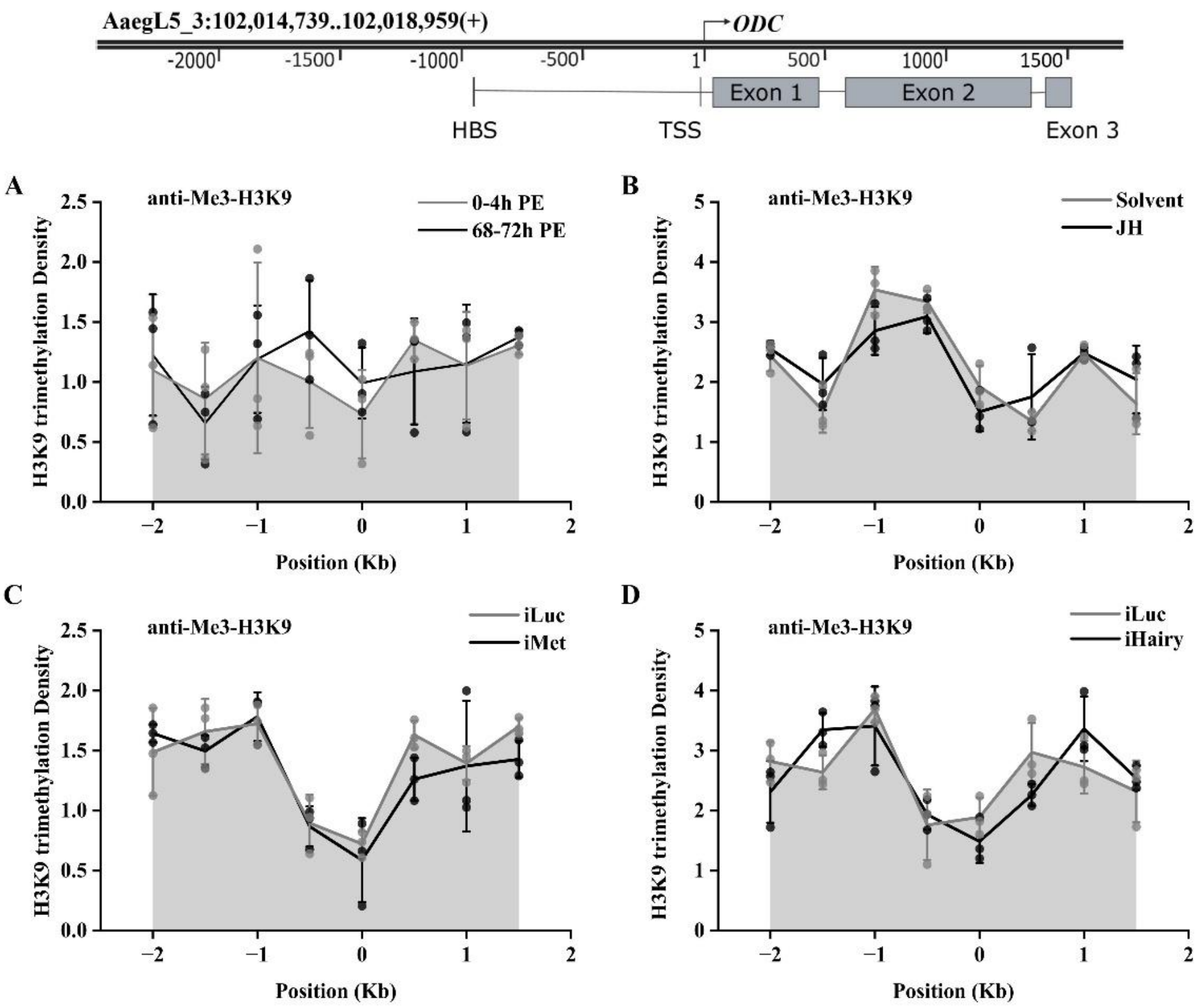

(A and B) No significant change in histone H3K9-Me3 level is observed on any loci of the *ODC* gene during PE development or after treatment with exogenous JH. **(C and D)** Knockdown of JH receptor *Met* and intermediate factor *Hairy* results in no alteration in the H3K9-Me3 level throughout the loci of *ODC* gene.

For ectopic JH treatment, sample collection was done 6 h post treatment. For knockdown, dsRNA injection was performed on 24 h PE adult female mosquitoes followed by sample collection 48 h post injection. The H3K9-Me3 density of target gene *ODC* was measured by ChIP assay using anti-H3K9-Me3 antibody for all experiments. H3K9-Me3 density was calculated from ChIP samples by normalizing the immunoprecipitation signal first with the H3K9-Me3 level at the actin promoter and then with the relative H3 level. Error bars represent ± SD. Three biological replicates were performed for all experiments with similar results. Source data are provided in Supplementary Data 1.

Supplementary Figure S9. *Kr-h1* knockdown compromises the JH-Met mediated H3K9 trimethylation and chromatin compaction of *DMO*.

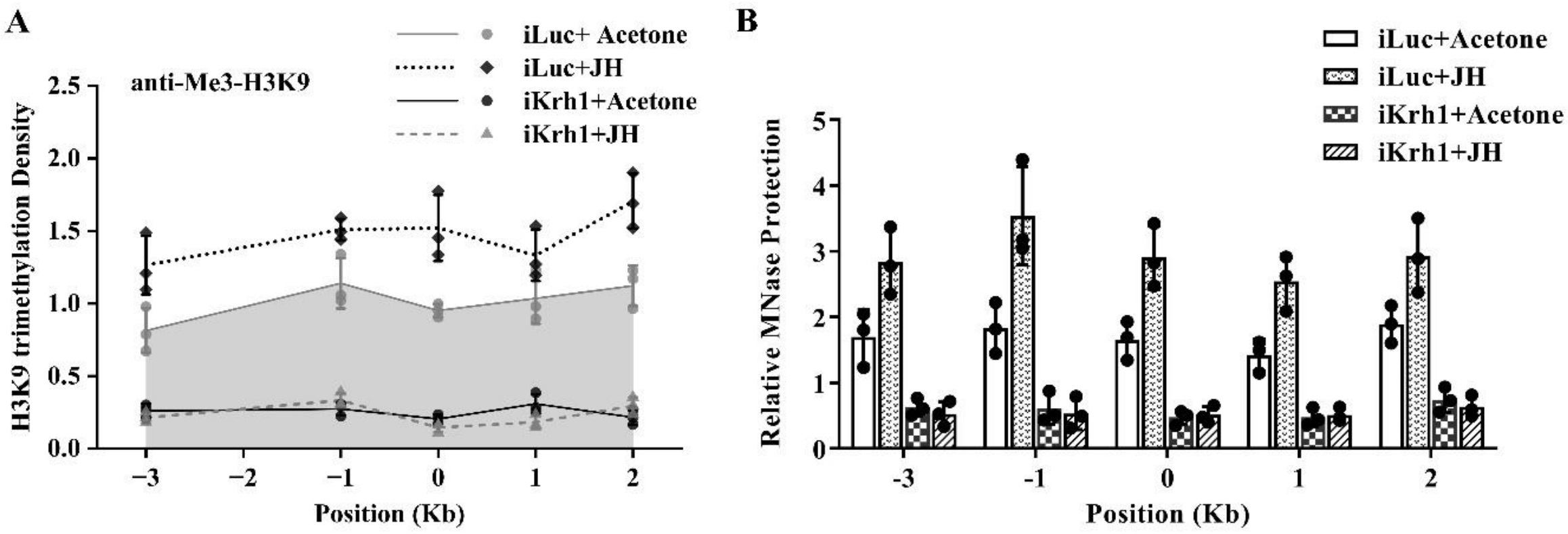

A) Ectopic JH application has no significant effect in H3K9 trimethylation density at any locus of *DMO* gene in iKr-h1 mosquitoes. However, JH induced significant H3K9 trimethylation in *DMO* gene in control iLuc mosquitoes. ChIP-qPCR was done using anti-Me3 H3K9 antibody. H3K9-Me3 density was calculated from ChIP samples by normalizing the immunoprecipitation signal first with the H3K9-Me3 level at the actin promoter and then with the relative H3 level. **B)** Ectopic JH application does not impact the overall MNase sensitivity of *DMO* gene after dsRNA mediated knockdown of *Kr-h1*. In iLuc control, ectopic JH application significant increase relative MNase protection in all tested loci of *DMO* gene. Relative MNase protection level was calculated by comparing the level of digested samples with undigested controls.

dsRNA injection was performed on 24 h PE adult female mosquitoes followed by JH or acetone (control) treatment 48 h post injection. Samples were collected 6 h post JH treatment. Error bars represent ± SD. Three biological replicates were performed for all experiments with similar results. Source data are provided in Supplementary Data 1.

Supplementary Figure S10. MNase protection assay demonstrating the chromatin compaction of the target gene *ODC*.

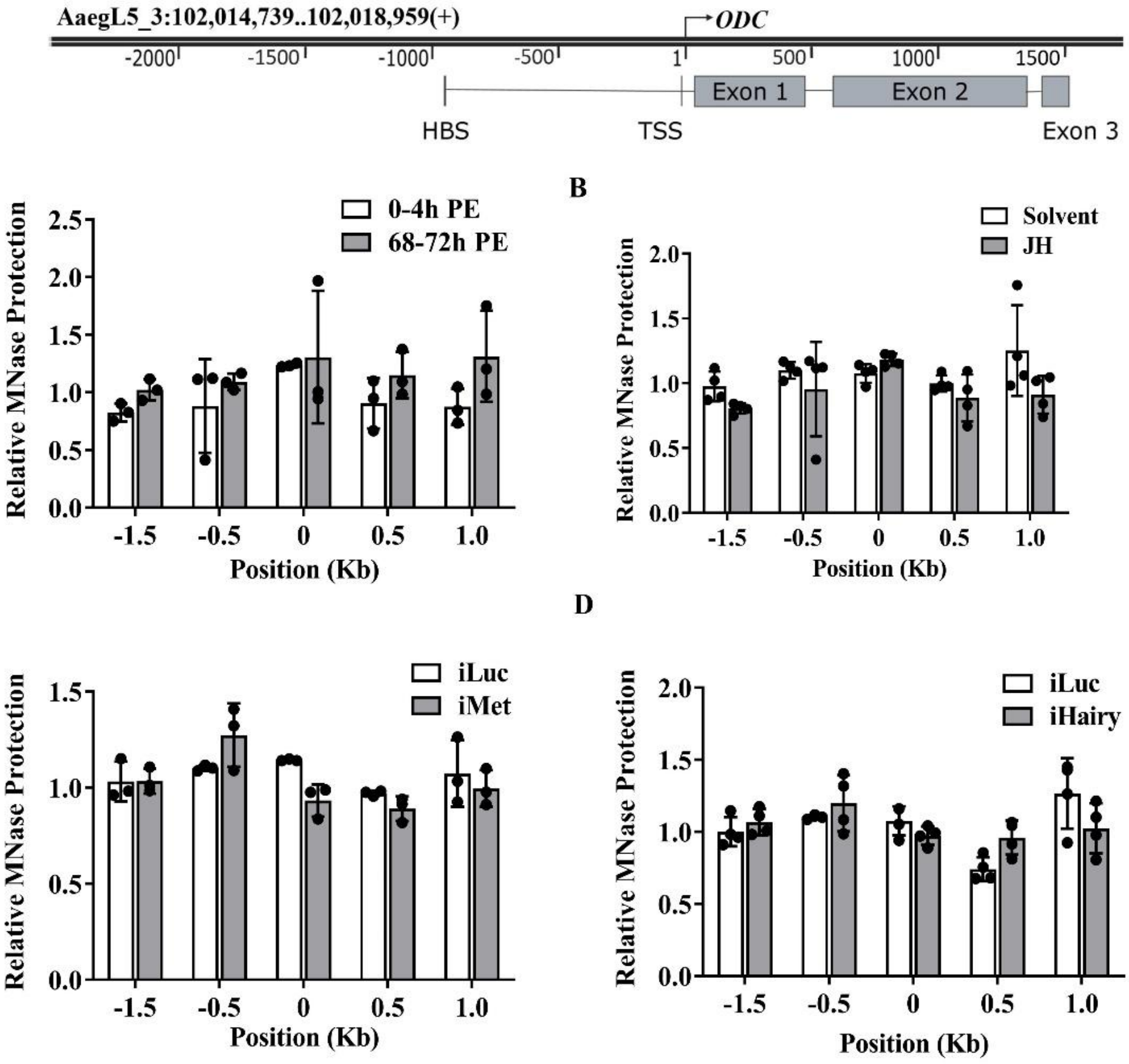

(A) No significant reduction in overall MNase sensitivity pattern is observed in 72 h PE samples or **(B)** JH-treated samples. **(C and D)** Knockdown of *Met* or *Kr-h1* does not impact the overall MNase digestion profile of *ODC*.

For ectopic JH treatment, sample collection was done 6 h post treatment. For knockdown, dsRNA injection was performed on 24 h PE adult female mosquitoes followed by sample collection 48 h post injection. Relative MNase protection level was calculated by comparing the level of digested samples with undigested controls. Error bars represent ± SD. Three biological replicates were performed for all experiments with similar results. Source data are provided in Supplementary Data 1.

Supplementary Figure S11. Histone H3 occupancy map of target gene *CSP*.

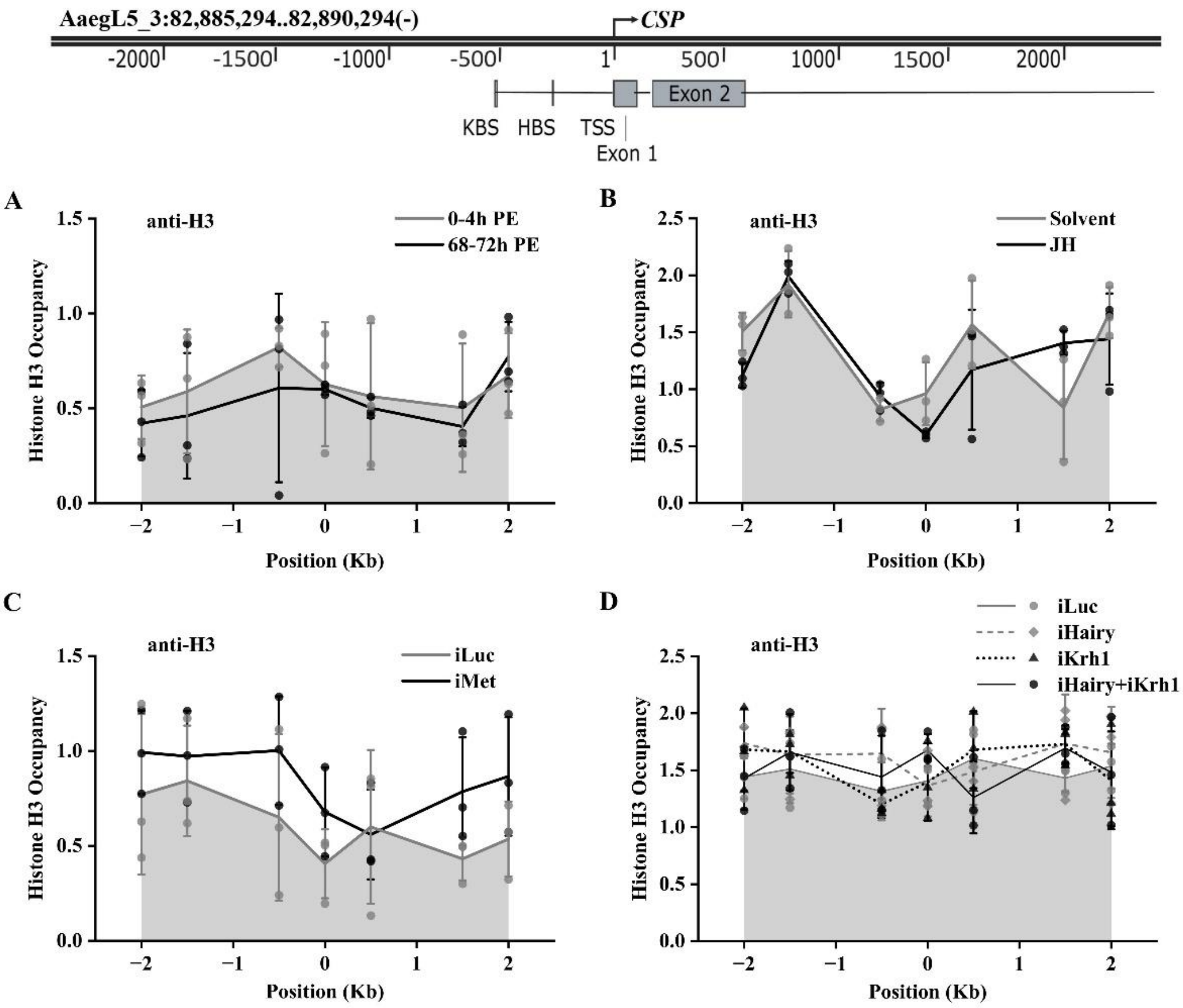

(A) PE reproductive maturation does not alter the overall histone H3 occupancy of the target gene, as evidenced by similar histone position profiles at 4 h and 72 h PE. **(B)** JH has no significant impact on the histone H3 positioning across the various regions of target gene loci. **(C)** Knockdown of *Met* results in no change in the histone positioning of the target gene *CSP*. **(D)** RNAi knockdowns of *Hairy* and *Kr-h1* individually (iHairy and iKr-h1, respectively) or simultaneously (iHairy+iKr-h1) do not alter the histone H3 occupancy map of the *CSP* gene. For ectopic JH treatment, sample collection was done 6 h post treatment. For knockdown, dsRNA injection was performed on 24 h PE adult female mosquitoes followed by sample collection 48 h post injection. Histone H3 occupancy was assayed for all experiments to check the effect of JH and its pathway components by ChIP-qPCR using an anti-H3 antibody. Histone H3 occupancy was calculated by normalizing the immunoprecipitation signal with the histone H3 occupancy level at the actin promoter. Error bars represent ± SD. Three biological replicates

were performed for all experiments with similar results. Source data are provided in Supplementary Data 1.

Supplementary Figure S12. Knockdown of transcriptional regulators compromises JH-Met induced chromatin modifications of *CSP*.

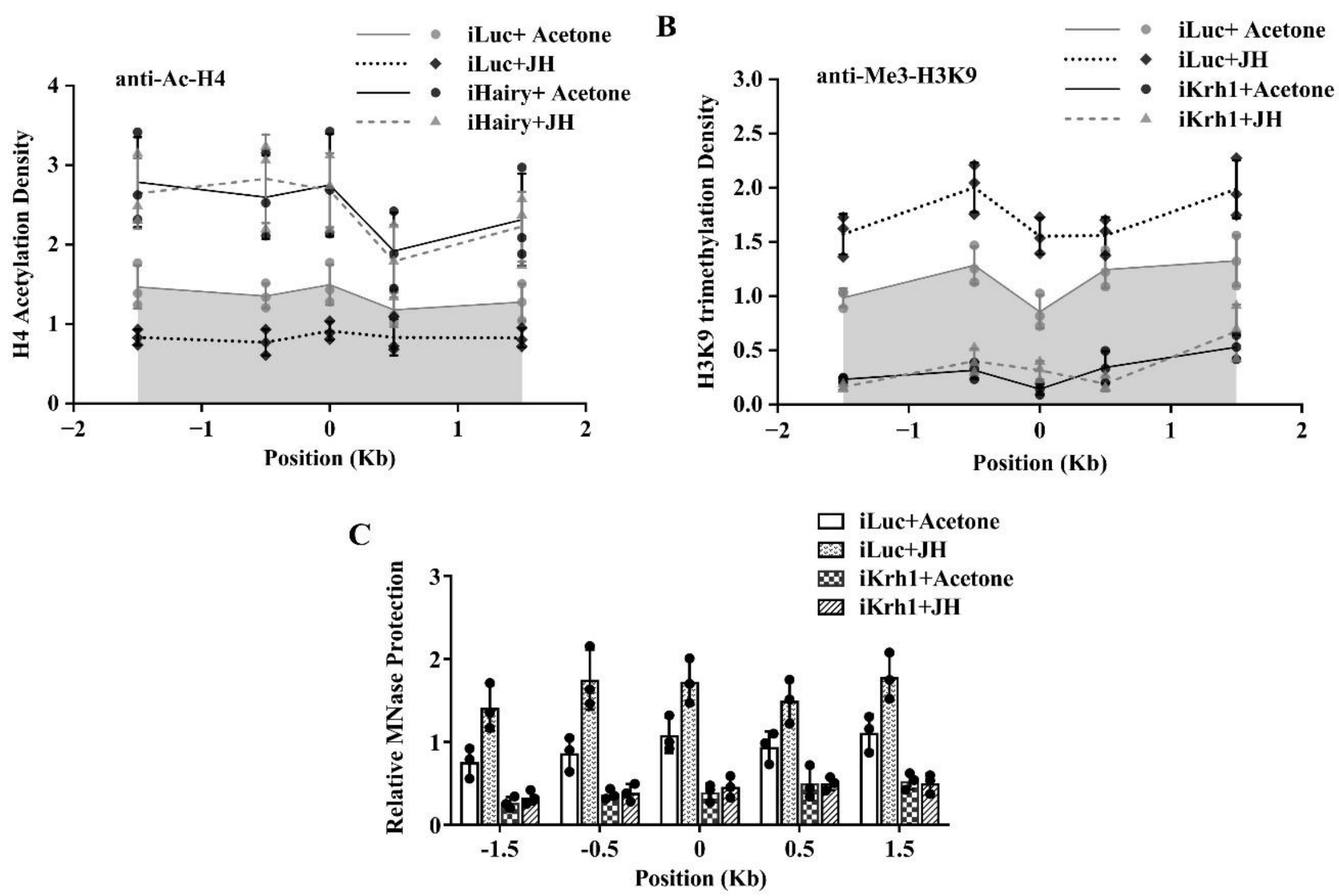

dsRNA mediated knockdown for *Hairy*, *Kr-h1* and *Luciferase* (control) was performed with adult female mosquitoes 12-16 h PE. JH or Acetone (control) treatment was done 48 h post injection followed by tissue collection for ChIP and MNase assay. **A)** Ectopic JH application does not alter the H4 acetylation density significantly at any locus of *CSP* gene in iHairy mosquitoes. However, JH induced significant H4 deacetylation in *CSP* gene in control iLuc mosquitoes. **B)** Ectopic JH application has no significant effect in H3K9 trimethylation density in *CSP* gene in iKr-h1 mosquitoes. However, JH induced significant H3K9 trimethylation in CSP gene in control iLuc mosquitoes. For (A & B), ChIP-qPCR was done using anti Ac-H4 and anti-Me3 H3K9 antibody. H4-Ac density and H3K9-Me3 density was calculated from ChIP samples by normalizing the immunoprecipitation signal first with the H4-Ac and H3K9-

Me3 level respectively at the actin promoter and then with the relative H3 level. **C)** Ectopic JH application does not impact the overall MNase sensitivity of *CSP* gene after dsRNA mediated knockdown of *Kr-h1*. In iLuc control, ectopic JH application significant increase relative MNase protection in all tested loci of *CSP* gene. Relative MNase protection level was calculated by comparing the level of digested samples with undigested controls.

dsRNA injection was performed on 24 h PE adult female mosquitoes followed by JH or acetone (control) treatment 48 h post injection. Samples were collected 6 h post JH treatment. Error bars represent ± SD. Three biological replicates were performed for all experiments with similar results. Source data are provided in Supplementary Data 1.

Supplementary Figure S13. Chromatin architecture of the target gene *Glutamine gamma glutamyl-transferase* (*GGG)*

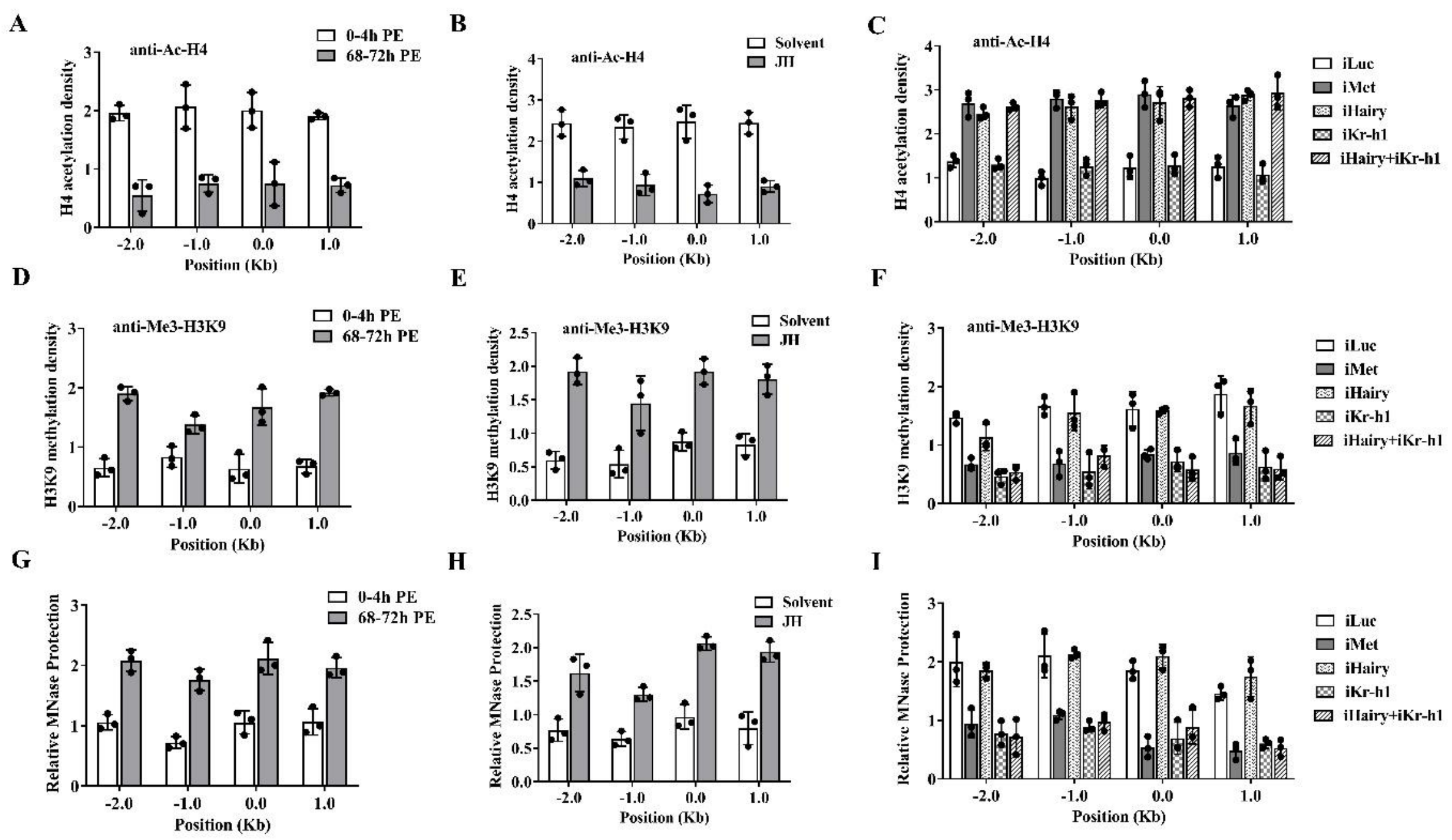

(A and B) Histone H4-Ac marks were significantly reduced at 72 h PE and after JH treatment at all the *GGG* gene loci tested. **(C)** RNAi knockdown of JH receptor *Met* and the downstream factor *Hairy* significantly increase the H4-Ac marks across different loci but remains unaltered in iKr-h1 samples. Double knockdown of *Hairy* and *Kr-h1* also shows increased H4-Ac marks

at all the *GGG* gene loci tested. **(D and E)** Histone H3k9-Me3 marks were significantly increased at 72 h PE and JH-treated samples. **(F)** iMet, iKr-h1, and iHairy+iKr-h1 samples significantly reduce H3K9-Me3 marks compared to iLuc samples. However, no significant change in the H3K9-Me3 profile of the *GGG* gene was observed in iHairy samples. **(G and H)** MNase sensitivity was significantly reduced in 72 h PE and JH-treated samples compared to controls throughout all the loci of *GGG*. **(I)** *Hairy* knockdown did not affect the compaction level of the *GGG* gene. Still, the knockdown of *Met*, *Kr-h1*, and double knockdown of *Hairy* and *Kr-h1* resulted in greater sensitivity of the target gene to MNase digestion.

For ectopic JH treatment, sample collection was done 6 h post treatment. For knockdown, dsRNA injection was performed on 24 h PE adult female mosquitoes followed by sample collection 48 h post injection. For graphs A-F, H4 acetylation density and histone H3K9-Me3 density was measured by ChIP assay using anti-Ac-H4 and anti-H3K9-Me3 antibody. H4 acetylation and H3K9 trimethylation density were calculated from ChIP samples by normalizing the immunoprecipitation signal first with the H4-Ac or H3K9-Me3 level at the actin promoter and then with the relative H3 level. For graphs G-I, the relative MNase protection level was calculated by comparing the level of digested samples with undigested controls. Error bars represent ± SD. Three biological replicates were performed for all experiments with similar results. Source data are provided in Supplementary Data 1.

Supplementary Figure S14. Confirmation of dsRNA mediated target gene knockdown by qPCR

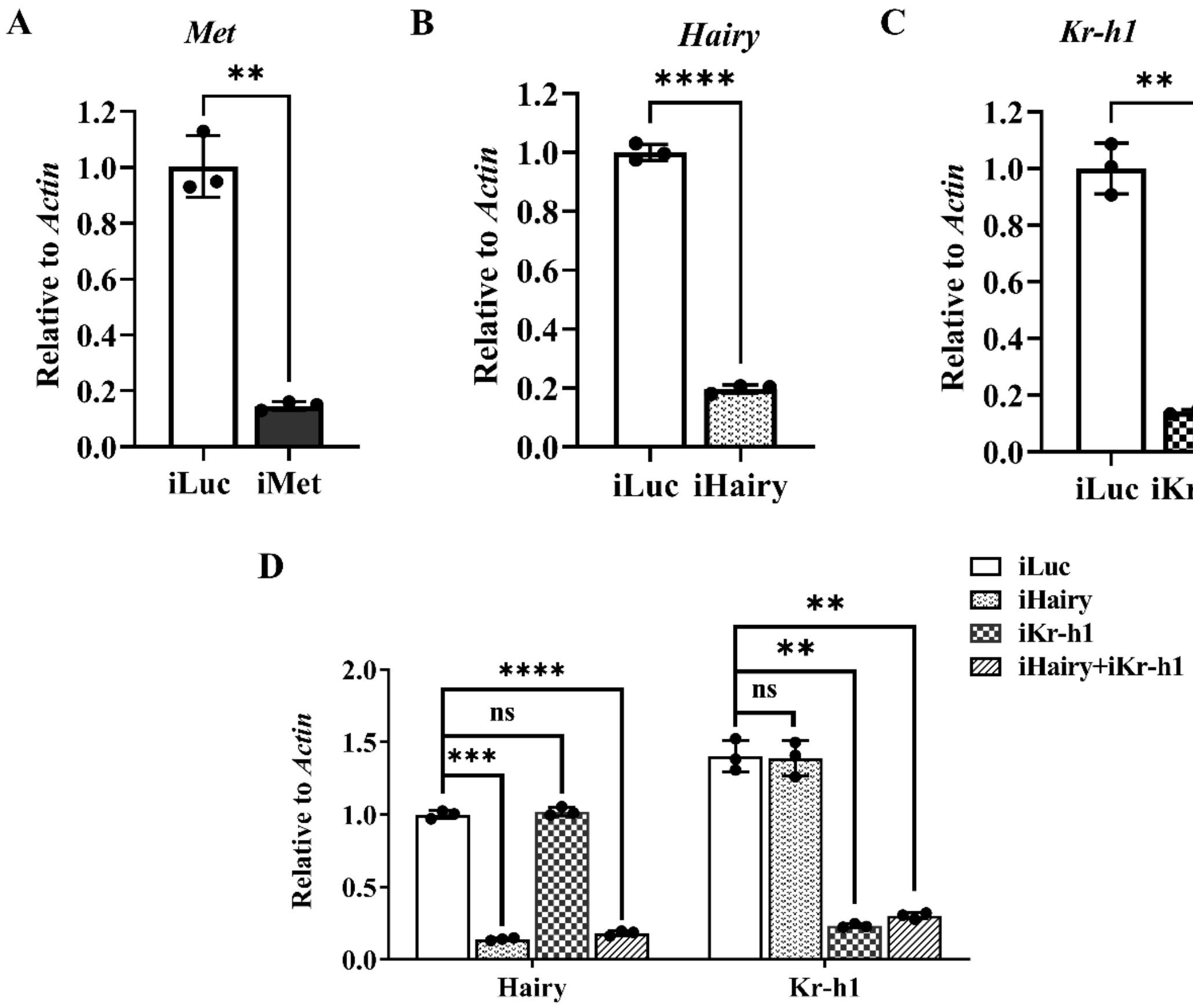

(A-C) Significant reduction in the levels of respective mRNA was observed post dsRNA treatment for *Met* (A), *Hairy* (B) and *Kr-h1* (C) in comparison to iLuc, confirming gene knockdown efficacy. **(D)** RNAi knockdown of either *Hairy* or *Kr-h1* significantly lowers mRNA levels of specific target gene. Double knockdown of *Hairy* and *Kr-h1* leads to significant reduction in the mRNA levels of both *Hairy* and *Kr-h1*.

Error bars represent ± SD. Three biological replicates were performed for all experiments with similar results. Source data are provided in Supplementary Data 1. (** p< 0.005; *** p< 0.0005; **** p< 0.00005; ns- not significant)

Supplementary Table S1. List of primers used

Primers for RNAi		
**Target_ID**	**Direction**	**Primer Sequence**
Kr-h1 *AAEL002390*	Forward	TAATACGACTCACTATAGGGCGCTTACCCAGCTATGCTTC
	Reverse	TAATACGACTCACTATAGGGCTCTTGAACGTTTCATCGCA
Met *AAEL025915*	Forward	TAATACGACTCACTATAGGGTCAATTTGTTCGACTCTGCG
	Reverse	TAATACGACTCACTATAGGGATACACAAAGTCGCCCGTTC
Hairy *AAEL027674*	Forward	TAATACGACTCACTATAGGGTGACCGTGAAACATTTGGAA
	Reverse	TAATACGACTCACTATAGGGCGGTCTCCAAGGTTTGTCAT
Luciferase *pGL3 Basic*	Forward	TAATACGACTCACTATAGGGGTCTGCCTAAAGGTGTCGCT
	Reverse	TAATACGACTCACTATAGGGGTGTTCGTCTTCGTCCCAGT

Primers for Q-PCR				
**Target_ID**	**Approximate position**	**Direction**	**Primer Sequence**	**Descriptors**
Ornithine decarboxylase (*ODC*) *AAEL000044*	-2 kb	Forward	TAAACATGACATTTGCGGTTTCCCA	ChIP assay
		Reverse	GTTCTTTGTTCTACCCGAGAATGC	
	-1.5 kb	Forward	GCCCAGAAGATCGAAAGATGAGA	ChIP and MNase protection assay
		Reverse	ACTAAAACCCATGGAAACCCCGA	
	-1 kb	Forward	GGCAGGCTTGAGGAAAATCG	ChIP assay
		Reverse	CACGTGAGCACCTTTCAATCG	
	-0.5 kb	Forward	CTTCCCTATACAAATGGGATTGCT	ChIP and MNase protection assay
		Reverse	TTGCACTATTCTAACACAAAAACCA	
	0 kb	Forward	CGACGACTCGGTTTCATCCC	ChIP and MNase protection assay
		Reverse	CCAGCGTATCCACTTCGGTC	
	0.5 kb	Forward	GTACCTGTGGCCTTAGTGGC	ChIP and MNase protection assay
		Reverse	ACCGATAGCGGATCAACACTC	
	1 kb	Forward	GTACGGGACGTTCTACAGCC	ChIP and MNase protection assay
		Reverse	TAGGTCGTCCCAAGATTGCC	
	1.5 kb	Forward	GGGAACTACTCGAAAAGGCATC	ChIP assay
		Reverse	GCAAGCACTTCGATCTGGGG	
		Forward	TGCACGCAAGCATAAGATTC	Expression Analysis
		Reverse	AACCGATAGCGGATCAACAC	
Dimethylaniline monooxygenase (*DMO*) *AAEL000834*	-4 kb	Forward	AGCGCGTCTTGATTTGTTATGA	ChIP assay
		Reverse	TAGAAATCCCTGTTGCCTGGG	
	-3 kb	Forward	ACGGGGGTGTACGGTATTACT	ChIP and MNase protection assay
		Reverse	AAGGATACAGGTCAAATGCGT	
	-2 kb	Forward	TACGATCCATGATACGCCCAAC	ChIP assay
		Reverse	AGCCCTATAAGAGCACGGAA	
	-1 kb	Forward	TTAACAACCCATTTCCAGGGC	ChIP and MNase protection assay
		Reverse	ATCGAGCAATGTGTCTGAACT	
	0 kb	Forward	AGTTACGCCCTGTTGATGGT	ChIP and MNase protection assay
		Reverse	ACCTCATTTCGTTGCTGGGT	
	0.5 kb	Forward	GAGTGCCCTACTTTCCCAGA	ChIP assay
		Reverse	ATGGTGAACACCCTCATCCATC	
	1 kb	Forward	AAACGCACCTACTGCGTGAT	ChIP and MNase protection assay
		Reverse	TCGTTGAACACCCACGTACC	

	2 kb	Forward	ACTGAGATGGAGGAACGCTG	ChIP and MNase protection assay
		Reverse	GTACCGATCCTCTTGCGGTC	
		Forward	ACCATACCCCAAAGTTGCCT	Expression Analysis
		Reverse	ACTAGCACGGTTTCACCAACA	
		Forward	CAGCTTGGTAAAACCGAAACAC	ChIP Assay_KBS
		Reverse	TGTTTCGATCCTTTTTGGTCTCA	
pGL3 Basic		Forward	TGGGCTGAATACAAATCACA	ChIP Assay_ KBS_ Control
		Reverse	CAACTCCGATAAATAACGCG	
Clip domain serine protease (*CSP*) *AAEL005093*	-2 kb	Forward	ATTTTGCACAAATTGTATACGGCT	ChIP assay
		Reverse	GGAACGATTAATTGTCTGCCAAAG	
	-1.5 kb	Forward	TTGTACCTCATCACCCACTTCA	ChIP and MNase protection assay
		Reverse	TTATGTCACTGGCATGATGGAA	
	-0.5 kb	Forward	TATGTGCACGTGATTCTAACGC	ChIP and MNase protection assay
		Reverse	AATGAATACCTACCTACACAAGTCG	
	0 kb	Forward	AACAATCGCCGATGAAACCG	ChIP and MNase protection assay
		Reverse	GATCCCAGCGCCTAATGGAC	
	0.5 kb	Forward	CTGGGTGGTGAAGAAACGGA	ChIP and MNase protection assay
		Reverse	AACTTCTCCGGCCACTGATG	
	1.5 kb	Forward	GACGTAGGATGGATTGCTCCC	ChIP and MNase protection assay
		Reverse	ATTCGATTCATAGGCGGATGGT	
	2 kb	Forward	CACTTCTCAATCAGCTCTTCTAGC	ChIP assay
		Reverse	TGAACTTGTCCGAAGGGTGC	
		Forward	GAAGTTATGGTTGCGGAGGA	Expression Analysis
		Reverse	TTCTAACACGCAAAGCATCG	
Matrix metallo-proteinase (*MMP*) *AAEL002655*	-1.5 kb	Forward	CGTTTCGTTTCGCATCAAACAGT	ChIP and MNase protection assay
		Reverse	GGGCGAGCGACTAAGGGTA	
	-1 kb	Forward	CGGAAAGATCCTGGACCGAC	ChIP and MNase protection assay
		Reverse	GAGGAAACAAATCGTGCCGC	
	0.5 kb	Forward	TGCTGGATTGGATGAGACCG	ChIP and MNase protection assay
		Reverse	GATGCACCGGATGGGTAGTC	
	1.5 kb	Forward	AAGTGGATCAGCACCACGAC	ChIP and MNase protection assay
		Reverse	GAGATCAGTTTCCCGGTGGG	
Thioester-containing protein 3 (Tep3) AAEL008607	-1.3 kb	Forward	TTTCATGCTAGTTTTTGTGATCCT	ChIP and MNase protection assay
		Reverse	AGTTCTCGTTTCTAATGAATTGGG	
	-0.3 kb	Forward	TTGGAAAAGCCGGTGCTGTA	ChIP and MNase protection assay
		Reverse	ACCACGCACATTCAACAAACA	
	0 kb	Forward	GAAGCAGCCTCACGATCAAG	ChIP and MNase protection assay
		Reverse	CGAGCGTACTTAGCGTAGCG	
	0.6 kb	Forward	CGATCCACATCCCTCGCTTC	ChIP and MNase protection assay
		Reverse	AGCCCAATGTGTGTGACTCT	
Glutamine gamma glutamyl-transferase (*GGG*)	-2 kb	Forward	TATTCAACCAATCAGTCACCTTGTG	ChIP and MNase protection assay
		Reverse	ATTTGCATTTAACCCGCAGATTGG	
	-1 kb	Forward	GTACGCAAACGTCAATCTAAAGCA	ChIP and MNase protection assay
		Reverse	TGCCTCCTAATGAAAGCCAAT	
	0 kb	Forward	AACATCCCGCGATTGACGAC	

AAEL006978		Reverse	ACTCAGTCGCCTCTCAAGTG	ChIP and MNase protection assay
	1 kb	Forward	GCTATTGAGCCTGAGTTTTCCTA	ChIP and MNase protection assay
		Reverse	TCTTCACCTTAAGATCACAGGTTT	
Met *AAEL025915*		Forward	TCACTTTCATCGGGGAGTTC	Knockdown
		Reverse	GAGGAGAAAGCGGAGGAAGT	
Hairy *AAEL027674*		Forward	CAAGTGCACATTTTGCCATC	Knockdown
		Reverse	GGATCAACTGAACGCTGGAT	
Kr-h1 *AAEL002390*		Forward	CAGGTCGTCAATCTGAGCAA	Knockdown
		Reverse	GGTCTGGTGGAAGTTCGTGT	
**Primers for Cloning**				
Dimethylaniline monooxygenase (*DMO*) *AAEL000834*		Forward	GCATGGTACCATGACATGGTAAGTGTGTCCAA	KBS Cloning
		Reverse	GCATAAGCTTCGGGTACGTTCAATAATACTTGTC	

Table S2. *p*-values for t-Test for ChIP and MNase protection assay for *ODC* gene in different treatment groups. [For all *p*-values < 0.001, it is represented as 0.001]

		-2 kb	-1.5 kb	-1 kb	-0.5 kb	-0 kb	0.5 kb	1 kb	1.5 kb
**H3 occupancy**	**0-4h vs 68-72h PE**	0.586	0.514	0.441	0.178	0.422	0.486	0.047	0.475
	**JH vs solvent**	0.198	0.065	0.320	0.217	0.171	0.334	0.508	0.098
	**iMet vs iLuc**	0.167	0.330	0.612	0.218	0.188	0.852	0.504	0.617
	**iHairy vs iLuc**	0.933	0.124	0.078	0.084	0.328	0.727	0.356	0.628
**H4 acetylation**	**0-4h vs 68-72h PE**	0.032	0.127	0.024	0.012	0.144	0.041	0.011	0.023
	**JH vs solvent**	0.002	0.002	0.028	0.041	0.048	0.003	0.021	0.014
	**iMet vs iLuc**	0.008	0.004	0.078	0.036	0.005	0.008	0.038	0.002
	**iHairy vs iLuc**	0.001	0.027	0.034	0.013	0.089	0.002	0.004	0.009
	**iLuc+JH vs iLuc+Acetone**		0.042		0.038	0.017	0.024	0.154	
	**iHairy+JH vs iHairy+Acetone**		0.219		0.711	0.287	0.280	0.573	
**H3K9 trimethylation**	**0-4h vs 68-72h PE**	0.808	0.614	0.963	0.251	0.459	0.421	0.929	0.312
	**JH vs solvent**	0.517	0.217	0.104	0.239	0.238	0.433	0.958	0.429
	**iMet vs iLuc**	0.578	0.423	0.714	0.853	0.569	0.036	0.924	0.102
	**iHairy vs iLuc**	0.240	0.042	0.596	0.631	0.212	0.126	0.179	0.568
**MNase protection**	**0-4h vs 68-72h PE**		0.057		0.493	0.822	0.273	0.121	
	**JH vs solvent**		0.047		0.436	0.072	0.348	0.197	
	**iMet vs iLuc**		0.931		0.278	0.041	0.163	0.540	
	**iHairy vs iLuc**		0.328		0.436	0.179	0.033	0.185	

Table S3. *p*-values for t-Test for ChIP and MNase protection assay for *DMO* gene in different treatment groups. [For all *p*-values < 0.001, it is represented as 0.001]

		**-4 kb**	**-3 kb**	**-2 kb**	**-1 kb**	**0 kb**	**0.5 kb**	**1 kb**	**2 kb**
**H3 occupancy**	**0-4h vs 68-72h PE**	0.664	0.631	0.763	0.049	0.109	0.933	0.231	0.864
	**JH vs solvent**	0.387	0.038	0.481	0.092	0.942	0.593	0.092	0.058
	**iMet vs iLuc**	0.612	0.487	0.132	0.789	0.664	0.813	0.084	0.239
	**iKr-h1 vs iLuc**	0.326	0.473	0.331	0.426	0.471	0.046	0.578	0.889
**H4 acetylation**	**0-4h vs 68-72h PE**	0.426	0.101	0.542	0.971	0.037	0.369	0.712	0.207
	**JH vs solvent**	0.523	0.741	0.261	0.404	0.497	0.067	0.843	0.433
	**iMet vs iLuc**	0.084	0.613	0.672	0.821	0.078	0.241	0.906	0.743
	**iKr-h1 vs iLuc**	0.224	0.017	0.547	0.330	0.689	0.276	0.866	0.738
**H3K9 trimethylation**	**0-4h vs 68-72h PE**	0.008	0.003	0.037	0.028	0.047	0.023	0.030	0.027
	**JH vs solvent**	0.021	0.002	0.016	0.034	0.078	0.040	0.004	0.036
	**iMet vs iLuc**	0.029	0.002	0.031	0.005	0.002	0.042	0.002	0.014
	**iKr-h1 vs iLuc**	0.023	0.004	0.316	0.037	0.018	0.021	0.020	0.033
	**iLuc+JH vs iLuc+Acetone**		0.048		0.042	0.050		0.123	0.018
	**iKr-h1+JH vs iKr-h1+Acetone**		0.341		0.153	0.112		0.087	0.129
**MNase protection**	**0-4h vs 68-72h PE**		0.003		0.006	0.033		0.049	0.039
	**JH vs solvent**		0.052		0.002	0.002		0.018	0.010
	**iMet vs iLuc**		0.007		0.024	0.004		0.042	0.047
	**iKr-h1 vs iLuc**		0.026		0.014	0.004		0.004	0.042
	**iLuc+JH vs iLuc+Acetone**	-	0.036	-	0.031	0.020	-	0.019	0.051
	**iKr-h1+JH vs iKr-h1+Acetone**		0.428		0.782	0.761		0.688	0.538

Table S4. ***p*****-values for t-Test for ChIP and MNase protection assay for *CSP* gene in different treatment groups.** [For all *p*-values < 0.001, it is represented as 0.001]

		-2 kb	-1.5 kb	-0.5 kb	0 kb	0.5 kb	1.5 kb	2 kb
**H3 occupancy**	**0-4h vs 68-72h PE**	0.631	0.694	0.511	0.834	0.757	0.618	0.596
	**JH vs solvent**	0.048	0.701	0.313	0.179	0.314	0.123	0.461
	**iMet vs iLuc**	0.437	0.515	0.349	0.141	0.922	0.178	0.236
	**iHairy vs iLuc**	0.118	0.694	0.348	0.799	0.643	0.318	0.696
	**iKr-h1 vs iLuc**	0.316	0.518	0.579	0.915	0.886	0.089	0.734
	**iHairy+iKr-h1 vs iLuc**	0.925	0.600	0.659	0.145	0.284	0.117	0.842
**H4 acetylation**	**0-4h vs 68-72h PE**	0.030	0.032	0.024	0.048	0.268	0.033	0.018
	**JH vs solvent**	0.024	0.057	0.019	0.023	0.040	0.037	0.009
	**iMet vs iLuc**	0.008	0.018	0.017	0.022	0.024	0.028	0.038
	**iHairy vs iLuc**	0.001	0.021	0.042	0.027	0.038	0.033	0.001
	**iKr-h1 vs iLuc**	0.038	0.733	0.951	0.493	0.220	0.050	0.498
	**iHairy+iKr-h1 vs iLuc**	0.005	0.048	0.002	0.025	0.024	0.003	0.004
	**iLuc+JH vs iLuc+Acetone**		0.036	0.047	0.049	0.124	0.081	
	**iHairy+JH vs iHairy+Acetone**		0.784	0.526	0.889	0.758	0.816	
**H3K9 trimethylation**	**0-4h vs 68-72h PE**	0.022	0.084	0.014	0.048	0.016	0.036	0.041
	**JH vs solvent**	0.006	0.045	0.028	0.052	0.017	0.012	0.027
	**iMet vs iLuc**	0.012	0.004	0.019	0.032	0.048	0.007	0.011
	**iHairy vs iLuc**	0.371	0.742	0.620	0.821	0.836	0.455	0.479
	**iKr-h1 vs iLuc**	0.034	0.006	0.036	0.059	0.034	0.006	0.022
	**iHairy+iKr-h1 vs iLuc**	0.008	0.003	0.018	0.013	0.047	0.003	0.071
	**iLuc+JH vs iLuc+Acetone**		0.036	0.019	0.014	0.125	0.048	
	**iKr-h1+JH vs iKr-h1+Acetone**		0.023	0.389	0.086	0.278	0.401	
**MNase protection**	**0-4h vs 68-72h PE**		0.038	0.004	0.015	0.005	0.047	
	**JH vs solvent**		0.084	0.035	0.024	0.039	0.011	
	**iMet vs iLuc**		0.043	0.028	0.022	0.040	0.021	
	**iHairy vs iLuc**		0.489	0.804	0.539	0.754	0.812	
	**iKr-h1 vs iLuc**		0.026	0.014	0.044	0.029	0.006	
	**iHairy+iKr-h1 vs iLuc**		0.136	0.018	0.039	0.003	0.047	
	**iLuc+JH vs iLuc+Acetone**		0.048	0.028	0.030	0.044	0.043	
	**iKr-h1+JH vs iKr-h1+Acetone**		0.489	0.836	0.520	0.981	0.728	

Table S5. *p*-values for t-Test for ChIP and MNase protection assay for *MMP* gene in different treatment groups. [For all *p*-values < 0.001, it is represented as 0.001]

		-1.5 kb	-1 kb	0.5 kb	1.5 kb
**H4 acetylation**	**0-4h vs 68-72h PE**	0.002	0.003	0.004	0.008
	**JH vs solvent**	0.025	0.003	0.033	0.039
	**iMet vs iLuc**	0.003	0.002	0.011	0.012
	**iHairy vs iLuc**	0.009	0.004	0.039	0.047
**H3K9 trimethylation**	**0-4h vs 68-72h PE**	0.136	0.151	0.770	0.189
	**JH vs solvent**	0.936	0.148	0.397	0.296
	**iMet vs iLuc**	0.647	0.199	0.564	0.912
	**iHairy vs iLuc**	0.578	0.631	0.766	0.352
**MNase treatment**	**0-4h vs 68-72h PE**	0.436	0.031	0.069	0.325
	**JH vs solvent**	0.211	0.031	0.498	0.124
	**iMet vs iLuc**	0.357	0.922	0.330	0.085
	**iHairy vs iLuc**	0.093	0.248	0.857	0.223

Table S6. *p*-values for t-Test for ChIP and MNase protection assay for *TEP3* gene in different treatment groups. [For all *p*-values < 0.001, it is represented as 0.001]

		-1.5 kb	-0.5 kb	0 kb	0.5 kb
**H4 acetylation**	**0-4h vs 68-72h PE**	0.878	0.344	0.671	0.184
	**JH vs solvent**	0.397	0.660	0.384	0.835
	**iMet vs iLuc**	0.412	0.589	0.436	0.574
	**iKr-h1 vs iLuc**	0.650	0.633	0.489	0.374
**H3K9 trimethylation**	**0-4h vs 68-72h PE**	0.004	0.028	0.001	0.003
	**JH vs solvent**	0.019	0.011	0.008	0.007
	**iMet vs iLuc**	0.004	0.016	0.021	0.025
	**iKr-h1 vs iLuc**	0.004	0.009	0.007	0.008
**MNase treatment**	**0-4h vs 68-72h PE**	0.004	0.001	0.002	0.001
	**JH vs solvent**	0.048	0.004	0.002	0.011
	**iMet vs iLuc**	0.024	0.003	0.040	0.036
	**iKr-h1 vs iLuc**	0.026	0.014	0.028	0.043

Table S7. *p*-values for t-Test for ChIP and MNase protection assay for *GGG* gene in different treatment groups. [For all *p*-values < 0.001, it is represented as 0.001]

		-2 kb	-1 kb	0 kb	1 kb
**H4 acetylation**	**0-4h vs 68-72h PE**	0.009	0.038	0.026	0.006
	**JH vs solvent**	0.005	0.006	0.005	0.007
	**iMet vs iLuc**	0.006	0.002	0.002	0.005
	**iHairy vs iLuc**	0.003	0.003	0.009	0.003
	**iKr-h1 vs iLuc**	0.589	0.156	0.783	0.361
	**iHairy+iKr-h1 vs iLuc**	0.003	0.001	0.002	0.009
**H3K9 trimethylation**	**0-4h vs 68-72h PE**	0.001	0.029	0.014	0.002
	**JH vs solvent**	0.002	0.033	0.008	0.008
	**iMet vs iLuc**	0.002	0.004	0.022	0.036
	**iHairy vs iLuc**	0.123	0.689	0.828	0.470
	**iKr-h1 vs iLuc**	0.002	0.017	0.026	0.008
	**iHairy+iKr-h1 vs iLuc**	0.001	0.003	0.011	0.019
**MNase treatment**	**0-4h vs 68-72h PE**	0.001	0.004	0.011	0.016
	**JH vs solvent**	0.013	0.004	0.001	0.005
	**iMet vs iLuc**	0.044	0.032	0.003	0.004
	**iHairy vs iLuc**	0.626	0.942	0.210	0.255
	**iKr-h1 vs iLuc**	0.027	0.033	0.006	0.004
	**iHairy+iKr-h1 vs iLuc**	0.023	0.048	0.009	0.003
