# Supplementary material for: Juvenile hormone-mediated chromatin remodelling by cumulative action of downstream transcription factors, Hairy and Krüppel homolog 1
Source: Commun Biol. 2026 May 26;9:1214. doi: 10.1038/s42003-026-10327-4 (PMC13586389; doi:10.1038/s42003-026-10327-4)
Supplement: Supplementary file 3 [file 42003_2026_10327_MOESM3_ESM.pdf]

Description of Additional Supplementary Files

File name- Supplementary Data 1

Description – Numerical source data for graphs can be found in Supplementary Data 1

File name: Supplementary Data 2

Description: dsRNA sequences for knockdown of target genes

File name: Supplementary Data 3

Description: Sequence of plasmid generated for the study
