# Supplementary material for: Juvenile hormone-mediated chromatin remodelling by cumulative action of downstream transcription factors, Hairy and Krüppel homolog 1
Source: Commun Biol. 2026 May 26;9:1214. doi: 10.1038/s42003-026-10327-4 (PMC13586389; doi:10.1038/s42003-026-10327-4)
Supplement: Supplementary file 5 [file 42003_2026_10327_MOESM5_ESM.docx]

>dsRNA for *Luciferase* (From the backbone of plasmid pGL3 Basic)

TAATACGACTCACTATAGGGGTCTGCCTAAAGGTGTCGCTCTGCCTCATAGAACTGCCTGCGTGAGATTCTCGCATGCCAGAGATCCTATTTTTGGCAATCAAATCATTCCGGATACTGCGATTTTAAGTGTTGTTCCATTCCATCACGGTTTTGGAATGTTTACTACACTCGGATATTTGATATGTGGATTTCGAGTCGTCTTAATGTATAGATTTGAAGAAGAGCTGTTTCTGAGGAGCCTTCAGGATTACAAGATTCAAAGTGCGCTGCTGGTGCCAACCCTATTCTCCTTCTTCGCCAAAAGCACTCTGATTGACAAATACGATTTATCTAATTTACACGAAATTGCTTCTGGTGGCGCTCCCCTCTCTAAGGAAGTCGGGGAAGCGGTTGCCAAGAGGTTCCATCTGCCAGGTATCAGGCAAGGATATGGGCTCACTGAGACTACATCAGCTATTCTGATTACACCCGAGGGGGATGATAAACCGGGCGCGGTCGGTAAAGTTGTTCCATTTTTTGAAGCGAAGGTTGTGGATCTGGATACCGGGAAAACGCTGGGCGTTAATCAAAGAGGCGAACTGTGTGTGAGAGGTCCTATGATTATGTCCGGTTATGTAAACAATCCGGAAGCGACCAACGCCTTGATTGACAAGGATGGATGGCTACATTCTGGAGACATAGCTTACTGGGACGAAGACGAACACCCCTATAGTGAGTCGTATTA

> dsRNA for *Methoprene tolerant (AAEL025915)*

TAATACGACTCACTATAGGGTCAATTTGTTCGACTCTGCGGTGTCAGCTCCATCGACGAGTCGAACATCCAGCGGAACGGAGACGAGCGCGGAGGAGCAACAGCGAAAGTCGCAGGACGAGGAAGACGAGATTGATCGCAAGTTGCGACAGGACAGGAGGAAGTTCACGTTAAGGATCGCCAGGGCAGGACCTCGATCTGAGCCAACAGCATACGAGCTAGTCACGATAGATGGATTCTTCAGGCGTGCGGATGCCGCTCCACGAGGAGAACGACCAAGTGGTCCCTCGGGTCTGCAGCTCTTGAGGAGGGCACGCGGCCGCGACGATGGGATTACACTGCAAAGCATTAATGGGAATGATATTGTTCTGGTAGCAGTGGCTCGCGTGCAAAAGGTGCCAACGATCTGTGATCGATTGATTGAGGCTTGCAGATATGAGTACAAAACGCGTCACCTGATCGACGGCAGGATAGTGCAATGTGATCACCGGATATCGGTCGTGGCTGGATACTTGACGACGGAAGTTTCCGGGCTGTCACCGTTTACCTTCATGCACAAGGACGACGTCCGATGGGTTATCGTGGCCCTTCGGCAGATGTACGATTACAGTCAGAACTACGGTGAGTCCTGCTACCGATTGATGACACGAACGGGCGACTTTGTGTATCCCTATAGTGAGTCGTATTA

> dsRNA for *Hairy (AAEL027674)*

TAATACGACTCACTATAGGGAAATGACCGTGAAACATTTGGAAAATCTGCAGCGACAGCAAAATGCTATGTCACAGGCGACAGATCCAAATGTTATGAACAAGTTCAAAGCTGGATTCAACGAGTGCGCCCAGGAAGTTAGCAGGTTTCCGGATATTGATCCAATGACCCGCCGGCGTTTGTTAGCTCATTTGAGCAACTGTATCAATGGAGTCAAATCTGATCTTCCAAAATCACGTCAATCGGCAGTCCAAGTGCACATTTTGCCATCGCCTCCCAGTTCACCAGAGCAAGATCATCATCCTCAACCTCATCCAGCTCAGATCAACGCAGTGCAGACCGGTAACGGAGTGTACTTCACTAATAGTATCGGATCCAGCGTTCAGTTGATCCCTACTAAACTTCCGAACGGAAGCATGGCTTTTGTTCTGCCGCAAGCCATTTCTGCAGCGACAGCTCCTGTTCCAATGCTAGTTCCGATTCCCAGTCGTACTGCATCTACCGGCTCAGCAGCATCCAGCCATTCGTCTTCGTCCGCGTACGATCGAGTTCCCCAAGAACACTCTACCTCGCCATATCACGCACCACCAAGTCCAGCCAACTCTAACTATGAACCCATGGACTGCAAACCCTCAGTTTTCCAAGAGCAACAGCAATACCTCCACTCGCAACAGATGCGGTACCAGCAACAGCAACCTCAGTCCCAATCGAGAGCCTACAGTCCCGACAGTCCGTTATCCTTGGTCATGAAAAAACCCTACCATGATCAGAACCAAGACGATGACAAACCTTGGAGACCGCCCTATAGTGAGTCGTATTA

> dsRNA for *Krüppel homolog-1 (AAEL002390)*

TAATACGACTCACTATAGGGCGCTTACCCAGCTATGCTTCAACGCAACGAACGACCATTCCCAGTCGCAGCCCCAACAGCAGCAGCAGCAGCAACAGCAATCGCTTGACCAATCCCTTCACCATCAGCAACAGCAACAGCATAGGTTCCAGCAGCAGGTCGTCAATCTGAGCAACACCCAAGGCAACATGCAGTCACAGATGGGCCAGCAGCATCCCAGCGCACCCTACACGAACTTCCACCAGACCAATAATGGTGGACTCGTGCCGACGACCATACCGCAGACCAGCCCTCCCCCGGCCGGACCTGCCAAGTTCCGCTGCGAACAGTGCAACATCAACTTCGGTAGCAAAAGTGCCCATACCAGTCACATGAAATCTCACGCCAAACAGCAGCAAATGGTAGCGGAGAGCAAGGGTGGAGTCAGTGCCAAGGGATTGCTCAACGGACCGGCCACTCCGGCCGACCCGTACCAGTGCGATGTGTGCAAGAAAACTTTTGCCGTTCCGGCCCGGCTTGTGCGGCACTACCGGACACATACCGGTGAACGCCCGTTCGAGTGCGAGTTCTGCCATAAGATGTTTAGCGTCAAGGAGAACCTGCAGGTTCACCGGCGGATCCACACCAAGGAACGGCCGTACAAGTGCGACATCTGCGAGCGAGCGTTTGAACACAGCGGAAAACTGCACAGGCACATGCGGATCCATACGGGTGAACGACCACACAAGTGCAACGTGTGCGGAAAGACGTTCATCCAGTCGGGTCAACTAGTCATCCACATGCGGACCCATACCGGTGAGAAACCGTACAAATGTCCGGTAGAGGGTTGCGGAAAGGGCTTCACGTGCTCGAAACAGTTGAAAGTGCACTCGCGGACTCACACCGGTGAGAAGCCGTACCATTGTGATATTTGCTTCCGGGACTTTGGCTATAACCATGTGCTGAAGTTGCACAGGGTACAGCACTACGGGTCGAAGTGCTACAAATGTACCATATGCGATGAAACGTTCAAGAGCCCTATAGTGAGTCGTATTA
